# Interacting Temperature, Nutrients and Zooplankton Grazing Control Phytoplankton Size-Abundance Relationships in Eight Swiss Lakes

**DOI:** 10.3389/fmicb.2019.03155

**Published:** 2020-01-22

**Authors:** Francesco Pomati, Jonathan B. Shurin, Ken H. Andersen, Christoph Tellenbach, Andrew D. Barton

**Affiliations:** ^1^Aquatic Ecology, Eawag: Swiss Federal Institute of Aquatic Science and Technology, Dübendorf, Switzerland; ^2^Institute of Integrative Biology, ETH-Zurich, Zurich, Switzerland; ^3^Department of Ecology Behavior and Evolution, University of California, San Diego, La Jolla, CA, United States; ^4^Centre for Ocean Life, DTU Aqua, Technical University of Denmark, Lyngby, Denmark; ^5^Scripps Institution of Oceanography, La Jolla, CA, United States

**Keywords:** size spectra, bottom–up and top–down controls, main effects and interactions, non-linear effects, random forests, eutrophication, climate change

## Abstract

Biomass distribution among size classes follows a power law where the Log-abundance of taxa scales to Log-size with a slope that responds to environmental abiotic and biotic conditions. The interactions between ecological mechanisms controlling the slope of locally realized size-abundance relationships (SAR) are however not well understood. Here we tested how warming, nutrient levels, and grazing affect the slope of phytoplankton community SARs in decadal time-series from eight Swiss lakes of the peri-alpine region, which underwent environmental forcing due to climate change and oligotrophication. We expected rising temperature to have a negative effect on slope (favoring small phytoplankton), and increasing nutrient levels and grazing pressure to have a positive effect (benefiting large phytoplankton). Using a random forest approach to extract robust patterns from the noisy data, we found that the effects of temperature (direct and indirect through water column stability), nutrient availability (phosphorus and total biomass), and large herbivore (copepods and daphnids) grazing and selectivity on slope were non-linear and interactive. Increasing water temperature or total grazing pressure, and decreasing phosphorus levels, had a positive effect on slope (favoring large phytoplankton, which are predominantly mixotrophic in the lake dataset). Our results therefore showed patterns that were opposite to the expected long-term effects of temperature and nutrient levels, and support a paradigm in which (i) small phototrophic phytoplankton appear to be favored under high nutrients levels, low temperature and low grazing, and (ii) large mixotrophic algae are favored under oligotrophic conditions when temperature and grazing pressure are high. The effects of temperature were stronger under nutrient limitation, and the effects of nutrients and grazing were stronger at high temperature. Our study shows that the phytoplankton local SARs in lakes respond to both the independent and the interactive effects of resources, grazing and water temperature in a complex, unexpected way, and observations from long-term studies can deviate significantly from general theoretical expectations.

## Introduction

**FIGURE 1:**
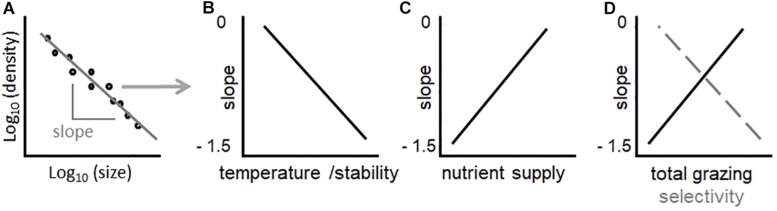
The SAR [local size-density relationship (Reuman et al., 2008) – **A**], and relative hypotheses of how changes in environmental conditions should influence its slope in lake phytoplankton communities **(B–D)**. In **(D)**, the black solid line depicts the predicted effect of total grazing pressure, while the gray dashed lines the effect of zooplankton selectivity (ratio of abundance between copepods and daphnids). Note that hypothesized trends in **(B–D)** are linear to simplify concepts. While a linear fit in **(A)** is a prerequisite for power law scaling, identifying the shape of relationships in **(B–D)** is a goal of this study.

In aquatic ecosystems, the size of planktonic organisms is a key determinant of community structure and food-web dynamics (Marañón, 2015). The relationship between size and abundance emerges from organism traits and ecological interactions, and describes how biomass is partitioned among the biota within an ecosystem (Sprules et al., 2016). Smaller phytoplankton are typically more numerous than larger phytoplankton in freshwater (Sprules and Munawar, 1986; Sprules et al., 1991; Gaedke et al., 2004) and marine ecosystems (Sheldon et al., 1972; Rodriguez and Mullin, 1986; Cavender-Bares et al., 2001; Huete-Ortega et al., 2014; Marañón, 2015). This negative relationship between abundance and body size is often called the size spectrum, and can be generally described as:

$$\mathrm{Abundance}=a\cdot \mathrm{Size}{}^{b}$$

where *a* is a constant and *b* is the power spectral slope. Observations from fresh and marine waters indicate that community size spectra in aquatic ecosystems tend to conform to the above power law, and exponent *b* is often close to −1 (larger cells are scarce relative to smaller cells) (Cavender-Bares et al., 2001; Gaedke et al., 2004; Marañón, 2015; Sprules et al., 2016). A less negative slope indicates a more even distribution of large and small cells. Alternatively, the size-abundance relationship (SAR) can be depicted by plotting the density of taxa as a function of their biovolume in a log–log space (Figure 1A). In phytoplankton, transient states or variations in phytoplankton SARs can have significant implications for aquatic food-webs and biogeochemical cycling. For example, communities with a greater proportion of larger phytoplankton cells are generally associated with algal blooms and lower biomass transfer to the herbivore food-chain (Behrenfeld and Boss, 2013; Yvon-Durocher et al., 2015; Cloern, 2018).

Slopes of size spectra and SARs are strong indicators of environmental and biotic impacts on community structure, particularly temperature, resource supply, and size-selective grazing. Competition theory in ecology predicts that temperature should favor small organismal size relative to large, also in phytoplankton (Reuman et al., 2014). This hypothesis is consistent with experimental evidence that, under higher temperature, smaller organisms maintain high metabolic rates because they are more efficient in resource uptake (due to more favorable surface to volume ratio) (Atkinson et al., 2003; Andersen et al., 2016). Increasing temperature of water should therefore decrease the slope (i.e., more negative values) of the phytoplankton community SAR, by increasing the prevalence of small taxa relative to large ones (Winder et al., 2009; Yvon-Durocher et al., 2011; Reuman et al., 2014; Marañón, 2015; Rasconi et al., 2015) (Figure 1B). Warming, however, can also have indirect effects on phytoplankton community structure. A strong effect of warming on phytoplankton community composition may be related to changes in thermal stratification and vertical mixing (Livingstone, 2003; Winder and Sommer, 2012; Yankova et al., 2017). For example, mixing processes determine changes in resource availability: enhanced or prolonged stratification of the water column due to warming can suppress the upward flux of nutrients from deep-waters through vertical mixing, resulting in more nutrient-depleted surface waters (Yankova et al., 2017; Lepori et al., 2018). Smaller organisms, which possess advantageous surface to volume ratio for nutrient uptake, should dominate in nutrient depleted environments (Marañón, 2015). Increasing temperature therefore has direct and indirect effects on phytoplankton size distributions, which should all combine to promote the dominance of small taxa and decrease (toward more negative values) the slope of SAR (Figure 1B).

Resource supply has a key role in determining the slope of SARs. Oligotrophic regions show steeper (more negative) slopes, while nutrient rich or eutrophic environments present flatter (less negative) slopes (Gaedke et al., 2004; Barton et al., 2013; Marañón, 2015; Guiet et al., 2016; Sprules et al., 2016). In principle, smaller phytoplankton should always outcompete larger ones, not only under oligotrophic conditions, since they have relatively high nutrient specific uptake affinity and growth rates (Edwards et al., 2012). In the presence of grazers, however, an increase in abundance of small phytoplankton is rapidly balanced by grazing, leaving excess nutrients available for larger phytoplankton forms, which gain an advantage through the lagged growth and grazing of their smaller competitors (Stibor et al., 2004; Ward et al., 2012; Barton et al., 2013; Marañón, 2015). The grazers of small phytoplankton are mostly microzooplankton (flagellates, dinoflagellates, ciliates and rotifers), which have generation times of the same scale of their prey, while larger phytoplankton have larger, slower-growing macrozooplankton predators such as copepods or cladocerans (Hansen et al., 1997; Sommer et al., 2001; Stibor et al., 2004; Wollrab and Diehl, 2015), whose generation times are orders of magnitude longer. Large phytoplankton are therefore more likely to outgrow their grazers under conditions of high nutrient supply, favoring larger phytoplankton taxa (Cloern, 2018). This implies that adding nutrients makes the SAR slope more positive (Armstrong, 1994; Stibor et al., 2004; Ward et al., 2012; Marañón, 2015) (Figure 1C). Light is also an important factor: light absorption decreases in larger cells because self-shading by pigment molecules (the package effect) increases with size, especially under light limitation, when intracellular pigment concentrations are higher (Finkel et al., 2010). Small cell size in phytoplankton is therefore advantageous in resource poor environments, where light and/or nutrient availability is low (Figure 1C). The ability to rapidly uptake and store nutrients may favor large cells in fluctuating environments, though it is not clear how this would uniformly affect SARs (Verdy et al., 2009; Bonachela et al., 2011).

Consensus on the magnitude and variability of the slope of SARs, as well as the causes of this variability, remains elusive. We expect variability in the SAR to occur in natural systems due to changes in environmental and ecological conditions, and due to seasonal succession or disturbance, but the mechanisms may be complex and multivariate. For example, in a set of experimental aquatic ecosystems, warming of ∼4°C initially increased the steepness of the phytoplankton community size spectra slope by increasing the prevalence of small organisms (Yvon-Durocher et al., 2011). These results have been attributed to greater competition among phytoplankton for limiting resources due to temperature-induced increases in metabolic rates. The effect of temperature was, however, reversed over the long term in the same experiment when warming was associated with dominance of large algal species (Yvon-Durocher et al., 2015). This pattern was interpreted as emerging from trophic interactions, with warming favoring taxa that were more resistant to grazing (larger cell size and/or colony formation), suggesting the importance of interacting bottom–up and top–down controls on size distributions. Despite the above evidence that temperature, resource supply and zooplankton grazing impact size distributions, unequivocal evidence linking main effects and interactions to changes in slopes from field observations is lacking. The shape of such relationships are also largely unknown. This gap may be due, in part, to the difficulty of disentangling the effects of these co-variating drivers, and to data limitations.

Here, we use long term phytoplankton community datasets from eight lakes in Switzerland, sampled monthly for decades, and spanning a range of environmental and ecosystem attributes (Table 1). We quantify the temporal variability in phytoplankton SARs, and test the above hypotheses about how abiotic and biotic factors control the relative abundances of different phytoplankton sizes. We examine variations through time in SARs and its drivers instead of the more commonly calculated size spectra, where the abundance of organisms in log-spaced bins is added together (White et al., 2008). We preferred the SAR approach because we could retain all individual data points from all taxa and adopt a robust slope estimation procedure based on bootstrapping of the species pool. The eight Swiss lakes provide an ideal setting for this study because of the quantity and quality (standardization) of paired biological and environmental observations (Table 1), and the lakes’ well-known history of eutrophication, re-oligotrophication, and climate change (Livingstone, 2003; Anneville et al., 2004; Pomati et al., 2012; Monchamp et al., 2018). We explore the extent of variation in phytoplankton SARs across ecosystems of contrasting conditions. This included the investigation of the relative importance and interactions among in-lake drivers such as nutrients, grazing and temperature, and seasonal and inter-annual ecosystem changes. For data analysis, we used a non-parametric machine learning approach (random forests), to find generalizable predictive patterns in notoriously noisy data. Random forests (RF) allowed us to overcome the most important constraints of traditional statistical approaches: *a priori* specification of (i) functional forms, (ii) interactions, and (iii) error distributions (Thomas et al., 2018). We expect that the marked natural and anthropogenic disturbances, particularly in temperature and phosphorous supply, induced variations in abundances between different size classes, which would allow us to quantitatively link changes in phytoplankton SARs to environmental drivers.

**TABLE 1:** Lake and plankton metadata.

				Phytoplankton				Chemistry				Zooplankton			Number of matching dates (*n*)	Previous studies
Lake	Abbreviation	Volume (km^3^)	Depth at sampling site (m)	Time span	Effective dates (*n*)	Depth range (m)	Number of taxa (unassigned)	Time-span	Effective dates (*n*)	Depth range (m)	P-PO4 level (μg/L)**	Time-span	Effective dates (*n*)	Depth range (m)		
Walensee	WA	2.5	144	1972–2000	350	0–20	158 (5)	1980–2000	261	0–144	2	1980–2000	249	0–144	243	Anneville et al., 2004, 2005
Upper Lake Zurich	UZ	0.6	36	1972–2000	349	0–20	174 (6)	1980–2000	253	0–36	5	1980–2000	251	0–36	253	Anneville et al., 2004, 2005
Lake Lucerne*	LU	11.8	110 (150)*	1968–2014	738	0–20 (30)*	384 (32)	1968–2004	327	0–110 (150)*	9	1975–2014	650	0–110 (150)*	202	Finger et al., 2013
Lower Lake Zurich	LZ	3.3	135	1976–2010	420	0–40	271 (14)	1976–2009	408	0–135	33	1977–2008	367	0–135	365	Anneville et al., 2004, 2005; Pomati et al., 2012, 2015, 2017
Sempachersee	SE	0.66	87	1984–2014	388	0–15	284 (1)	1982–1998	201	0–87	76	1984–2014	389	0–87	151	Bürgi and Stadelmann, 1991; Monchamp et al., 2018
Hallwilersee	HA	0.285	45	1982–2014	415	0–13	314 (2)	1977–2001	172	0–45	86	1982–2014	414	0–45	124	Bürgi and Stadelmann, 1991; Monchamp et al., 2018
Baldeggersee	BA	0.173	67	1984–2014	403	0–15	295 (0)	1982–1998	205	0–67	87	1984–2014	388	0–67	157	Bürgi and Stadelmann, 1991
Greifensee	GR	0.148	30	1984–2016	429	0–20	401 (20)	1972–2015	519	0–30	96	1975–2015	565	0–30	286	Mieleitner et al., 2008; Monchamp et al., 2018

Lakes are sorted in ascending levels of P-PO4. *Change in sampling location in 1998; ** median of the analyzed time series.

## Materials and Methods

### Data

The plankton dataset consists of microscopic counts of samples collected between 1960 and 2016, mostly in monthly intervals (occasionally biweekly), across 8 Swiss lakes (Table 1). The full raw data and metadata are available from Zenodo (10.5281/zenodo.3582838). Plankton microscopy data from Baldeggersee, Greifensee, Hallwilersee, Sempachersee, and Lake Luzern were collected by Eawag taxonomists, while data from Walensee, upper Lake Zurich (location Lachen) and lower Lake Zurich (location Thalwil) were collected by the Zurich Water Supply Company (WVZ). Plankton samples have consistently been taken in the same locations (with the exception of Lake Luzern in which there was a change in sampling location in 1998, from Kreuztrichter to Obermattbecken) and counted by the same teams of taxonomists over the years, who have also exchanged knowledge and attended the same taxonomy courses. For more details about sampling methods and datasets, see references reported in Table 1.

Samples for phytoplankton microscopy have been collected as integrated samples over the epilimnion [with a Schröder sampler (Mieleitner et al., 2008), where the lower depth varies across lakes; Table 1] or at discrete depths (e.g., Pomati et al., 2012), depending on lake and time period. Taxa abundances were converted to total abundance (cells L^–1^) across the available depths in the epilimnion (when discrete depth samples were collected) to allow comparisons across all lakes (Table 1). Taxonomy of all phytoplankton species in the dataset was harmonized according to modern phytoplankton classification (for examples see Pomati et al., 2012, 2015). Biovolumes for each phytoplankton species were recorded by the taxonomists that counted the samples. Biovolumes represent the median of tens of cells measured for each species over the years. This information has been stored as a meta-database of species biovolumes (H. R. Buergi, unpublished – see online Supplementary Table S1), which was merged with information from the Zurich Water Supply Company database. When species were missing in the Eawag meta-database of species biovolumes, taxa biovolumes were obtained by matching species names against the published database by Kremer et al. (2014) (Supplementary Table S1). From Kremer et al. (2014), we used median taxa biovolumes, which were obtained by collecting data across studies from the literature (Kremer et al., 2014). Biovolume (hereafter size) was expressed in μm^3^ for each counted taxon, and reflects individual cell volumes; for colony forming taxa such as diatoms and cyanobacteria, the biovolume is for individual cells, not the size of colonies. In this study, we linked taxa names (at the species level) with a numeric taxon identifier, which was then used to match each taxon with a corresponding size in the meta-database (Supplementary Table S1). In this way, every taxon in our microscopy data was assigned to a species-specific cell size, with few exceptions of unassigned taxa for which we could not find reliable cell biovolume data (Table 1).

Zooplankton samples were collected as a net tow from the lake bottom to the surface; over time and across locations and lakes (with different maximum depths) the depth span of the net sampling varied. Zooplankton densities were therefore normalized across the database by expressing them as individuals m^–2^ of surface area. In this study we considered only two functional groups of grazers: the unselective filter feeding daphnids and the selective feeding copepods (with calanoids being current feeders and cyclopoids ambush feeders). Consistent information on ciliates and rotifers was not available. Specifically, we focused on the concentration of individuals (juveniles included, but no eggs, ovaria or ephippia) of the following four families: *Bosminidae* and *Daphniidae* (daphnids), *Diaptomidae* and *Cyclopidae* (copepods). As potential drivers of phytoplankton size spectra, we considered the total abundance of all the above families in each sample, and the ratio between selective (*Diaptomidae* and *Cyclopidae*) and unselective (*Bosminidae* and *Daphniidae*) grazers. We focused on these four families as they represent the dominant zooplankton in Swiss lakes, in terms of biomass, provide a strong top–down constraint upon lake phytoplankton, and represent grazing pressures on different size groups (all from daphnids and large from copepods) (Sommer et al., 1986, 2012; Gaedke et al., 2004).

Chemical and physical water parameters were measured monthly (occasionally biweekly) for all lakes in the same locations in which phytoplankton samples were collected (Table 1). The datasets included measurements over the water column in discrete depths, from surface to bottom, with differences in maximum depth and depth resolution depending on the lake and sampling location (Table 1). Data from Walensee, upper Lake Zurich (location Lachen) and lower Lake Zurich (location Thalwil) were produced by WVZ, while data from the other lakes were obtained from local Swiss Cantonal environmental authorities. In this study we focus on the two main environmental drivers of lake change in the Swiss peri-alpine region, as previously assessed (Anneville et al., 2004, 2005; Pomati et al., 2012; Monchamp et al., 2018): water temperature and free available dissolved phosphorus (P-PO4). As noted previously, other variables such as light, turbulence, and other nutrients (e.g., nitrogen) theoretically play important ecological roles, but we focus on temperature and phosphate because previous studies have shown these variables to be the most significant drivers of ecological change in these lakes (Monchamp et al., 2018, 2019). For example, nitrogen levels have been steady over the past four decades and did not correlate significantly with the changes phytoplankton community structure detected in previous studies in the same lakes (Pomati et al., 2012; Monchamp et al., 2018, 2019). *In situ* physical measurement of temperature and laboratory chemical analyses of P-PO4 were performed using standard methods and are comparable across lakes (Pomati et al., 2012; Monchamp et al., 2018). In many cases however, P-PO4 was below detection limits of the method: in such cases we substituted the actual detection limit of the method for the logical character “below detection limit.” For subsequent statistical analyses we used the mean of temperature and mean of P-PO4 over the water column (i.e., the average based on available depths). Variability of temperature over depths was used as an indicator of water column stability (i.e., high variability over depth = strong stratification and therefore stability), and estimated it as the coefficient of variation (standard deviation divided by the mean value) over the sampled depths (Pomati et al., 2012).

### Data Analyses

The overarching goals of the data analysis were to: (a) calculate the SAR for phytoplankton at each time in each lake and (b) characterize the effects of key environmental drivers on the slope of SARs across the lake database. All statistical analysis, including RFs (see below), were performed in the R programing environment (R-Development-Core-Team, 2018).

### Calculation of Size-Abundance Relationships

To analyze the phytoplankton SAR, we fit a linear model to the raw data of taxa abundances relative to their size (both variables in Log_10_, see Figure 1A) per each sampling date, in each lake (no binning, histogram or distribution model was used). Following the advice of White et al. (2008) and Duncanson et al. (2015) we refrained from binning when estimating SAR exponents, given that our phytoplankton size data are continuous and binning introduces biases and arbitrary decisions (e.g., number and width of bins) in the estimation of the scaling exponent. Our approach to study the SAR was based on calculating local size-density relationships, which plot species concentrations in each water sample relative to the mean species biovolume (Reuman et al., 2008) (in this way we retained the information from all the taxa in the database), and on fitting a linear regression to the data (Figure 1A). The model took this form:

Log(density)10=a+b⋅Log(meantaxonbiovolume)10

where densities were expressed as cells L^–1^, and mean taxon biovolume as μm^3^. We extracted from the generalized least square linear fit the coefficient *b*, hereafter “slope” (Figure 1A). We used this metric to examine how SARs vary across lakes and over time. To reduce uncertainties in estimating the scaling exponent, rather than using a maximum likelihood estimator, we opted for a bootstrapping of the species pool. This allows to account for potential biases in the estimation of *b* due to (i) the linear assumption of the model and (ii) taxonomic inconsistencies in the classification and counting of species in the dataset, which spans across many lakes and decades (Straile et al., 2013; Pomati et al., 2015). The linear fit held significant for all lakes (Supplementary Figure S1) and all dates (data not shown). To account for the potential effect of taxonomic biases, we calculated the slope for each date and lake 999 times, by resampling at each round of analysis only 70% of taxa present in the species pool (jackknife bootstrapping). This allowed us to calculate a median and 95% confidence intervals (CIs) for each estimated slope. To confirm and interpret patterns observed when studying changes in the slope of SARs across lakes and over time, we also divided the proportion of biovolumes for all the taxa in our database into three quartiles (Supplementary Figure S2) and investigated patterns in the total abundance of species composing the first (Q1, the smallest 25% of taxa) and third (Q4, the largest 25% of taxa) quartiles.

### Modeling of Size-Abundance Relationships Based on Environmental Drivers

We used RF, a non-parametric machine learning approach, to test for the relative importance and direction of the effects of hypothesized drivers of SARs (Figures 1B–D). RF are a robust machine learning tool based on an ensemble of regression (or decision) trees featuring bootstrap sampling, random variable selection, and model averaging (Breiman, 2001). When presented with complex environmental datasets, RF avoid constraints inherent in traditional statistical approaches, namely the *a priori* specification of functional forms, interactions, and error distributions. In each regression tree within the “RF,” a randomly selected subset of the data is recursively partitioned based on the most strongly associated predictor. At each node, a random subset of the total number of predictors is considered for partitioning. This bootstrapping of both data and explanatory variables minimizes problems associated with the presence of data outliers or artifacts, and with variable collinearity. The final tree prediction is given by the average value of the data within each branch of the tree. By aggregating predictions across trees in the forest, RF are able to reproduce arbitrarily complex shapes and patterns without *a priori* functional form specification (Breiman, 2001; Thomas et al., 2018). In our study, each forest comprised 999 trees. For RF analyses, we used the following R-packages: *randomForest* (version 4.6-14), *randomForestSRC* (version 2.7.0), and *plotmo* (for interactions plots).

We implemented a RF model for the prediction of estimated median slopes after taxa resampling (see the section “Size-abundance relationship analysis”). Modeling of observed slopes instead of estimated medians, however, did not change the results (see Supplementary Figures S3, S4). To explain variation in the slope of size SARs across lakes and over time, we used the following environmental drivers (see also the section “Data”):

- *Temperature*: average water column temperature (variable name *T_*mean*_)* and its coefficient of variation over the sampled depths as a measure of stability (*T*_*CV*_) (Pomati et al., 2012).
- *Nutrients*: mean P-PO4 levels over the sampled depths (*P-PO4_*mean*_*), and total phytoplankton densities as measures of total available resources (*Phytoplankton*_*total*_). While P-PO4 is the limiting factor for phytoplankton growth in our panel of lakes (Anneville et al., 2004; Pomati et al., 2012; Monchamp et al., 2018), total phytoplankton abundances account for total nutrients (phosphorus and nitrogen) available in the systems. Additionally, high levels of phytoplankton densities anti co-vary with light penetration in the water column, and causing light limitation.
- *Grazing*: total densities of daphnids and copepods (*Zooplankton_*total*_)* as a measure of total grazing pressure, and the ratio between abundances of copepods and daphnids (*Zooplankton*_*selectivity*_) as a proxy for the prevalence of size-selective versus non-size-selective grazers, respectively (Sommer et al., 2001; Gaedke et al., 2004; Stibor et al., 2004; Wollrab and Diehl, 2015).

All environmental variables in the model were used without any transformation, with the exception of phytoplankton and zooplankton densities that were Log_10_ transformed. Missing values (12 in total) were imputed automatically by the *rfsrc* function (package *randomForestSRC*) or using the function *rfImpute* (package *randomForest*): NAs are initially replaced by data column medians, then a proximity matrix from a RF model is used to update the imputation of NAs where the imputed values is the weighted average of the non-missing observations. To account for important differences in the morphometry of lakes (Table 1), we included depth (*Lake Depth*) at the sampling site and lake total water volume (*Lake Volume*) in the model. This allowed us to separate the effects of in-lake environmental conditions from lake characteristics in predicting phytoplankton size spectra slopes. Additionally, to compare the magnitude of effects of in-lake environmental drivers relative to the strength of lake long-term temporal trends and seasonal changes, we included as explanatory variables (i) the time sequence of dates for every lake as a proxy for unaccounted time-varying factors (*Time-trend*) and (ii) the sequence of months in the year (*Seasonality*).

The RF model for the median slope is a function of all variables: *T_*mean*_, T_*CV*_, P-PO4_*mean*_, Phytoplankton_*total*_, Zooplankton_*total*_, Zooplankton_*selectivity*_, Lake Depth, Lake Volume, Time-trend, and Seasonality.* This model explained 52% of the variance in slopes. Including the response variable (slope) as an autoregressive term in the model only slightly increased the variance explained (from 51.8 to 52.4%), without significantly changing the effects of explanatory variables (Supplementary Figures S5, S6), likely due to the inclusion of the temporal trend as a predictor in the model.

The importance of each explanatory variable (e.g., T_*CV*_) in the RF model was assessed by permuting it across all generated trees (the forest), and by quantifying the resulting change in the model’s error rate. More important drivers lead to a greater increase in error when omitted from the model (Breiman, 2001). The partial effect of any explanatory variable on the response (slope) can be quantified by averaging, across the forest, the variable values used in the trees to reach terminal nodes. This property of RF allowed us to examine the functional form of the relationship between environmental drivers and slope values, which may be non-linear. We did not include interaction (multiplicative) terms in the RF model: in linear models, interaction terms might bring value by fixing non-linearity or independence violations between the response and the explanatory variables. RF do not have assumptions about linearity and interactions emerge from predicting the response variable over varying levels of a chosen pair of explanatory variables (Breiman, 2001; Thomas et al., 2018).

## Results

### Environmental Changes

**FIGURE 2:**
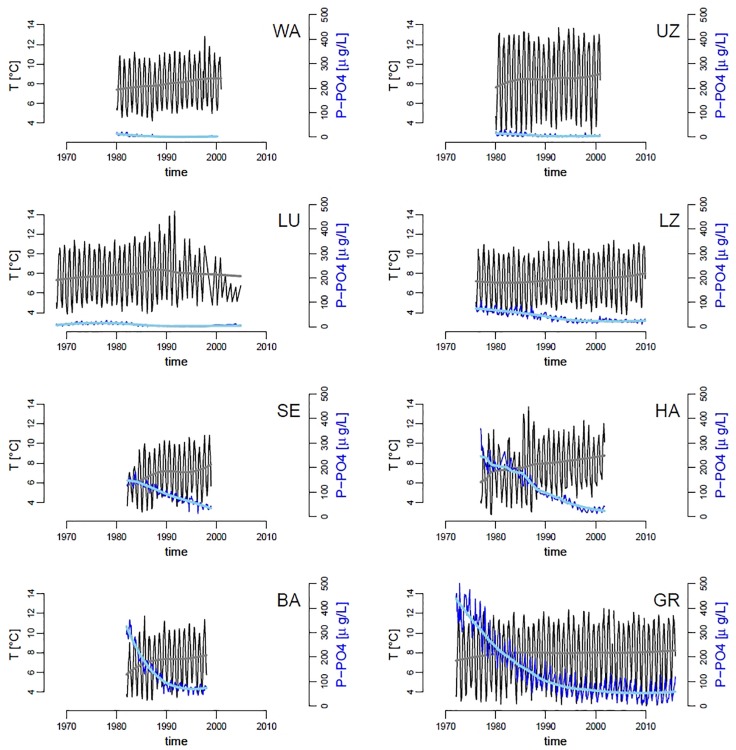
Time series of mean water column temperature (black lines, gray trend;°C) and dissolved inorganic phosphorus (P-PO4, dark blue lines, light blue trend; μg L^–1^), across our panel of lakes. Trend-lines were obtained by locally weighted scatterplot smoothing. Codes in panels represent the name of lakes as in **Table 1**.

Over the past five decades, the mean temperature of the water column has increased in all lakes by an average of 0.85°C (standard error = 0.13), and the mean dissolved phosphorus (P-PO4) concentrations have decreased by an average of 99 μg/L^–1^ (standard error = 38) across all lakes, though the magnitude and pace of change differed clearly by lake (Figure 2). The unusual pattern in Lake Lucerne at the end of the time series is likely due to change in sampling frequency, which has become sporadic and irregular starting from the 1990s (due to complete recovery of the lake from eutrophication, the sampling location was changed and frequency reduced to 2–4 times per year). Along with warming of surface waters, most lakes have shown an increase in stability of the water column (coefficient of variation – CV – of temperature over depths), which is consistent with an increase in thermal stratification (Supplementary Figure S7). P-PO4 levels differ among the lakes (Figure 2), ranging from a high of almost 500 μg L^–1^ in Greifensee to below detection limits (1 μg L^–1^) in Walensee, Upper Lake Zurich and Lower Lake Zurich (Figure 2).

As a consequence of managing P-PO4 discharges and subsequent recovery of lakes from eutrophication, phytoplankton median population densities and total community densities have decreased in all the studied lakes, with few exceptions (namely total algal abundances in Lower Lake Zurich and Baldeggersee, Supplementary Figure S8). Densities of zooplankton (daphnids and copepods), and the ratio between selective (copepods) and non-selective (daphnids) grazers, did not show any strong or general pattern, with most lakes showing no change over time (Supplementary Figure S9). Notable exceptions are Lake Lucerne and Hallwilersee, in which total zooplankton densities have decreased, and WA in which the ratio between copepods and daphnids has increased over time (Supplementary Figure S9). The effects of environmental changes on the median size of taxa in phytoplankton communities were small and inconsistent across lakes (Supplementary Figure S10). In the most oligotrophic lakes (Walensee, Upper Zurich and Lucerne), we observed a slight decrease in median taxa size over time, while the most productive lakes (Sempachersee, Hallwilersee, Baldeggersee and Greifensee) showed a small temporal increase in median size throughout the community (Supplementary Figure S10).

### Dynamics of SAR Slopes

**FIGURE 3:**
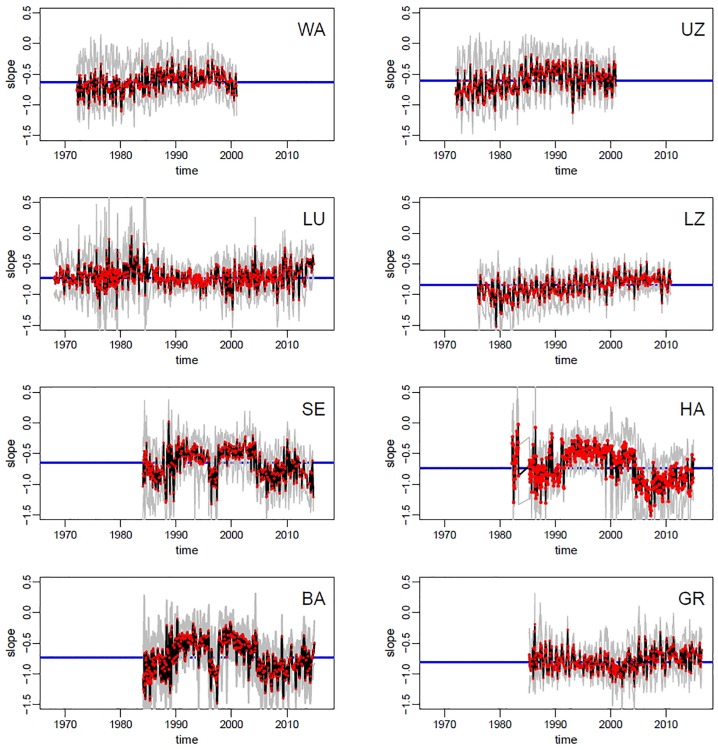
Time series of size spectra slopes across lakes (codes in panels represent lakes as in Table 1). Red dots = observed slopes in the monthly samples; black line = median of bootstrapped slopes (see section Materials and Methods for details); gray lines = 95% confidence intervals of bootstrapping; blue line = median slope of the whole time series.

SAR slopes varied across lakes and over time, as depicted in Figure 3, showing observed and bootstrapped exponents of SARs at each lake-date. The oligotrophic lakes (Walensee, Upper Zurich, Lucerne) had a slightly less negative slope than the most productive lakes (Hallwilersee, Baldeggersee, and Greifensee), but the difference is small (and Baldeggersee and Lake Lucerne have similar slopes). Note the ample variability of slopes within lakes and within years, signaling potential fluctuations in the mechanisms regulating phytoplankton SARs at the seasonal scales. Additionally, for five lakes out of eight (Walensee, Upper Zurich, Lucerne, Lower Zurich, Greifensee), there was a clear increasing long-term temporal trend, with a tendency toward flattening of the slope (i.e., less negative) in the most recent years (Figure 3). For lakes Sempachersee, Hallwilersee and Baldeggersee, the pattern of slopes shows relatively large fluctuations but no clear long-term trend.

**FIGURE 4:**
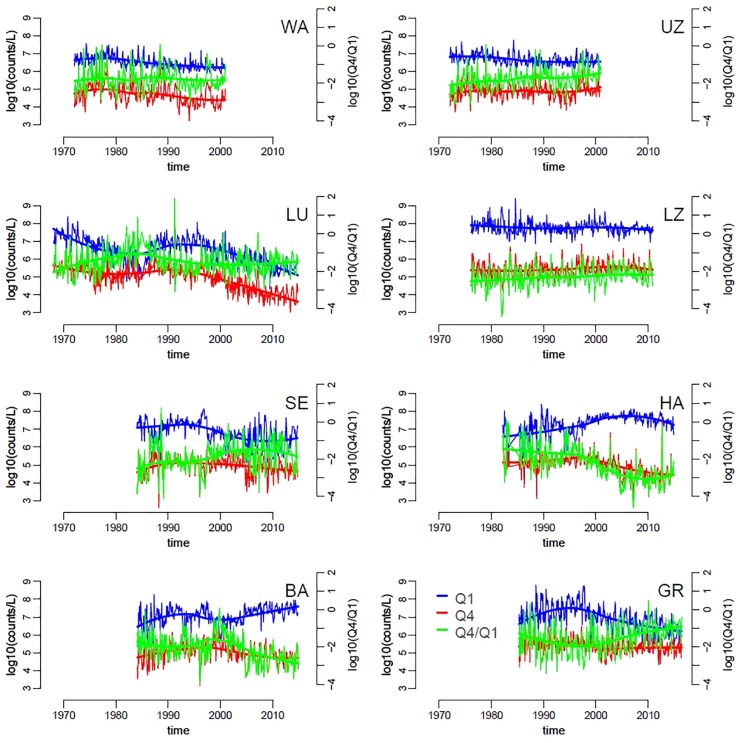
Time series of small and large phytoplankton densities, specifically of the first (Q1, smaller cells, blue) and fourth (Q4, larger cells, red) quartiles of the distribution of species biovolumes, across lakes (codes represent lakes as in **Table 1**). The green lines represent the ratio Q4/Q1. Trend-lines were obtained by locally weighted scatterplot smoothing.

Changes in slopes across lakes and over time corresponded to variation in the absolute and relative abundances of small and large phytoplankton taxa. We focused on the first (Q1) and fourth (Q4) quartiles, respectively, of the distribution of species biovolumes (Supplementary Figure S2). Lakes with steeper slopes (e.g., list lakes here) were characterized by slightly higher density of small taxa (Q1) compared to lakes with flatter slopes (Figure 4). Similarly to patterns in SAR slopes, the abundances of both large and small taxa groups was dynamic within years and over the long term, with differences between lakes. Overall, the average abundance of large taxa seemed to be more stable over time compared to the density of small taxa, which decreased slightly in time for all lakes with the exception of Hallwilersee and Baldeggersee (Figure 4, solid thick lines). An increase in the relative abundances of large versus small taxa (Q4/Q1) was observed in lakes Walensee, Upper Zurich, Lucerne, Lower Zurich and Greifensee (Figure 4), which helps explain the flattening of the slope size spectra in these lakes (Figure 3).

**FIGURE 5:**
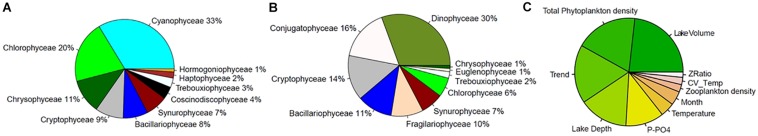
**(A,B)** Relative abundance of phytoplankton classes, expressed as a percentage, in the first (Smaller cells, **A**) and fourth (Larger cells, **B**) quartiles of the distribution of taxa biovolumes. Note that the color palette is consistent across the two charts, and not all groups are present in both size classes (e.g., Cyanophyceae). **(C)** Random forests ranking of predictors of SAR slopes over time and across lakes: the importance reflects the change in mean absolute error of the model when the variable of interest is permuted (the color gradient has no specific meaning, it is only for display).

The composition of small and large groups of phytoplankton in our studied lakes is shown in Figures 5A,B. The small taxa group was dominated by cyanobacteria, green algae, and chrysophytes, while the most prevalent phytoplankton classes in the large taxa group were dinoflagellates, Conjugatophyceae (which includes the desmids), and cryptophytes. Note that the size of taxa in our database corresponds to cell biovolumes, since information in our database about the sizes colonies was not consistent among all lakes and time points (Supplementary Table S1).

### Effects of Environmental Drivers on Slopes

**FIGURE 6:**
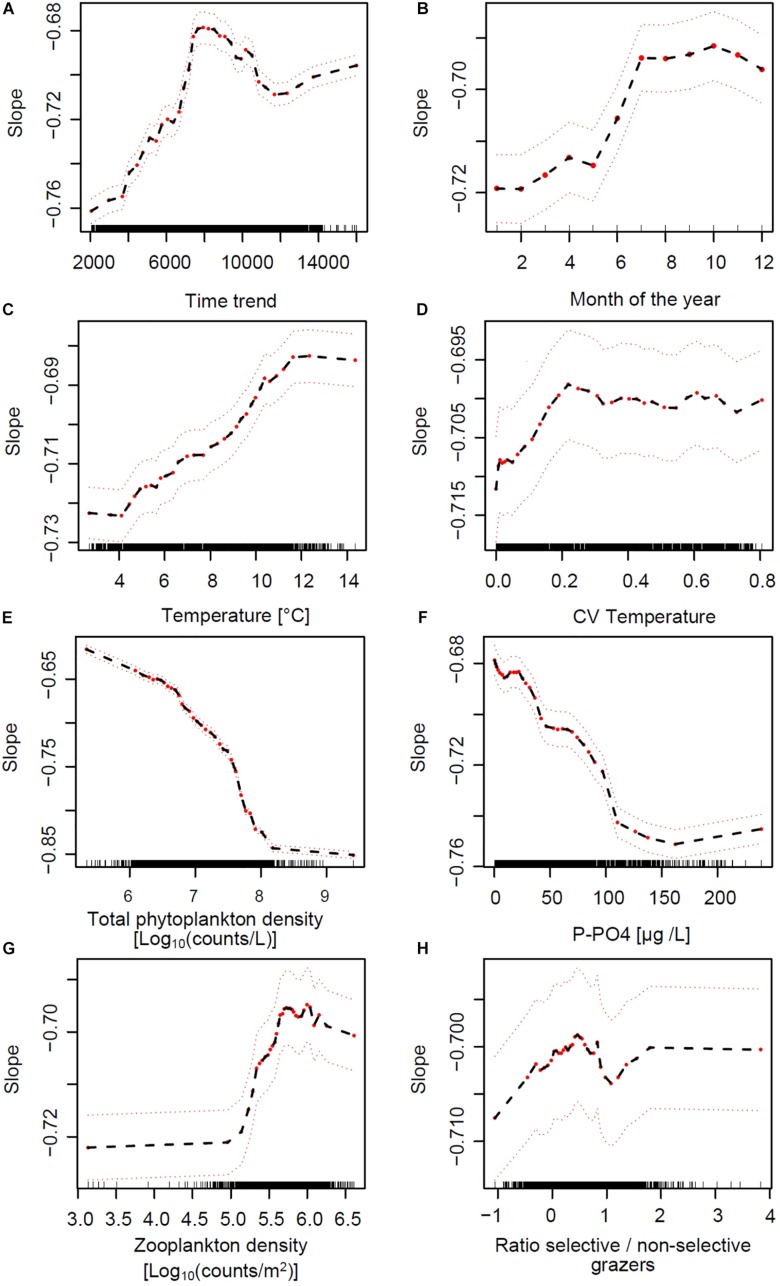
Partial effects of time-varying environmental predictors of the slope of SARs across lakes, based on the RF model (Figure 5C). Red dots represent partial values (the black dashed line follows these partial effects), and dashed red lines indicate a smoothed interval of ± two standard errors. Wile panels **(A,B)** depict the partial effects of time trend and seasonal progression, comparing the direction of effects in panels **(C–H)** of this figure to the hypotheses in **Figures 1B–D** exposes the importance of non-linear dependencies and inconsistencies between theoretical predictions and responses to environmental drivers in the data.

To tease apart the relative effects of environmental drivers on SARs we modeled slope values across lakes and over time using a RF approach (see Section Materials and Methods). The most important explanatory variables predicting the slope of SARs across lakes and over time are those that, when omitted in the RF model, more strongly reduce the performance of the model. These were, respectively: lake volume, total phytoplankton densities, time trend, and lake depth, followed by P-PO4, water temperature, month of the year, total zooplankton density, water column stability (CV of temperature over depths) and zooplankton selectivity (ratio between abundance of copepods and daphnids) (Figure 5C). Based on the analysis of partial effects from the RF model, the time-invariant factors “lake volume” and “depth at sampling site” had a similar consequence on slope: larger and deeper lakes had steeper (more negative) slopes of the SARs (Supplementary Figure S11), with the exception of Greifensee (which is the smallest lake but showed steep slopes). RF-based partial effects of time-varying environmental variables on slope of SAR exposed the importance of non-linear dependencies and inconsistencies between theoretical predictions (Figures 1B–D) and patterns in the data. Time trend, included in the RF model to allow extracting the effects of all unaccounted time-varying factors across lakes, showed a steady increase of slope from values ranging −0.75 toward less negative values during the 1970s and 1980s, with a peak of −0.68 in the early 1990s (Figure 6A). The slope then decreased again in the 2000s and remained in the range of value of −0.70 at present. Extracting seasonal succession from the data using the RF model revealed steeper slopes in winter and spring, and flatter (less negative) slopes in summer and autumn (Figure 6B).

The partial effects of water column thermal structure, nutrient supply and grazing on the slopes of SARs are depicted in Figures 6C–H. Patterns extracted from the RF analysis of partial effects revealed evident non-linear responses of slope to these general environmental drivers. Increasing water column temperature (average over depths) and stability (CV over depths) showed positive and weak effects on slope up to values of 12°C and 0.2, respectively, after which the response curve appeared to saturate (Figures 6C,D). Total phytoplankton cells density (a measure of total productivity of the system) and dissolved inorganic phosphorus (the main growth limiting factor) showed a negative effect on slope, saturating on the low end at 8 Log_10_(counts L^–1^) and 100 μg L^–1^, respectively (Figures 6E,F). The effects of nutrients on changes in SAR slope were stronger compared to those of temperature (see the scale of the *Y*-axes in Figures 6C,D compared to Figures 6E,F). A key finding of the RF analysis is that the patterns in Figures 6C–F are the opposite of what expected from theory and depicted in Figures 1B,C as hypotheses. Total zooplankton grazing had the expected positive effect on slope, starting from densities around 5 and saturating at 6 Log_10_(counts m^–2^) (Figure 6G). The effect of the ratio between selective and non-selective grazers on slope was very weak and potentially positive in its direction (Figure 6H).

**FIGURE 7:**
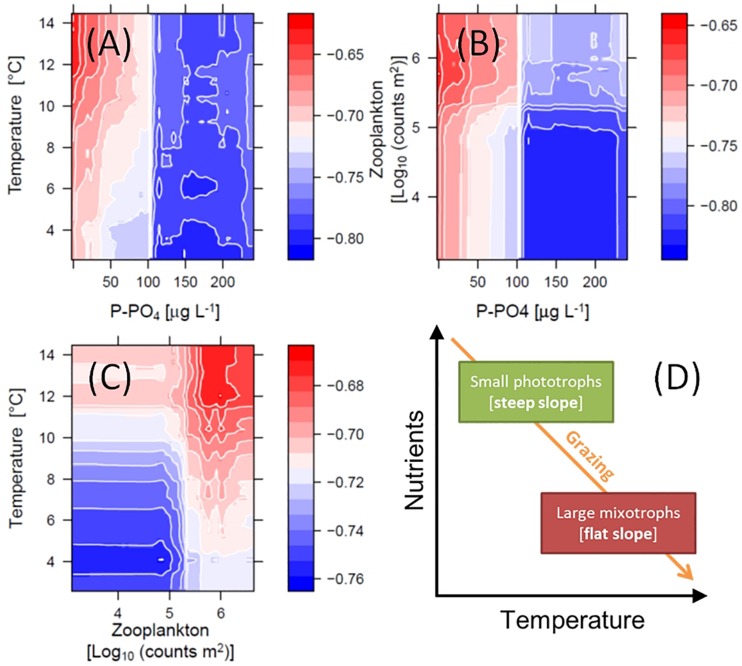
Interacting effects of environmental drivers on the slope of SARs. Color-coded contour plots in **(A–C)** depict the RF model inferred interactions, which emerge from predicting slope (*Z*-axis) over varying levels of the chosen pair of explanatory variables (while holding others at their medians): temperature and dissolved phosphorus **(A)**, total zooplankton densities and dissolved phosphorus **(B)**, and temperature and total zooplankton densities **(C)**. **(D)** Conceptualized interaction of temperature, resource availability and grazing effects on phytoplankton community composition and slope of SARs.

The non-linear patterns in Figure 6, emerged from the RF analysis, suggest possible multiplicative effects (e.g., interactions) and threshold responses of SAR slope to in-lake environmental drivers. The RF model predicted effects of temperature, PO4 and total grazing on slope, for example, changed direction at defined levels (Figures 6C,F,G). These potential interactions are illustrated by color-coded contour plots generated by the RF model, showing the jointed predicted effects of the main ecological drivers on slope (Figure 7). P-PO4 levels and total zooplankton densities show evident thresholds that influence both their direct effects and the effects of co-varying drivers (Figures 7A–C). The negative effects of increasing temperature on slope were stronger under nutrient limitation and low zooplankton densities (see deep blue shades in Figures 7A,C), and the positive effects of nutrients and of grazing were stronger under high temperature (see bright red shades in Figures 7A,C). Specifically, high temperature and high total zooplankton grazing synergized with low P-PO4 to predict the least negative (flattest) values of slope (bright red shades in Figure 7). The steepest slopes (deep blue shades) are instead predicted for low temperature, low zooplankton levels, and P-PO4 values between 110 and 200 μg L^–1^ (Figure 7). Note the abrupt change in predicted response (slope) crossing the value of 100 μg L^–1^ of P-PO4 (Figures 6F, 7A,B), and 5.2 Log_10_ (counts m^–2^) of total zooplankton density (Figures 6G, 7B,C). The interactive effects of water temperature and total zooplankton grazing showed low slope values (bright red) for temperature comprised between 12 and 14°C and zooplankton densities between 5.5 and 6, and high slope values (deep blue) at 4°C and low zooplankton densities (Figure 7C). Note that low temperature and high phosphorus are always associated with steep slopes (deep blue color), while high temperature and low phosphorus correspond to flat slopes (bright red color, Figure 7).

## Discussion

Random forests analysis allowed us to model and explain the observed variation in the slope of size SARs across eight lakes and over decades of time (Figure 3), based on abiotic and biotic environmental drivers (Figure 2 and Supplementary Figures S7–S9). As mentioned in Section “Materials and Methods,” this machine learning approach is indifferent to outliers, not biased by *a priori* specification of response functions (e.g., linearity), and allows to extract robust patterns from noisy and high-dimensional datasets (Thomas et al., 2018). The most striking pattern emerging from our data analysis was the high prevalence of small phytoplankton taxa in more nutrient rich environments, signaled by steeper slopes of SARs under high nutrient levels. This pattern is the opposite of our theoretical expectation (Figure 1C) and is in contrast with what has been observed previously in nutrient rich freshwater and marine environments (Cavender-Bares et al., 2001; Gaedke et al., 2004; Marañón, 2015; Guiet et al., 2016; Sprules et al., 2016). It is, however, the predominant pattern in the data, consistent across lakes and over time (Figures 2–4), and emerged unequivocally from the RF analysis of partial effects of environmental drivers (Figures 6, 7). While deeper and larger ecosystems tend to be characterized by a more oligotrophic environment and higher dominance of small phytoplankton taxa, as expected (Marañón, 2015) (Table 1 and Supplementary Figure S11), eutrophic lakes in our dataset have steeper slopes (Figure 3). The pattern in our data is mostly driven by changes in abundance of small taxa, which decrease over time (Figure 4). Over the temporal span of this study, lakes have undergone a process of re-oligotrophication (Figure 2) (Anneville et al., 2004; Monchamp et al., 2018). Concomitantly, we detected a decrease in the slope of phytoplankton SARs toward less negative values (i.e., a reduction of small phytoplankton forms over time) (Figures 3, 4). This long-term trend in re-oligotrophication is likely the strongest component of the effect of nutrient changes on SARs: the decadal trend in nutrient levels covers a much larger range than the seasonal fluctuations (Figures 2, 5). This pattern of temporal decrease in slope values emerged in the RF analysis as partial effect of the time trend, showing a minimum of the SAR slope in the early 1990s, which is when most lakes stabilized their decreasing trend in phosphorus levels (Figure 2). This happened alongside with warming, causing stronger and more stable stratification, which reinforced the oligotrophication process in the upper water column where phytoplankton thrive (Anneville et al., 2004; Pomati et al., 2012; Posch et al., 2012; Yankova et al., 2017; Lepori et al., 2018).

The most representative taxonomic classes belonging to the small phytoplankton group in our dataset are the cyanobacteria, followed by the green algae (Figure 5A). Both of these classes have unicellular and colonial forms, with cyanobacteria being predominantly colonial while green algae are largely unicellular (Reynolds, 2006). They all appear in our database as small phytoplankton because our compiled information includes only cell biovolumes: colony size was not available for all lakes and all dates (see Section Materials and Methods). We acknowledge that the use of cell biovolume as a proxy for size, with no consistent information about colony dimensions, might have biased our results. Particularly, cyanobacterial diversity and abundance have dramatically changed over the studied period across the chosen lakes, due to interacting oligotrophication and climate change. Temporal trends in taxonomic alpha and beta diversity are consistent at the regional scale and have favored in increase in richness and prevalence of colony forming cyanobacteria (Monchamp et al., 2018, 2019). It is plausible to hypothesize that changes in diversity and abundance of the Cyanobacteria might have biased the data analysis toward an increasing importance of small sized taxa, due to the strong dynamics of this (primarily colonial) phytoplankton group over the past decades. We therefore tested for this bias by excluding the entire class Cyanobacteria from the data. We then estimated slopes and confidence intervals for each lake and date by resampling the species pool without cyanobacteria, and modeled the median of slope distributions using the same RF approach reported in Methods and Results for the full dataset. The RF model of cyanobacteria-free slopes showed slightly different relative importance of explanatory variables, however the directions of partial effects for environmental drivers matched very closely those reported in Figure 6 and Supplementary Figure S12. The trends we document therefore do not result from changes in cyanobacteria only, advocating against strong biases in the analyses due to a lack of information about phytoplankton colony size.

The above test suggests that the pattern of decreasing abundance of small phytoplankton (flattening of the slope of SARs) over declining nutrient levels (Figures 2–4, 6, 7) is robust. Together with cyanobacteria, eukaryotic algae have declined under oligotrophication, reinforced by climate warming, as previously noted (Yankova et al., 2017; Lepori et al., 2018). Decreasing nutrient levels appeared to penalize smaller taxa, which are mostly phototrophic, more strongly than larger forms, which in our data are predominantly mixotrophic (Figures 5, 7D) (Reynolds, 2006). This is consistent with previous empirical and theoretical evidence suggesting that large mixotrophic species can survive and thrive in nutrient depleted conditions by engaging in heterotrophy and phagotrophy (Andersen et al., 2015; Ward and Follows, 2016), while small phototrophs have higher growth rates when carbon and inorganic nutrients are abundant (Edwards et al., 2012). It has been recently noted that the relative importance of mixotrophic algae in lakes increases as nutrients decrease (Waibel et al., 2019). Being large and mixotrophic appeared in our study to be an advantageous strategy in lakes undergoing oligotrophication and climate warming (Yankova et al., 2017; Lepori et al., 2018).

In addition to nutrient uptake rates and resource uptake strategies, phytoplankton SARs are also influenced by susceptibility to general and selective zooplankton grazing, which might co-vary with environmental conditions (Sommer et al., 2001; Stibor et al., 2004; Barton et al., 2013; Marañón, 2015). The impact of total zooplankton on the slope of phytoplankton SARs matched the general expectations emerging from the literature (compare Figure 1D with Figures 6G,H). The ratio between selective and non-selective grazers (copepods/daphnids) had a negligible effect on size distributions, likely due to a coarse grouping of juvenile and adult forms of calanoids (current feeders) and cyclopoids (ambush feeders), which might have very different size-specific effects on phytoplankton. This could have biased the RF analysis by adding noise to this variable, and therefore reducing its predictive power. Our proxy for zooplankton size-selectivity, the ratio between copepods and daphnids, is also affected by the lack of data on a very important group of size-selective grazers: ciliates and rotifers. Albeit not being dominant in lakes in terms of biomass, they are very significant drivers of changes in phytoplankton community structure (Stibor et al., 2004; Sommer et al., 2012; Wollrab and Diehl, 2015). On the other hand, total zooplankton abundance had a clear positive effect on slope (Figure 6G). This result was consistent with previous evidence of crustacean abundance having a positive consequence on the slope of phytoplankton size spectra in Lake Müggelsee (Gaedke et al., 2004). Our data highlight a previously unnoticed non-linear (saturating) shape of this effect. According to our hypotheses (outlined in the Introduction), grazing pressure should also interact with the effects of resource availability on the slope of phytoplankton SARs, and the outcomes of our data analysis confirmed this prediction – however, with the opposite direction. Specifically, a combination of high grazing pressure and low (instead of high) nutrient levels robustly favored large phytoplankton (Figures 7B,D). A positive interaction between zooplankton grazing and warming was also detected in the data (Figure 7C), indicating the prevalence of large phytoplankton under high grazing pressure and high temperature conditions (Figure 7D). This pattern is supported by findings that consumption by herbivores (grazing rates) increases more strongly with temperature than primary production (Rose and Caron, 2007), strengthening the top–down control from grazers on phytoplankton abundance and community structure under warming conditions (Winder and Sommer, 2012; Cloern, 2018).

The above consideration brings us the second most striking pattern in our data, which is the positive effect of water temperature on the slope of phytoplankton SARs: large phytoplankton taxa are more prevalent in warmer environments. Given the monthly frequency of our sampled community data, we note that the effects of temperature are necessarily linked to changes at the monthly, seasonal, and inter-annual scale. The pattern was in fact evident from partial effects of temperature and stability (Figures 6C,D) that resembled the seasonal progression (from winter to summer – Figure 6B) and the temporal trend (climate warming – Figure 6A): they all drove slopes toward less negative values (Figures 6A–D). While the effect of stability of the water column was weak (Figures 5C, 6D), water temperature had a clearly positive relationship with slope (Figure 6C). The direct effect of water warming on plankton community SAR, predicted to be negative (Figure 1B) and mediated by increase in metabolic rates, has been previously detected under laboratory controlled conditions, and after short-term warming of experimental mesocosms (Atkinson et al., 2003; Yvon-Durocher et al., 2011). It has been also noted, however, that the direct effects of temperature are small and may be hard to estimate in natural phytoplankton communities (Marañón et al., 2012; Mousing et al., 2014) and, when detectable, might be minor compared to the co-varying or interacting effects of seasonality and nutrient levels (Marañón et al., 2015, 2018). Surveys (Mousing et al., 2014), theoretical modeling (Sentis et al., 2017), and long-term experimental studies (Yvon-Durocher et al., 2015), suggest that the strongest effect of warming in aquatic communities is mediated by indirect effects of temperature through changes in grazing rates (as noted above), and resource availability (due to suppressed vertical mixing) (Winder and Sommer, 2012). The former might actually have the strongest effect of favoring large phytoplankton due to increasing metabolic rates of grazers and heavier grazing pressure under warming conditions (Yvon-Durocher et al., 2015; Cloern, 2018). Our data analysis supports this previous evidence and suggests a conceptual model of the detected patterns in which phytoplankton size distributions respond to interacting temperature, resource availability, and grazing pressure by favoring small phototrophic algae under high levels of nutrients and low temperature and grazing, and large mixotrophs in oligotrophic conditions when temperature and grazing are high (Figure 7D). This outlined concept matches the predictions of the PEG model of phytoplankton seasonal succession for spring and summer phytoplankton communities, respectively (Sommer et al., 1986). Slopes of phytoplankton SARs and community composition toward the end of lake time series, in fact, resemble summer assemblages, supporting previous reports of a temporal progression of lake ecosystems toward a “summer-like” environment and phytoplankton community structure (Anneville et al., 2004; Posch et al., 2012; Pomati et al., 2017; Yankova et al., 2017; Monchamp et al., 2018).

## Conclusion

In our analysis, each lake had a different baseline biomass distribution among phytoplankton size classes, likely because of different food-web architectures. Our data indicate that co-occurring seasonal and long-term environmental changes significantly control these structures. We highlight a three way interaction between effects of warming, nutrient supply, and grazing that might depend on seasonality and on the long-term history of the analyzed ecosystems, in this case lakes experiencing climate warming and oligotrophication. Regardless of the fact that cyanobacteria have increased in prevalence within and between lakes, and occurrences of cyanobacterial blooms have been increasingly reported, our data analysis suggests that they are not the only group contributing to the observed long-term changes in the phytoplankton community SARs. While cyanobacterial fluctuations contributed a significant proportion of the variation in the abundance of small sized phototrophs over time, the increase in importance of large mixotrophic species in recent monitoring data requires further investigations. Some recent reports corroborate our findings (Waibel et al., 2019), however more evidence is required to confirm a generalized increase in prevalence of mixotrophs relative to smaller phototrophs along oligotrophication and warming gradients. Our results suggest changes in plankton trophic interactions over the course of the past half century, with potentially fundamental consequences for the functioning of lake food-webs.

The main results of our analyses contrast with the starting hypotheses based on previous reports, however we are not the first authors to report inconsistencies between theoretical expectations of environmental effects on phytoplankton size distributions and observed patterns (Cermeño et al., 2006; Marañón, 2015; Marañón et al., 2018). Our observations are well supported by basic lake plankton ecology, and we speculate that the inconsistencies between expected and detected effects of environmental drivers are due to four main reasons. First, the sampling frequency of our dataset (monthly) restricts the detection of effects to seasonal and inter-annual scales, while the direct effects of temperature on metabolic rates and the effects of nutrient supply might have the strongest influence on plankton dynamics and the daily and weekly scales (Thomas et al., 2018). Second, previous studies did not specifically attempt to address non-linearities and interactions in co-occurring ecological mechanisms, and this might have confounded the estimation of importance and direction of environmental effects. Third, the data used in this study describe lakes that were not at stationary state: strong effects of time-varying factors like climate warming and re-oligotrophication had profound but potentially transient effects on these ecosystems. The patterns that we detected, therefore, might not be generalizable to stationary state ecosystems. Fourth, since the majority of previous studies come from the marine literature, our results might suggest that there are fundamental differences in how freshwater and marine phytoplankton communities respond to bottom–up and top–down controls. Specifically, we note that grazing by small herbivores such as ciliates and rotifers, which control small phytoplankton under high nutrient supply and were not counted in our datasets, might be weaker in freshwater compared to marine planktonic environments. This is currently an untested hypothesis and could explain the dominance of small algae under eutrophic or high resource conditions.

## Data Availability Statement

The raw data supporting the conclusions of this article are made available by the authors, without undue reservation, through Zenodo (10.5281/zenodo.3582838).

## Author Contributions

CT and FP prepared the datasets. FP designed the study and carried out the data analyses with feedbacks from AB, JS, and KA. FP drafted the manuscript. All authors contributed to the manuscript development and revisions, and approved the final manuscript for publication.

## Conflict of Interest

The authors declare that the research was conducted in the absence of any commercial or financial relationships that could be construed as a potential conflict of interest.

## Supplementary Material

FIGURE S1 Scaling of phytoplankton abundances (Log_10_ cells L^−1^) with size (Log_10_ taxa biovolumes) in each lake dataset.

FIGURE S2 Distribution of phytoplankton biovolumes across the whole dataset (all lakes and all dates): blue lines depict the division of the distribution applied to obtain Q1 (leftmost data, first quartile) and Q4 (rightmost data, fourth quartile) used in Figure 4.

FIGURE S3 Ranking of predictors for the RF model of observed slopes (as opposed to the bootstrapped slopes as in Figure 5C).

FIGURE S4 Partial effects of environmental predictors based on the RF model of observed slopes (as opposed to the bootstrapped slopes as in Figure 6).

FIGURE S5 Ranking of predictors for the RF model of bootstrapped slopes, adding slope at time-lag 1 (previous month data) as a predictor (lag_slope).

FIGURE S6 Partial effects of environmental predictors based on the RF model of bootstrapped slopes, adding slope at time-lag 1 (previous month data) as a predictor (lag_slope).

FIGURE S7 Time series of mean water column temperature (black lines, gray trend) and temperature coefficient of variation (CV, blue line, light blue trend) over the water column (i.e., meant temperature/standard deviation). Codes in panels represent the name of lakes as in Table 1.

FIGURE S8 Time series of total phytoplankton abundances (black lines, gray trend) and median taxa abundances (green lines, gray trend). Codes in panels represent the name of lakes as in Table 1.

FIGURE S9 Time series of total zooplankton abundances (black lines, gray trend) and selectivity (ratio between copepods / daphnids – blue lines, light blue trend). Codes in panels represent the name of lakes as in Table 1.

FIGURE S10 Time series of median phytoplankton size (i.e. biovolume – black lines, blue trend): codes in panels represent the name of lakes as in Table 1.

FIGURE S11 Partial effects of lake morphometry predictors in the RF model (Figures 5C, 6).

FIGURE S12 Ranking of predictors and partial effects of environmental drivers for the RF model of bootstrapped slopes after exclusion of the class Cyanobacteria from the dataset.

TABLE S1 Phytoplankton meta-database, including code identifier of taxa, full taxonomic classification and taxa biovolumes.

## References

[B1] Andersen K. H. Aksnes D. L. Berge T. Fiksen Ø Visser A. (2015). Modelling emergent trophic strategies in plankton. *J. Plankton Res.* 37 862–868. 10.1093/plankt/fbv054

[B2] Andersen K. H. Berge T. Gonçalves R. J. Hartvig M. Heuschele J. Hylander S. (2016). Characteristic Sizes of Life in the Oceans, from Bacteria to whales. *Annu. Rev. Mar. Sci.* 8 217–241. 10.1146/annurev-marine-122414-034144 26163011

[B3] Anneville O. Gammeter S. Straile D. (2005). Phosphorus decrease and climate variability: mediators of synchrony in phytoplankton changes among European peri-alpine lakes. *Freshw. Biol.* 50 1731–1746. 10.1111/j.1365-2427.2005.01429.x

[B4] Anneville O. Souissi S. Gammeter S. Straile D. Wimereux S. M. D. (2004). Seasonal and inter-annual scales of variability in phytoplankton assemblages: comparison of phytoplankton dynamics in three peri-alpine lakes over a period of 28 years. *Freshw. Biol.* 49 98–115. 10.1046/j.1365-2426.2003.01167.x

[B5] Armstrong R. A. (1994). Grazing limitation and nutrient limitation in marine ecosystems: steady state solutions of an ecosystem model with multiple food chains. *Limnol. Oceanogr.* 39 597–608. 10.4319/lo.1994.39.3.0597

[B6] Atkinson D. Ciotti B. J. Montagnes D. J. S. (2003). Protists decrease in size linearly with temperature: ca. 2.5% C-1. *Proc. R. So. B Biol. Sci.* 270 2605–2611. 10.1098/rspb.2003.2538 14728784 PMC1691543

[B7] Barton A. D. Pershing A. J. Litchman E. Record N. R. Edwards K. F. Finkel Z. V. (2013). The biogeography of marine plankton traits. *Ecol. Lett.* 16 522–534. 10.1111/ele.12063 23360597

[B8] Behrenfeld M. J. Boss E. S. (2013). Resurrecting the Ecological Underpinnings of Ocean Plankton blooms. *Annu. Rev. Mar. Sci.* 6 167–194. 10.1146/annurev-marine-052913-021325 24079309

[B9] Bonachela J. A. Raghib M. Levin S. A. (2011). Dynamic model of flexible phytoplankton nutrient uptake. *Proc. Natl. Acad. Sci. U.S.A.* 108 20633–20638. 10.1073/pnas.1118012108 22143781 PMC3251133

[B10] Breiman L. (2001). Random forests. *Mach. Learn.* 45 5–32.

[B11] Bürgi H. R. Stadelmann P. (1991). Plankton succession in lake sempach, lake hallwil and lake baldegg before and during internal restoration measures. *SIL Proc.* 24 931–936. 10.1080/03680770.1989.11898884

[B12] Cavender-Bares K. K. Rinaldo A. Chisholm S. W. (2001). Microbial size spectra from natural and nutrient enriched ecosystems. *Limnol. Oceanogr.* 46 778–789. 10.4319/lo.2001.46.4.0778

[B13] Cermeño P. Marañón E. Harbour D. Harris R. P. (2006). Invariant scaling of phytoplankton abundance and cell size in contrasting marine environments. *Ecol. Lett.* 9 1210–1215. 10.1111/j.1461-0248.2006.00973.x 17040323

[B14] Cloern J. E. (2018). Why large cells dominate estuarine phytoplankton. *Limnol Oceanogr.* 63 392–409.

[B15] Duncanson L. I. Dubayah R. O. Enquist B. J. (2015). Assessing the general patterns of forest structure: quantifying tree and forest allometric scaling relationships in the United States. *Global Ecol. Biogeogr.* 24 1465–1475. 10.1111/geb.12371

[B16] Edwards K. F. Thomas M. K. Klausmeier C. A. Litchman E. (2012). Allometric scaling and taxonomic variation in nutrient utilization traits and maximum growth rate of phytoplankton. *Limnol. Oceanogr.* 57 554–566. 10.4319/lo.2012.57.2.0554

[B17] Finger D. Wüest A. Bossard P. (2013). Effects of oligotrophication on primary production in peri-alpine lakes. *Water Resour. Res.* 49 4700–4710. 10.1002/wrcr.20355

[B18] Finkel Z. V. Beardall J. Flynn K. J. Quigg A. Rees T. A. V. Raven J. A. (2010). Phytoplankton in a changing world: cell size and elemental stoichiometry. *J. Plankton Res.* 32 119–137. 10.1093/plankt/fbp098

[B19] Gaedke U. Seifried A. Adrian R. (2004). Biomass size spectra and plankton diversity in a shallow eutrophic lake. *Int. Rev. Hydrobiol.* 89 1–20. 10.1002/iroh.200310661

[B20] Guiet J. Poggiale J. C. Maury O. (2016). Modelling the community size-spectrum: recent developments and new directions. *Ecol. Modell.* 337 4–14. 10.1016/j.ecolmodel.2016.05.015

[B21] Hansen P. J. Koefoed P. Winding B. (1997). Zooplankton grazing and growth: scaling within the 2-2,000-micrometer body size range. *Limnol. Oceanogr.* 42 687–704. 10.4319/lo.1997.42.4.0687

[B22] Huete-Ortega M. Rodríguez-Ramos T. López-Sandoval D. Cermeño P. Blanco J. Palomino R. (2014). Distinct patterns in the size-scaling of abundance and metabolism in coastal and open-ocean phytoplankton communities. *Mar. Ecol. Prog. Ser.* 515 61–71. 10.3354/meps11007

[B23] Kremer C. T. Gillette J. P. Rudstam L. G. Brettum P. Ptacnik R. (2014). A compendium of cell and natural unit biovolumes for >1200 freshwater phytoplankton species. *Ecology* 95 2984–2984.

[B24] Lepori F. Roberts J. J. Schmidt T. S. (2018). A paradox of warming in a deep peri-Alpine lake (Lake Lugano. Switzerland and Italy). *Hydrobiologia* 824 215–228. 10.1007/s10750-018-3649-1

[B25] Livingstone D. M. (2003). Impact of secular climate change on the thermal structure of a large temperate central European lake. *Clim. Change* 57 205–225. 10.1023/a:1022119503144

[B26] Marañón E. (2015). Cell size as a key determinant of phytoplankton metabolism and community structure. *Annu. Rev. Mar. Sci.* 7 241–264. 10.1146/annurev-marine-010814-015955 25062405

[B27] Marañón E. Cermeño P. Latasa M. Tadonléké R. D. (2012). Temperature, resources, and phytoplankton size structure in the ocean. *Limnol. Oceanogr.* 57 1266–1278. 10.4319/lo.2012.57.5.1266

[B28] Marañón E. Cermeño P. Latasa M. Tadonléké R. D. (2015). Resource supply alone explains the variability of marine phytoplankton size structure. *Limnol. Oceanogr.* 60 1848–1854. 10.1002/lno.10138

[B29] Marañón E. Lorenzo M. P. Cermeño P. Mouriño-Carballido B. (2018). Nutrient limitation suppresses the temperature dependence of phytoplankton metabolic rates. *ISME J*. 12 1836–1845. 10.1038/s41396-018-0105-1 29695860 PMC6018665

[B30] Mieleitner J. Borsuk M. Bürgi H.-R. Reichert P. (2008). Identifying functional groups of phytoplankton using data from three lakes of different trophic state. *Aquat. Sci.* 70 30–46. 10.1007/s00027-007-0940-z

[B31] Monchamp M.-E. Spaak P. Dubois N. Domaizon I. Bouffard D. Pomati F. (2018). Homogenization of lake cyanobacterial communities over a century of climate change and eutrophication. *Nat. Ecol Evolu.* 2 317–324. 10.1038/s41559-017-0407-0 29230026

[B32] Monchamp M.-E. Spaak P. Pomati F. (2019). High dispersal levels and lake warming are emergent drivers of cyanobacterial community assembly in peri-Alpine lakes. *Sci. Rep.* 9 7366–7366. 10.1038/s41598-019-43814-2 31089175 PMC6517590

[B33] Mousing E. A. Ellegaard M. Richardson K. (2014). Global patterns in phytoplankton community size structure-evidence for a direct temperature effect. *Mar. Ecol Prog. Ser.* 497 25–38. 10.3354/meps10583

[B34] Pomati F. Matthews B. Jokela J. Schildknecht A. Ibelings B. W. (2012). Effects of re-oligotrophication and climate warming on plankton richness and community stability in a deep mesotrophic lake. *Oikos* 121 1317–1327. 10.1111/j.1600-0706.2011.20055.x

[B35] Pomati F. Matthews B. Seehausen O. Ibelings B. W. (2017). Eutrophication and climate warming alter spatial (depth) co-occurrence patterns of lake phytoplankton assemblages. *Hydrobiologia* 787 375–385. 10.1007/s10750-016-2981-6

[B36] Pomati F. Tellenbach C. Matthews B. Venail P. Ibelings B. W. Ptacnik R. (2015). Challenges and prospects for interpreting long-term phytoplankton diversity changes in Lake Zurich (Switzerland). *Freshw. Biol.* 60 1052–1059. 10.1111/fwb.12416

[B37] Posch T. Köster O. Salcher M. M. Pernthaler J. (2012). Harmful filamentous cyanobacteria favoured by reduced water turnover with lake warming. *Nat. Clim. Change* 2 1–5.

[B38] Rasconi S. Gall A. Winter K. Kainz M. J. (2015). Increasing water temperature triggers dominance of small freshwater plankton. *PloS One* 13:e0140449. 10.1371/journal.pone.0140449 26461029 PMC4603799

[B39] R-Development-Core-Team (2018). *R: A Language and Environment for Statistical Computing.* Vienna: R-Development-Core-Team.

[B40] Reuman D. C. Holt R. D. Yvon-Durocher G. (2014). A metabolic perspective on competition and body size reductions with warming. *J. Anim. Ecol.* 83 59–69. 10.1111/1365-2656.12064 23521010

[B41] Reuman D. C. Mulder C. Raffaelli D. Cohen J. E. (2008). Three allometric relations of population density to body mass: theoretical integration and empirical tests in 149 food webs. *Ecol. Lett.* 11 1216–1228. 10.1111/j.1461-0248.2008.01236.x 18803644

[B42] Reynolds C. S. (2006). *Ecology of Phytoplankton.* Cambridge: Cambridge University Press.

[B43] Rodriguez J. Mullin M. M. (1986). Relation between biomass and body weight of plankton in a steady state oceanic ecosystem. *Limnol. Oceanogr.* 31 361–370. 10.4319/lo.1986.31.2.0361

[B44] Rose J. M. Caron D. A. (2007). Does low temperature constrain the growth rates of heterotrophic protists? Evidence and implications for algal blooms in cold waters. *Limnol. Oceanogr.* 52 886–895. 10.4319/lo.2007.52.2.0886

[B45] Sentis A. Binzer A. Boukal D. S. (2017). Temperature-size responses alter food chain persistence across environmental gradients. *Ecol. Lett.* 20 852–862. 10.1111/ele.12779 28544190

[B46] Sheldon R. W. Prakash A. Sutcliffe H. (1972). The size distribution of particles in the ocean. *Limnol. Oceanogr*. 17 327–340. 10.4319/lo.1972.17.3.0327

[B47] Sommer U. Adrian R. De Senerpont Domis L. Elser J. J. Gaedke U. Ibelings B. W. (2012). Beyond the plankton ecology group (PEG) model: mechanisms driving plankton succession. *Ann. Rev. Ecol.Evolu. Syst.* 43 429–448. 10.1146/annurev-ecolsys-110411-160251

[B48] Sommer U. Gliwicz Z. M. Lampert W. I. Duncan A. (1986). The PEG-model of seasonal succession of planktonic events in fresh waters. *Arch. Hydrobiol.* 106 433–471.

[B49] Sommer U. Sommer F. Santer B. Jamieson C. Boersma M. Becker C. (2001). Complementary impact of copepods and cladocerans on phytoplankton. *Eco. Lett.* 4 545–550. 10.1046/j.1461-0248.2001.00263.x

[B50] Sprules W. G. Barth L. E. Giacomini H. (2016). Surfing the biomass size spectrum: some remarks on history, theory, and application. *Can. J. Fishe. Aquat. Sci.* 73 477–495. 10.1139/cjfas-2015-0115

[B51] Sprules W. G. Brandt S. B. Stewart D. J. Munawar M. Jin E. H. Love J. (1991). Biomass size spectrum of the lake michigan pelagic food web. *Can. J. Fish. Aquat. Sci.* 48 105–115. 10.1139/f91-015

[B52] Sprules W. G. Munawar M. (1986). Plankton size spectra in relation to ecosystem productivity. Size, and Perturbation. *Can. J. Fish. Aquat. Sci.* 43 1789–1794. 10.1139/f86-222

[B53] Stibor H. Vadstein O. Diehl S. Gelzleichter A. Hansen T. Hantzsche F. (2004). Copepods act as a switch between alternative trophic cascades in marine pelagic food webs. *Ecol. Lett.* 7 321–328. 10.1111/j.1461-0248.2004.00580.x

[B54] Straile D. Jochimsen M. C. Kümmerlin R. (2013). The use of long-term monitoring data for studies of planktonic diversity: a cautionary tale from two Swiss lakes. *Freshw. Biol.* 58 1292–1301. 10.1111/fwb.12118

[B55] Thomas M. K. Fontana S. Reyes M. Kehoe M. Pomati F. (2018). The predictability of a lake phytoplankton community, over time-scales of hours to years. *Ecol. Lett.* 21 619–628. 10.1111/ele.12927 29527797

[B56] Verdy A. Follows M. Flierl G. (2009). Optimal phytoplankton cell size in an allometric model. *Mar. Ecol. Prog. Ser.* 379 1–12. 10.3354/meps07909

[B57] Waibel A. Peter H. Sommaruga R. (2019). Importance of mixotrophic flagellates during the ice-free season in lakes located along an elevational gradient. *Aquat. Sci.* 81 45–45. 10.1007/s00027-019-0643-2 31057304 PMC6469636

[B58] Ward B. A. Dutkiewicz S. Jahn O. Follows M. J. (2012). A size-structured food-web model for the global ocean. *Limnol. Oceanogr.* 57 1877–1891. 10.4319/lo.2012.57.6.1877

[B59] Ward B. A. Follows M. J. (2016). Marine mixotrophy increases trophic transfer efficiency, mean organism size, and vertical carbon flux. *Proc. Natl. Acad. S.U.A.* 113 2958–2963. 10.1073/pnas.1517118113 26831076 PMC4801304

[B60] White E. P. Enquist B. J. Green J. L. (2008). On estimating the exponent of power-law frequency distributions. *Ecology* 89 905–912. 10.1890/07-1288.1 18481513

[B61] Winder M. Reuter J. E. Schladow S. G. (2009). Lake warming favours small-sized planktonic diatom species. *Proc. R. Soc BBiol. Sci.* 276 427–435. 10.1098/rspb.2008.1200 18812287 PMC2581674

[B62] Winder M. Sommer U. (2012). Phytoplankton response to a changing climate. *Hydrobiologia* 698 5–16. 10.1007/978-94-007-5790-5_2

[B63] Wollrab S. Diehl S. (2015). Bottom-up responses of the lower oceanic food web are sensitive to copepod mortality and feeding behavior. *Limnol. nd Oceanogr.* 60 641–656. 10.1002/lno.10044

[B64] Yankova Y. Neuenschwander S. Köster O. Posch T. (2017). Abrupt stop of deep water turnover with lake warming: drastic consequences for algal primary producers. *Sci. Rep.* 7 13770–13770. 10.1038/s41598-017-13159-9 29062037 PMC5653828

[B65] Yvon-Durocher G. Allen A. P. Cellamare M. Dossena M. Gaston K. J. Leitao M. (2015). Five years of experimental warming increases the biodiversity and productivity of phytoplankton. *PLoS Biol.* 13:e1002324. 10.1371/journal.pbio.1002324 26680314 PMC4682994

[B66] Yvon-Durocher G. Montoya J. M. Trimmer M. Woodward G. (2011). Warming alters the size spectrum and shifts the distribution of biomass in freshwater ecosystems. *Global Change Biol.* 17 1681–1694. 10.1111/j.1365-2486.2010.02321.x

