# Supplementary figures and images for: Interacting Temperature, Nutrients and Zooplankton Grazing Control Phytoplankton Size-Abundance Relationships in Eight Swiss Lakes
Source: Front Microbiol. 2020 Jan 22;10:3155. doi: 10.3389/fmicb.2019.03155 (PMC6987318; doi:10.3389/fmicb.2019.03155)

WA

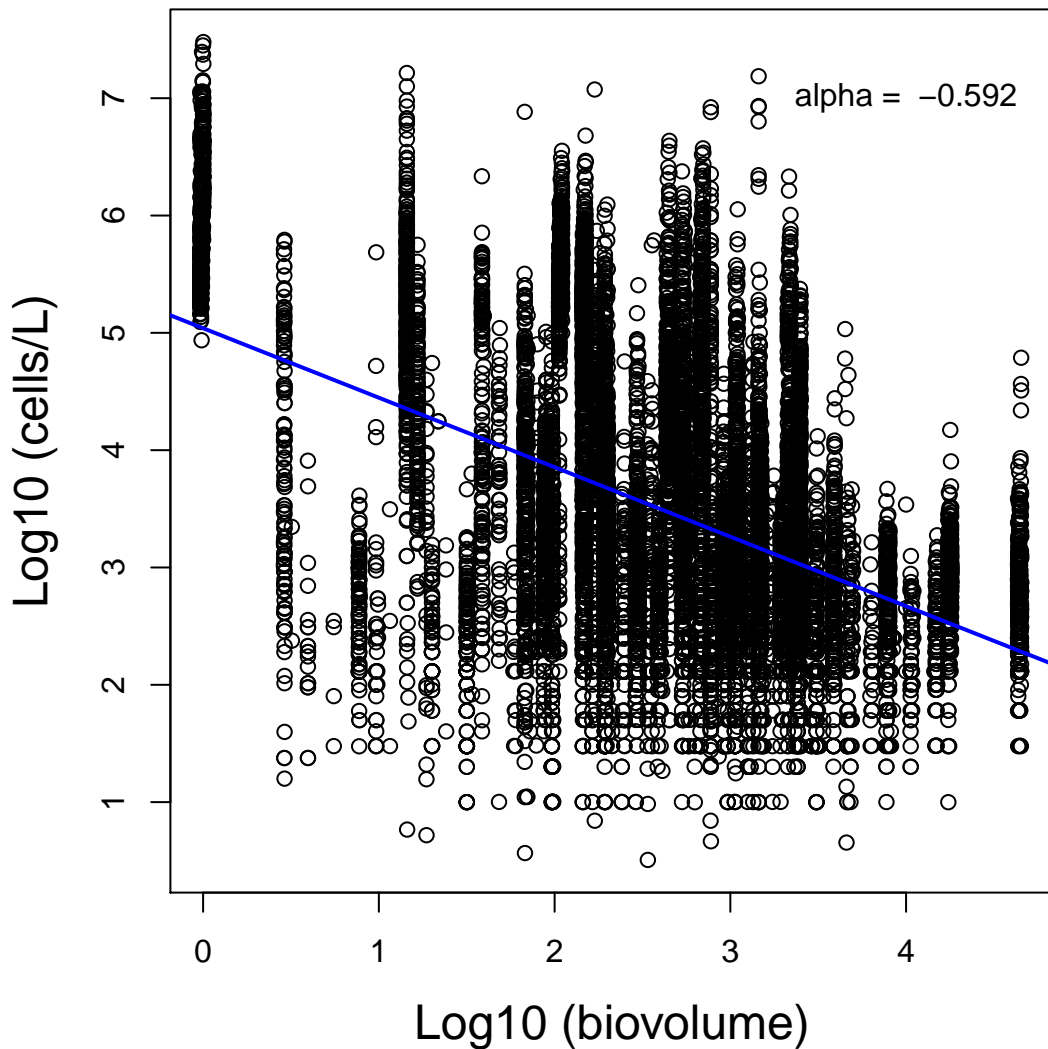

UZ

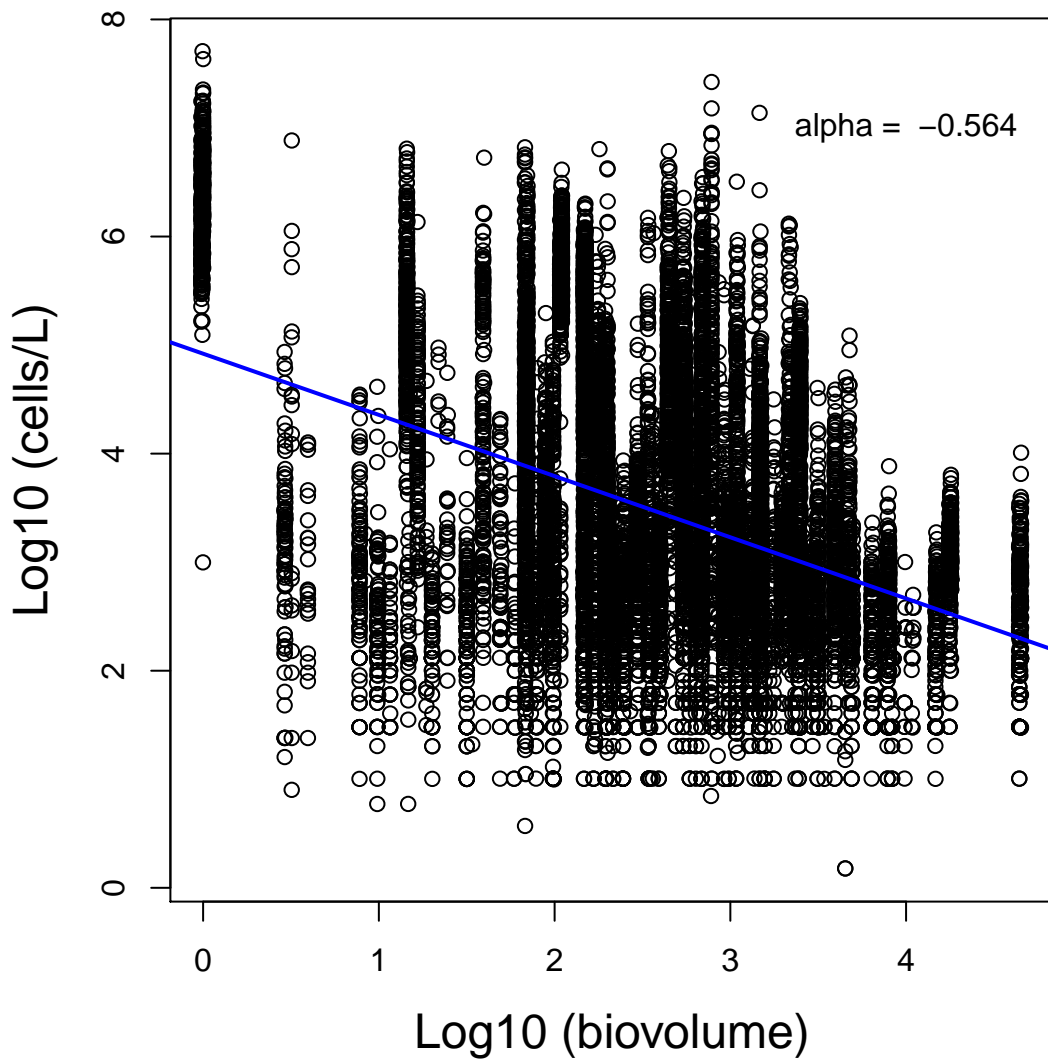

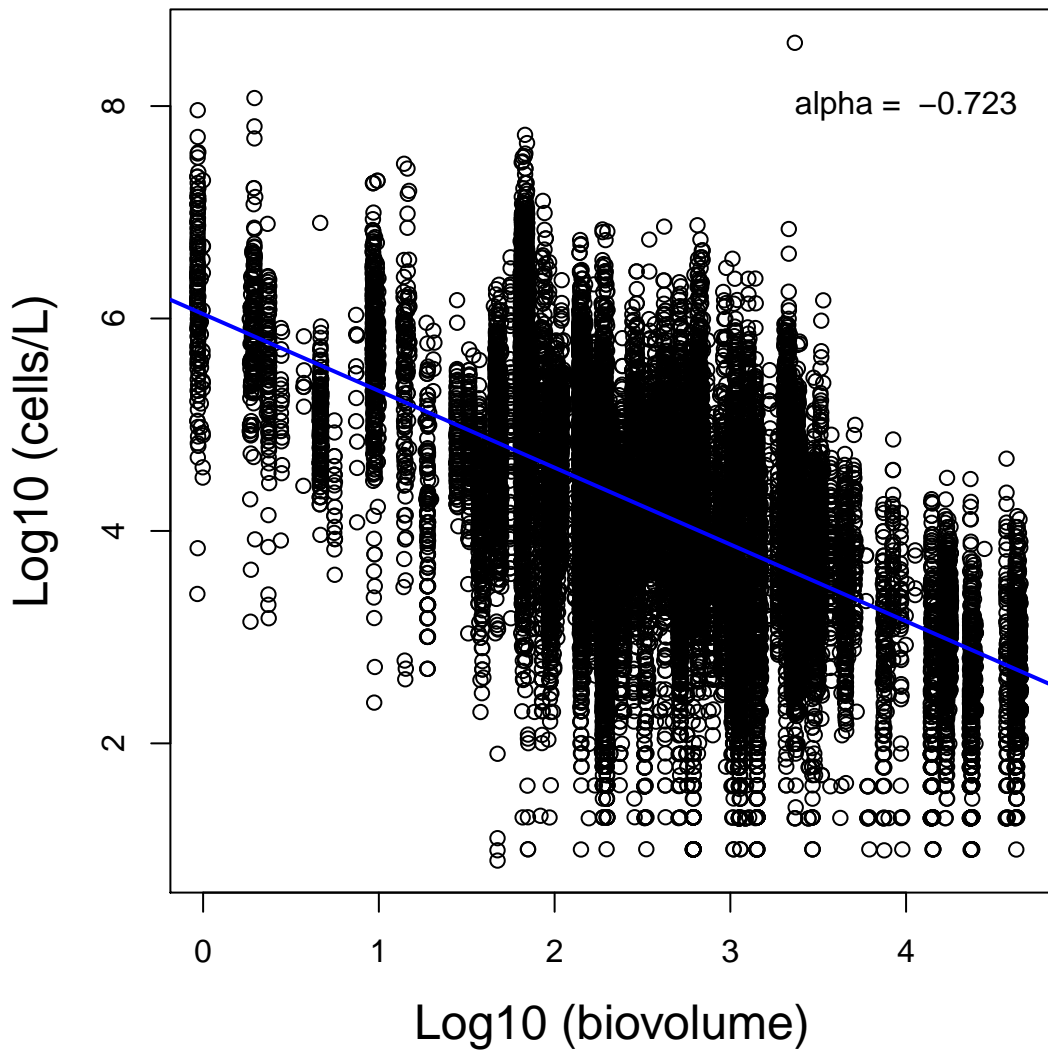

LU

LZ

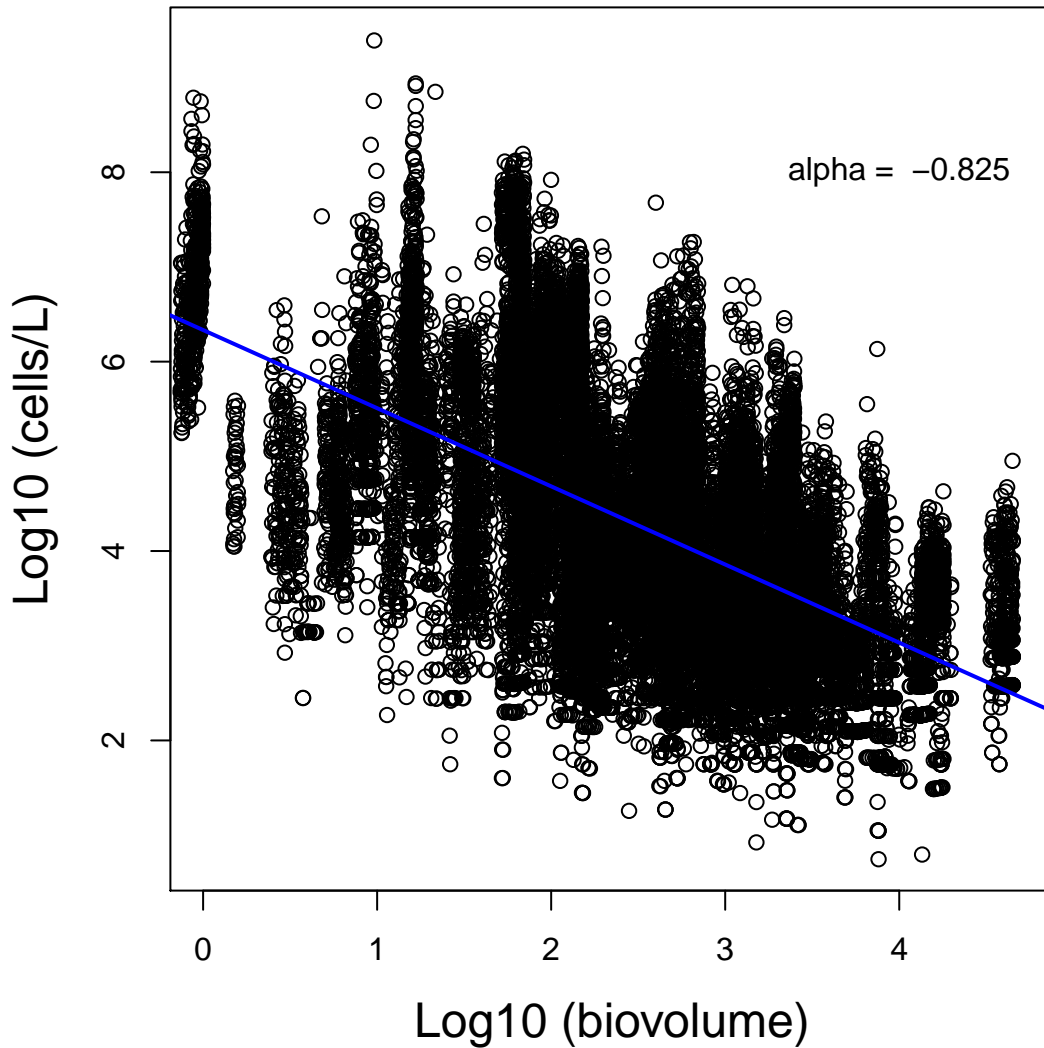

SE

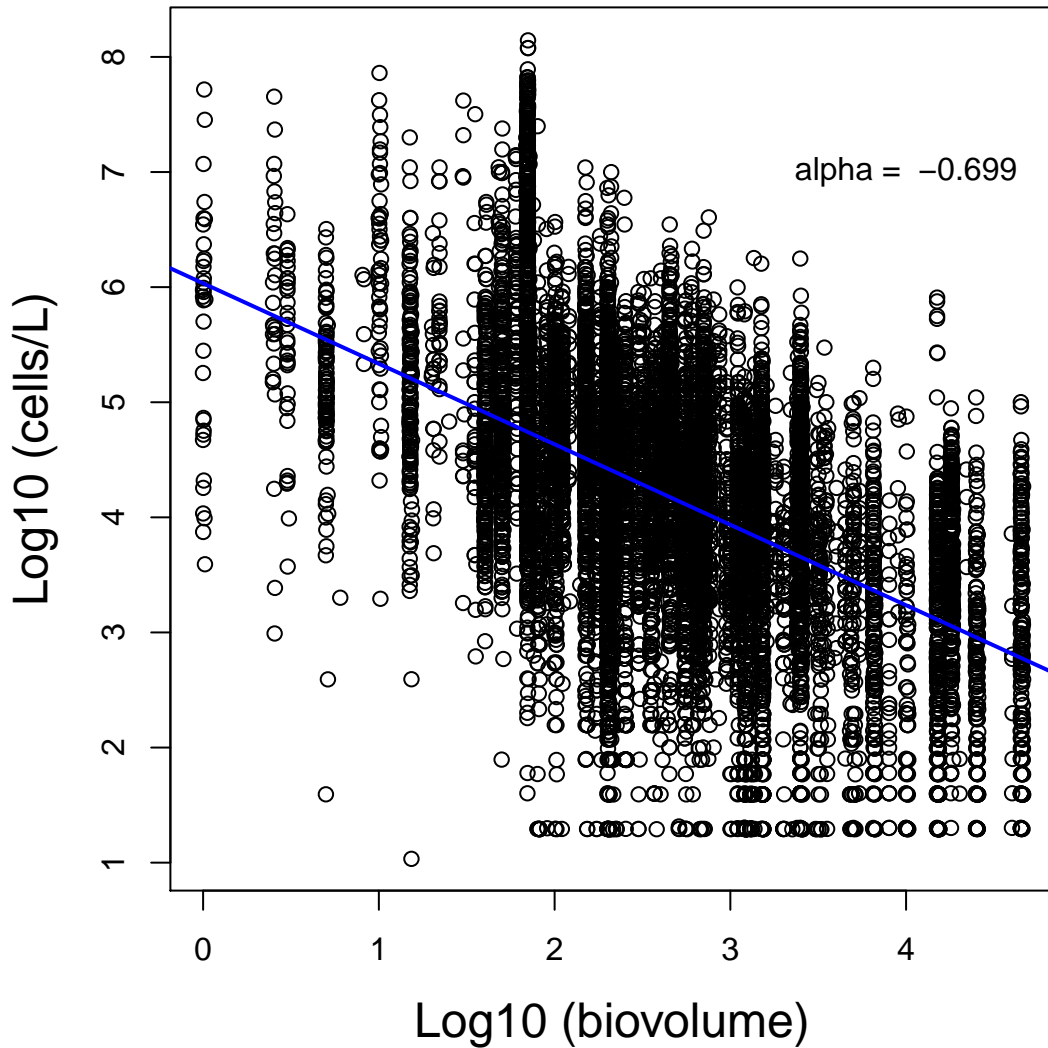

HA

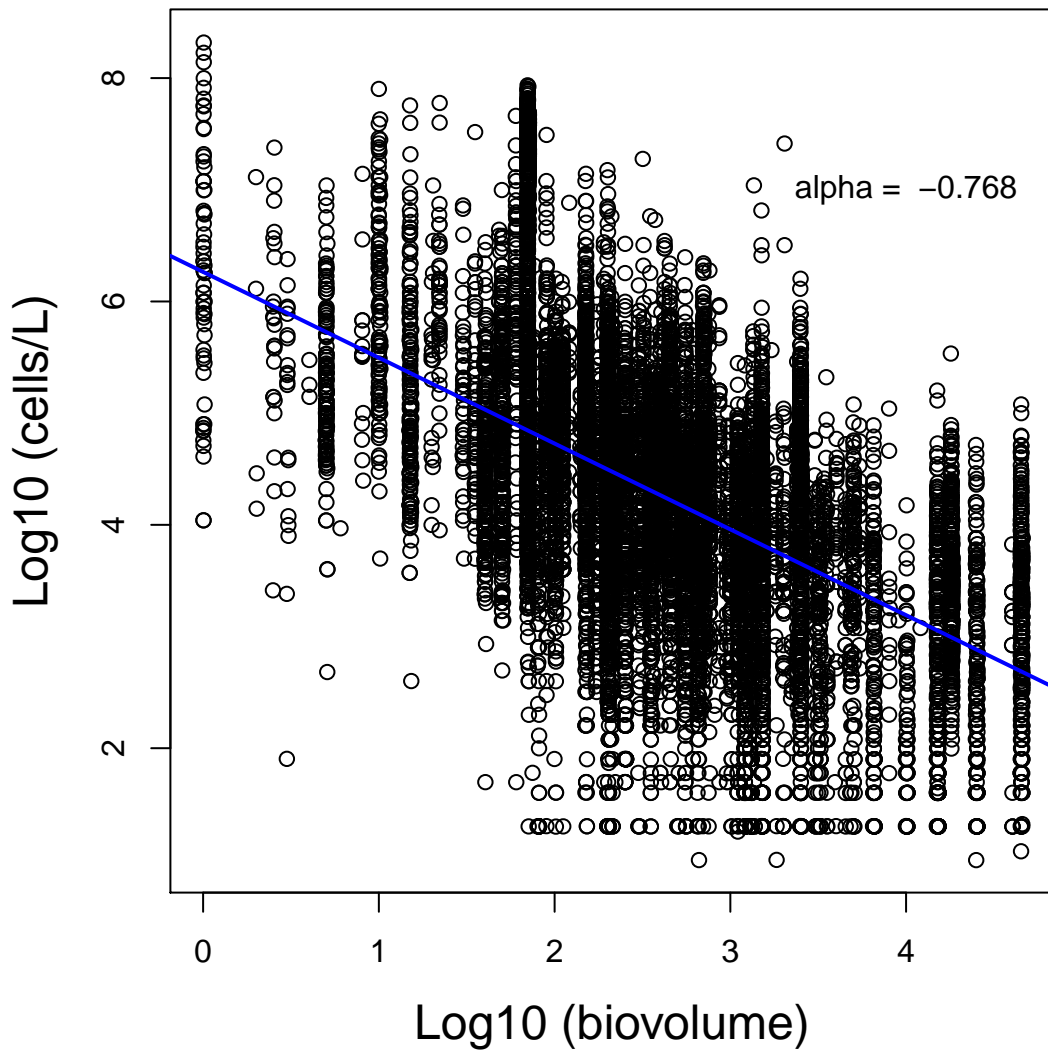

BA

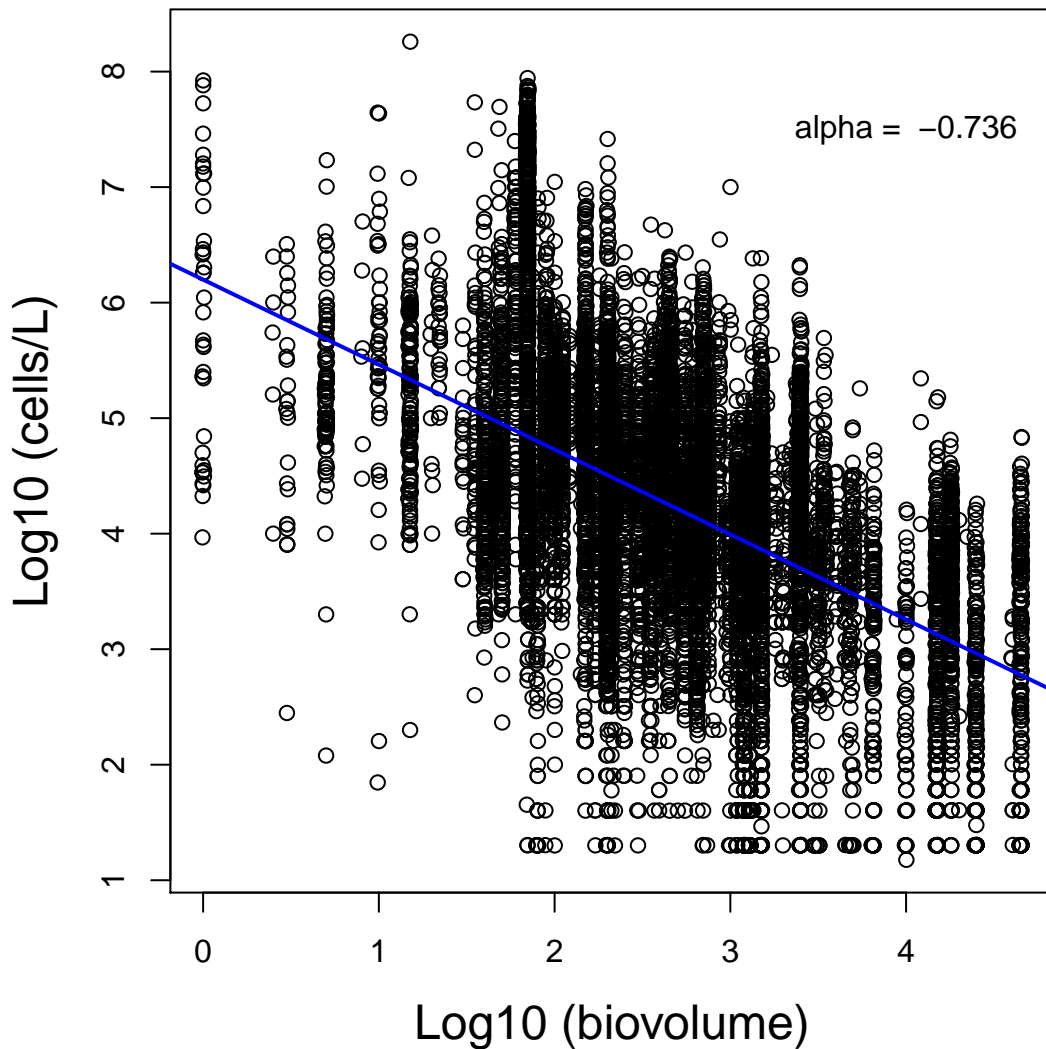

GR

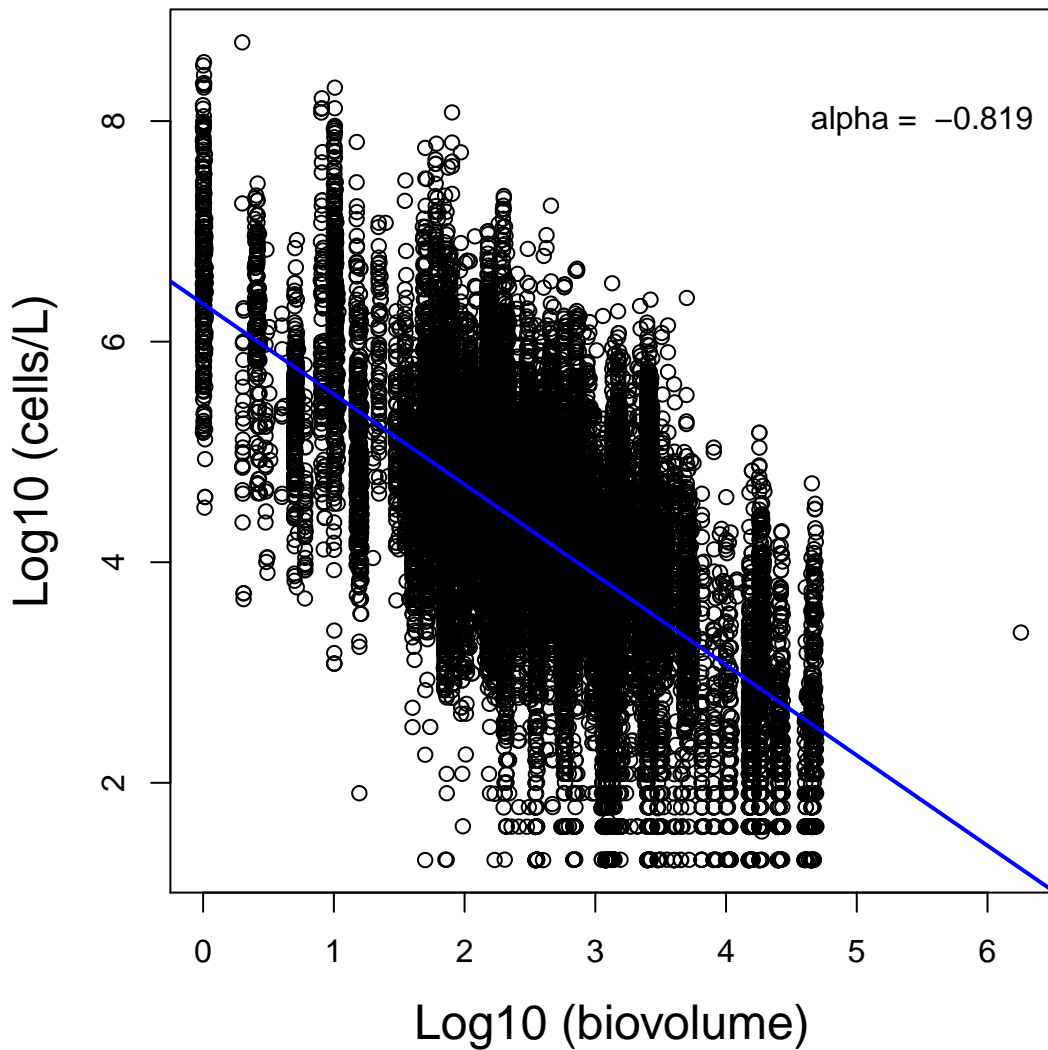

Supplement: FIGURE S1 — Scaling of phytoplankton abundances (Log10 cells L−1) with size (Log10 taxa biovolumes) in each lake dataset. [file Presentation_1.ZIP › FigS1.all_lakes_scaling.pdf]

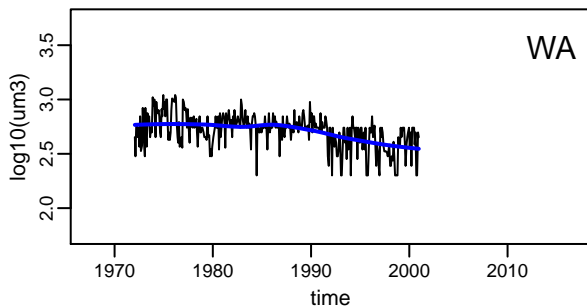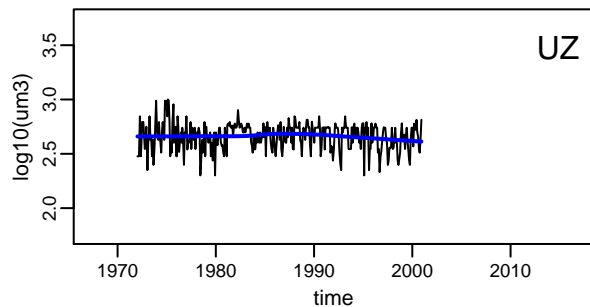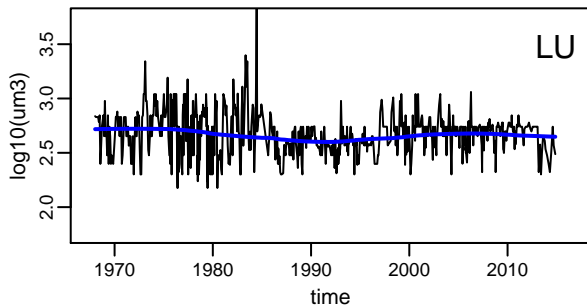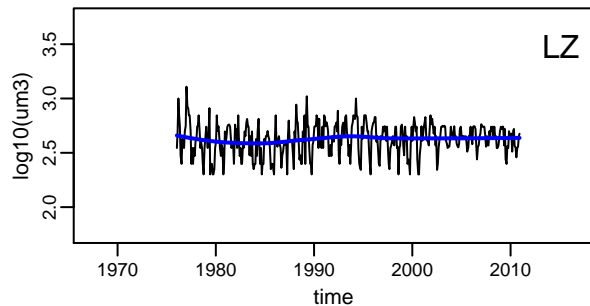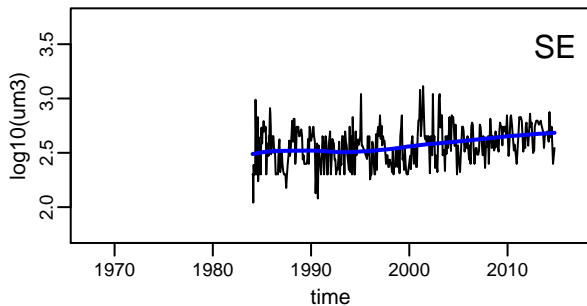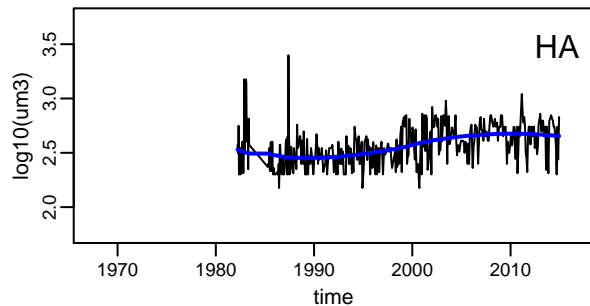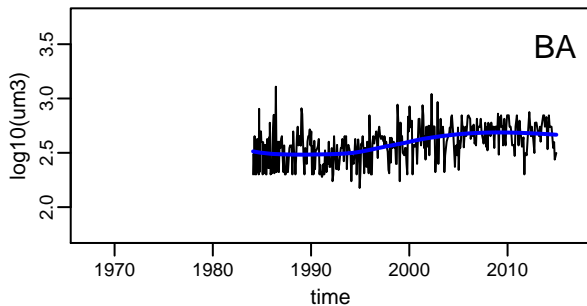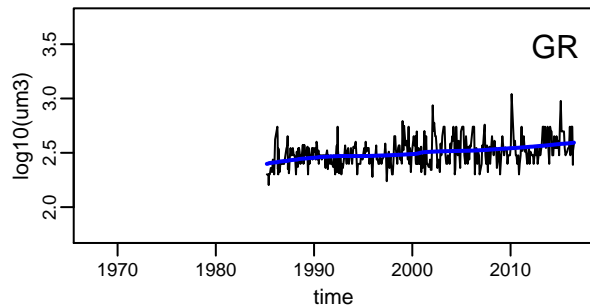

Supplement: FIGURE S1 — Scaling of phytoplankton abundances (Log10 cells L−1) with size (Log10 taxa biovolumes) in each lake dataset. [file Presentation_1.ZIP › FigS10.median_phyto_size.pdf]

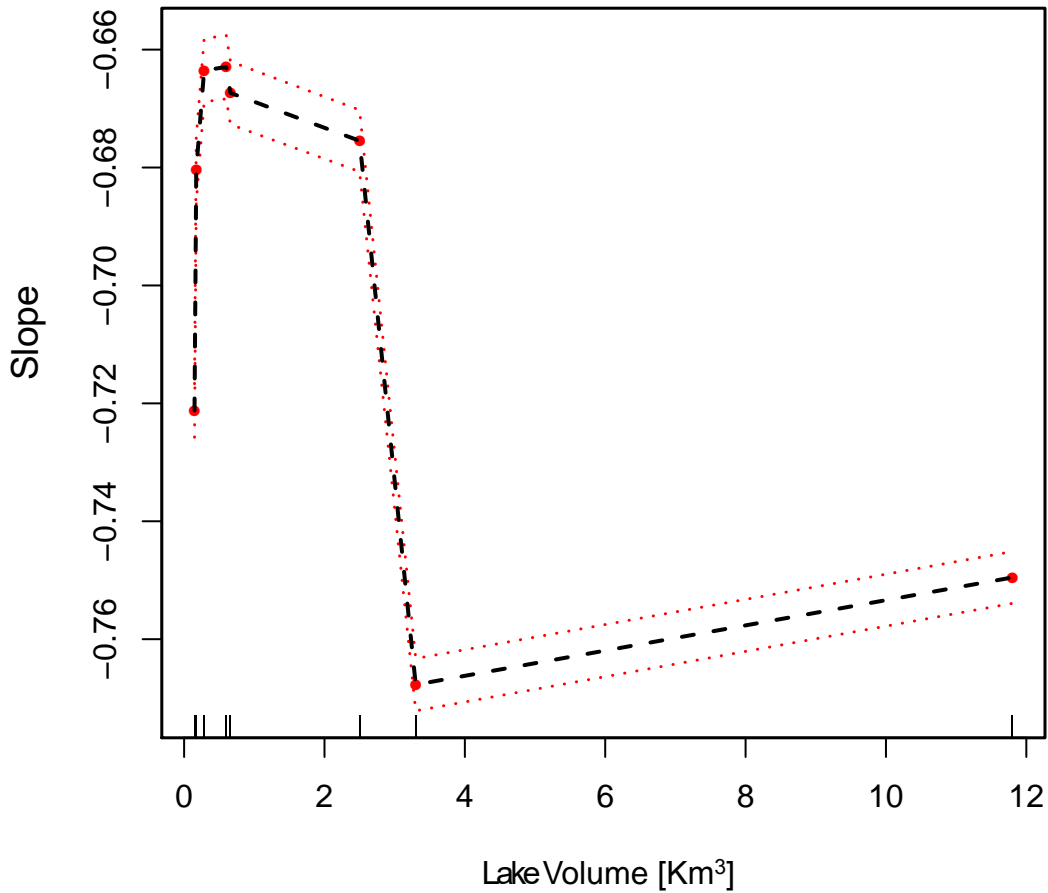

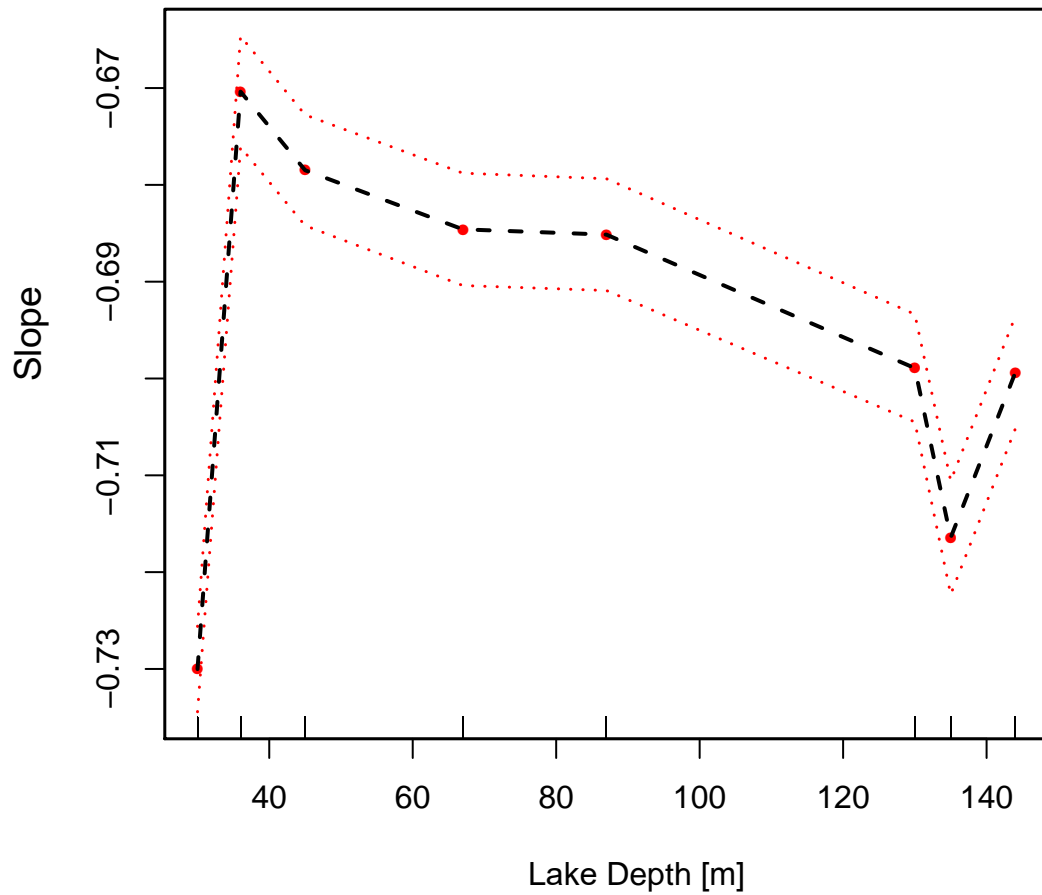

Supplement: FIGURE S1 — Scaling of phytoplankton abundances (Log10 cells L−1) with size (Log10 taxa biovolumes) in each lake dataset. [file Presentation_1.ZIP › FigS11.lake_morphometry_effects_rf.pdf]

**% variance explained: 57.91**

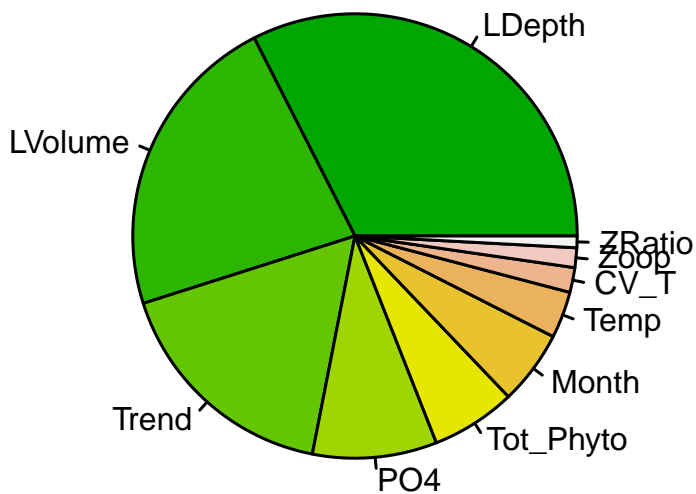

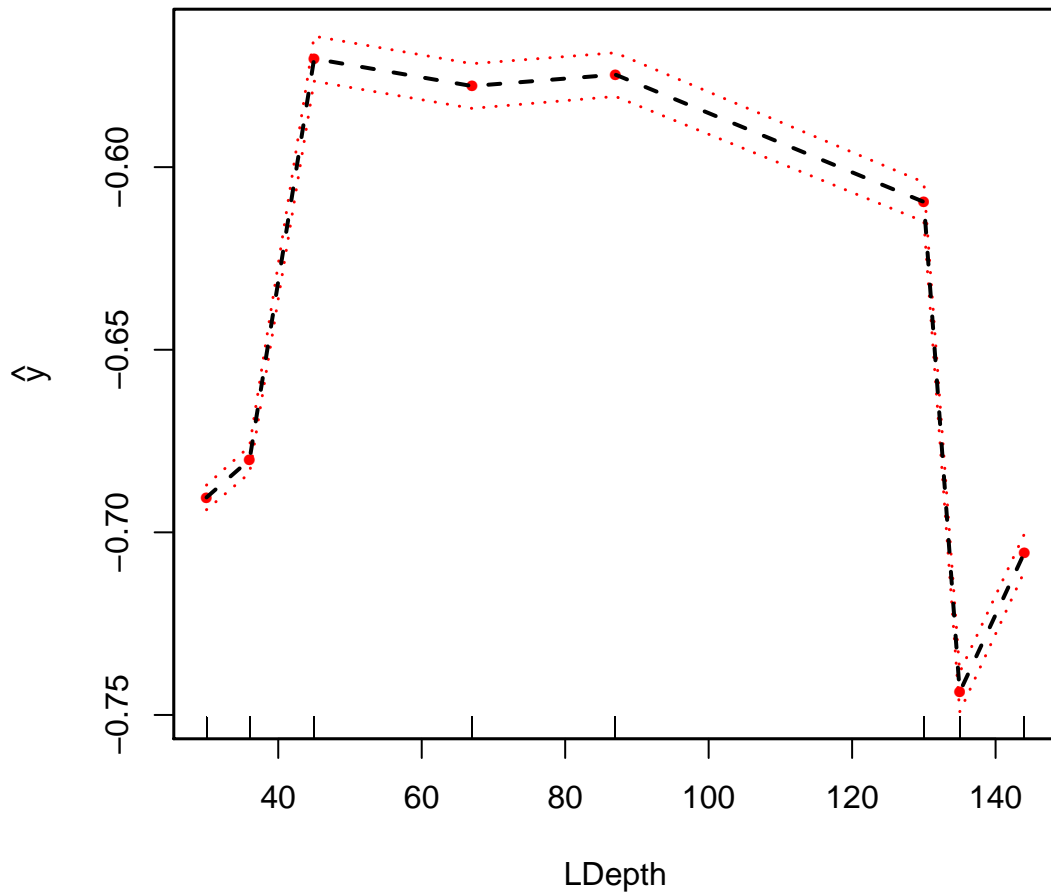

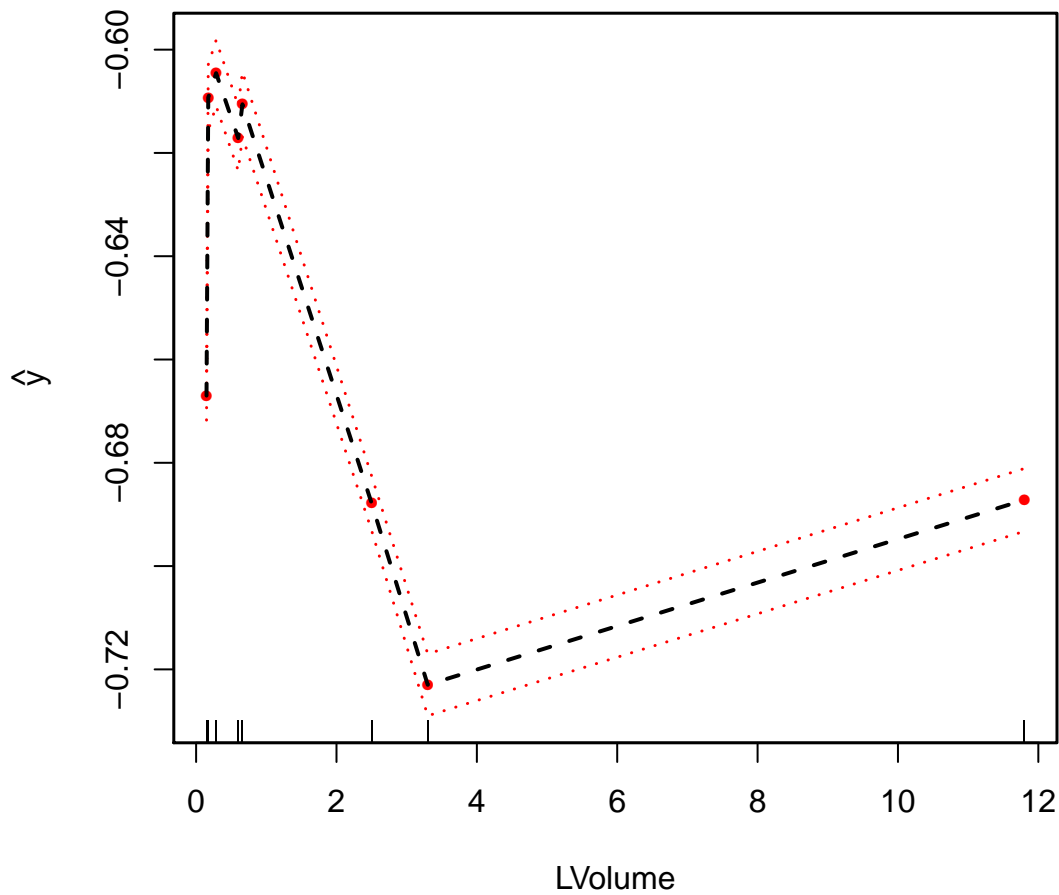

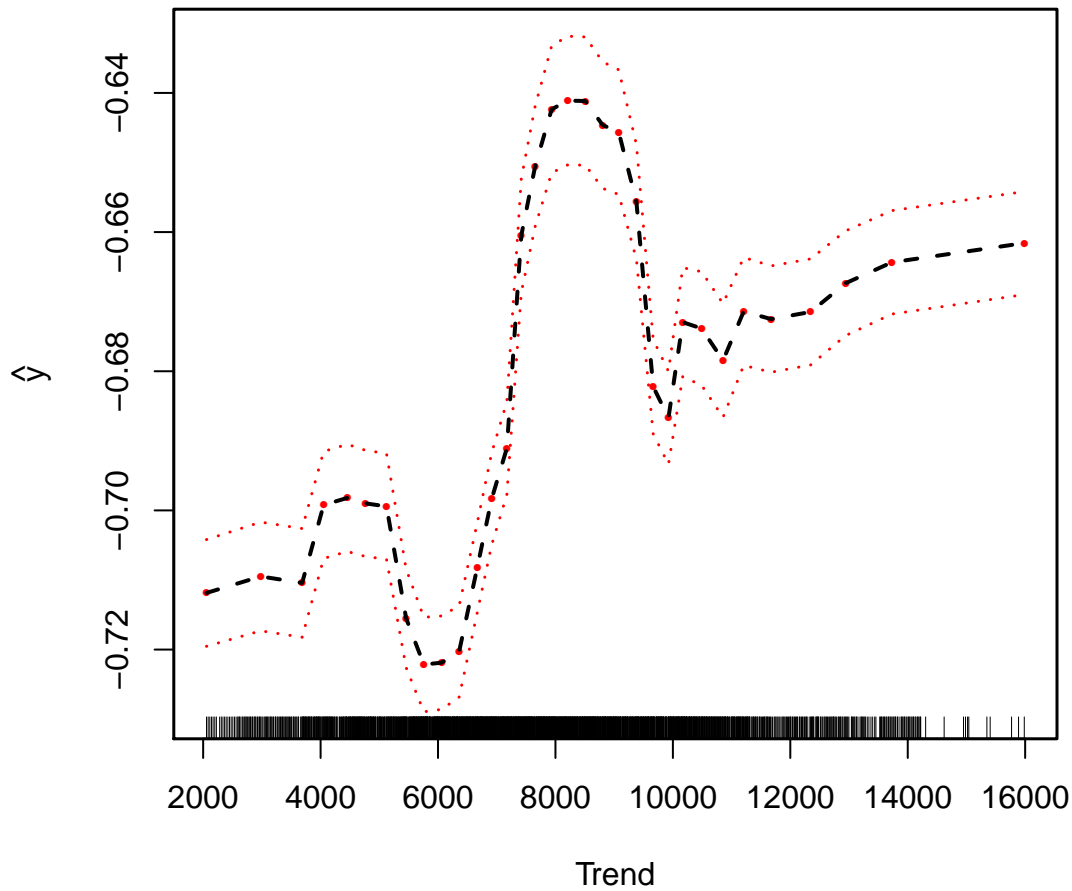

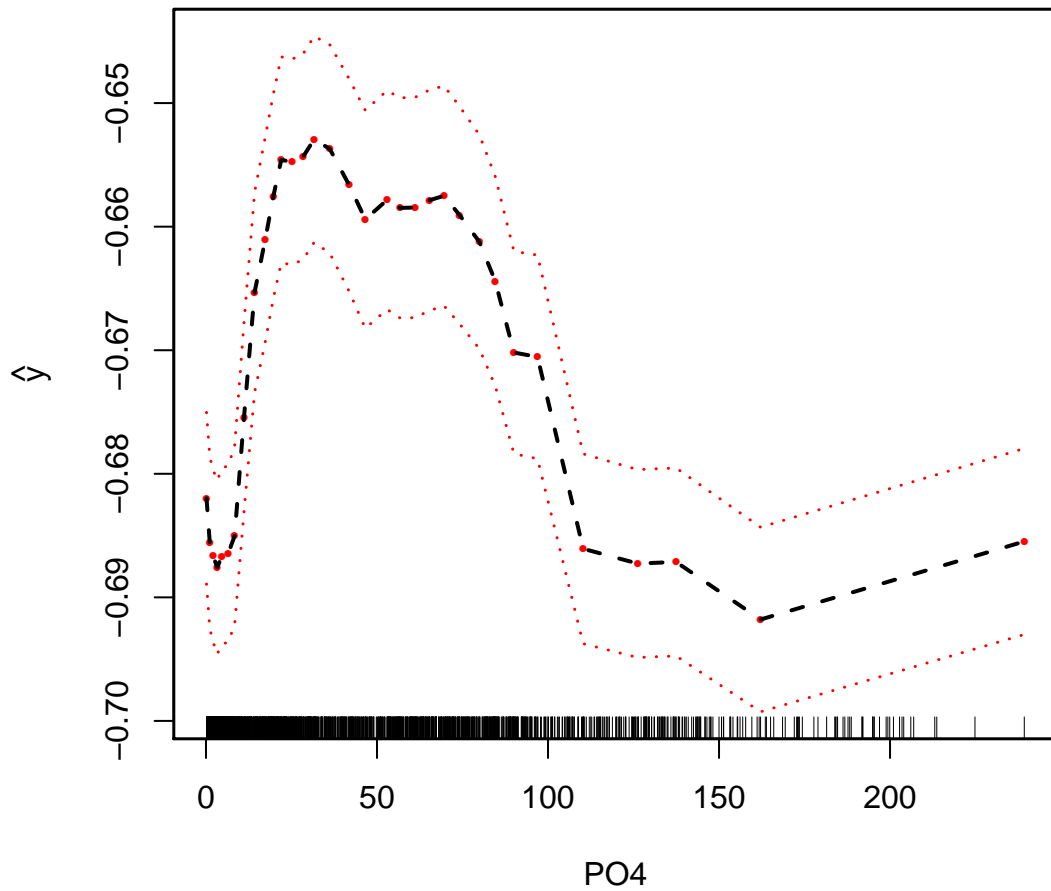

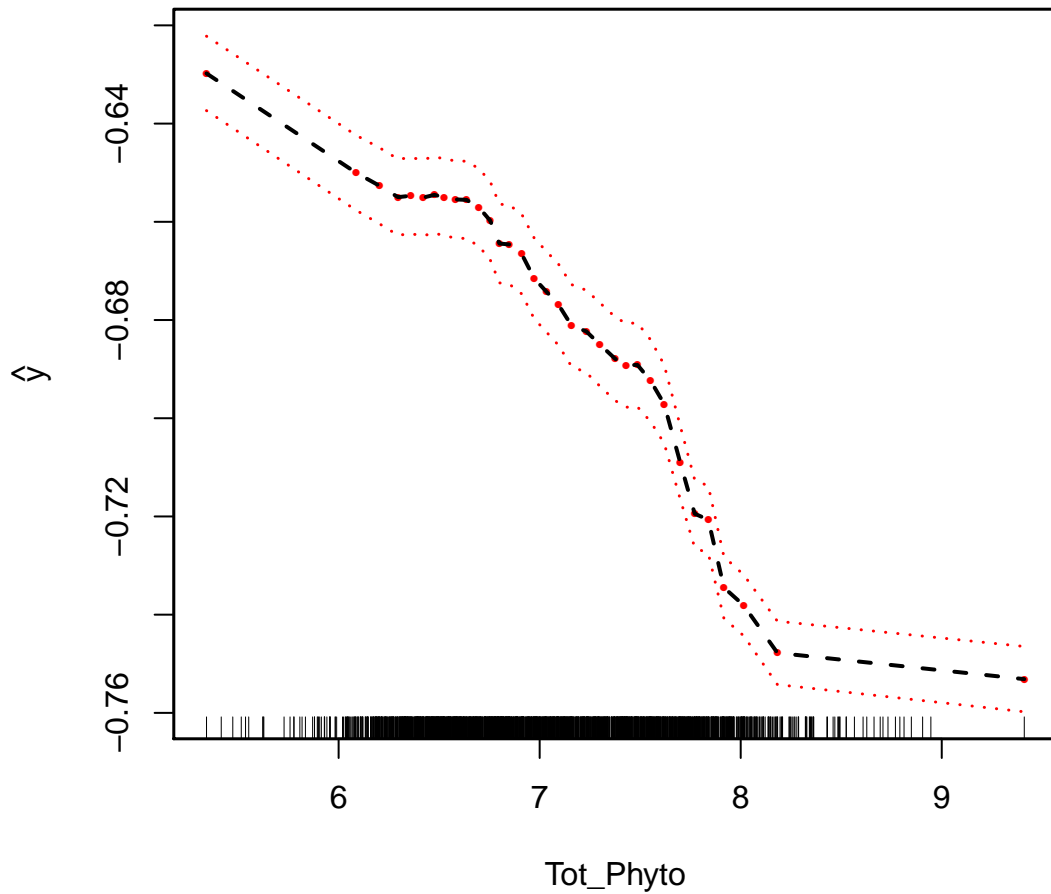

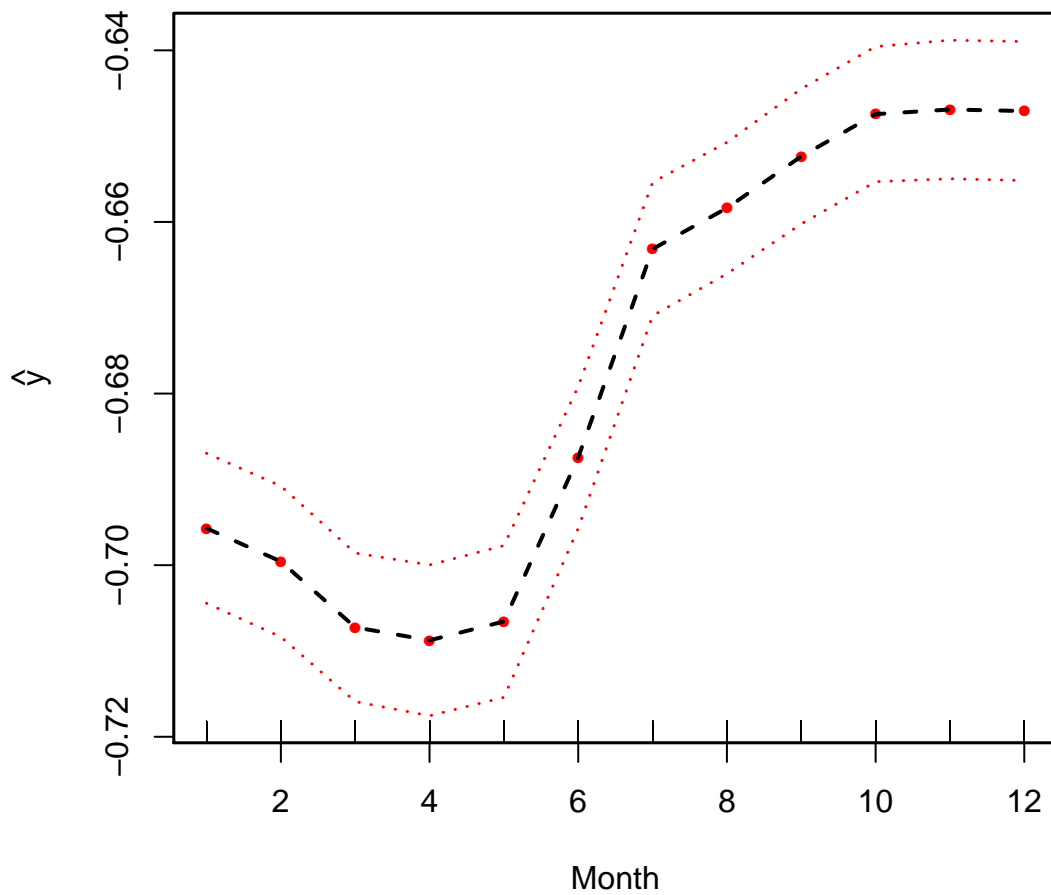

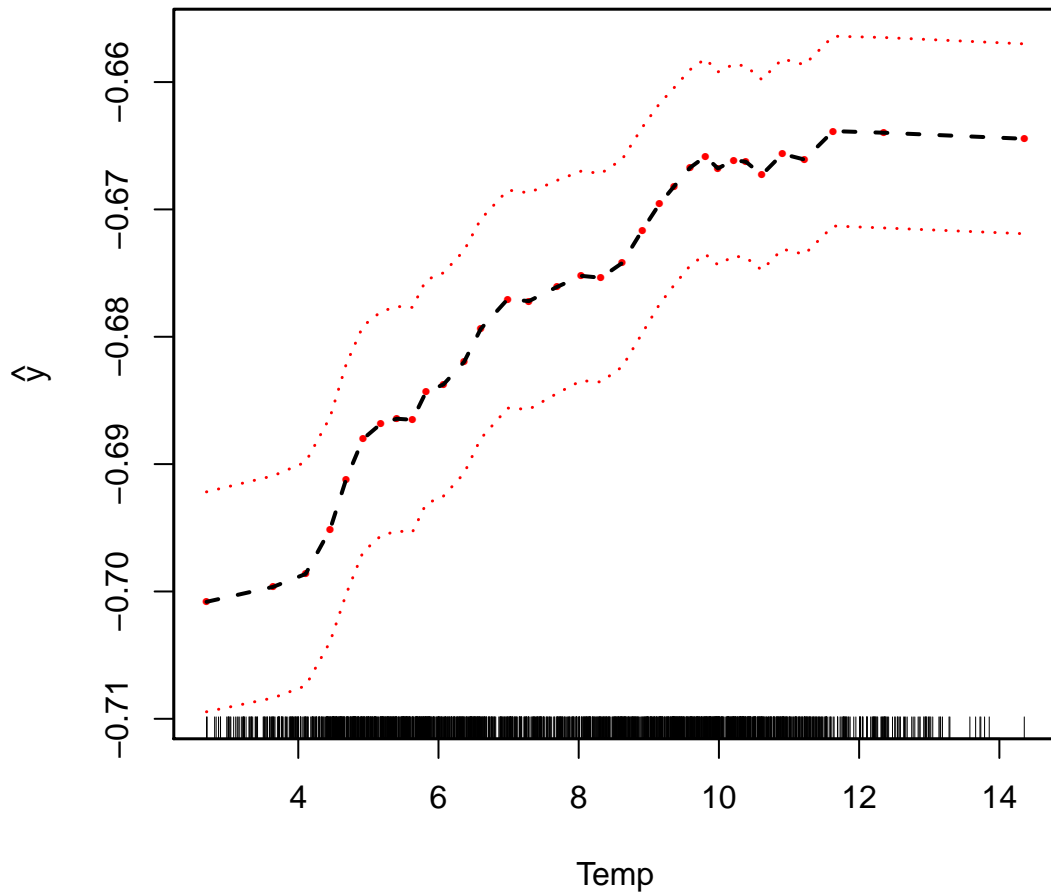

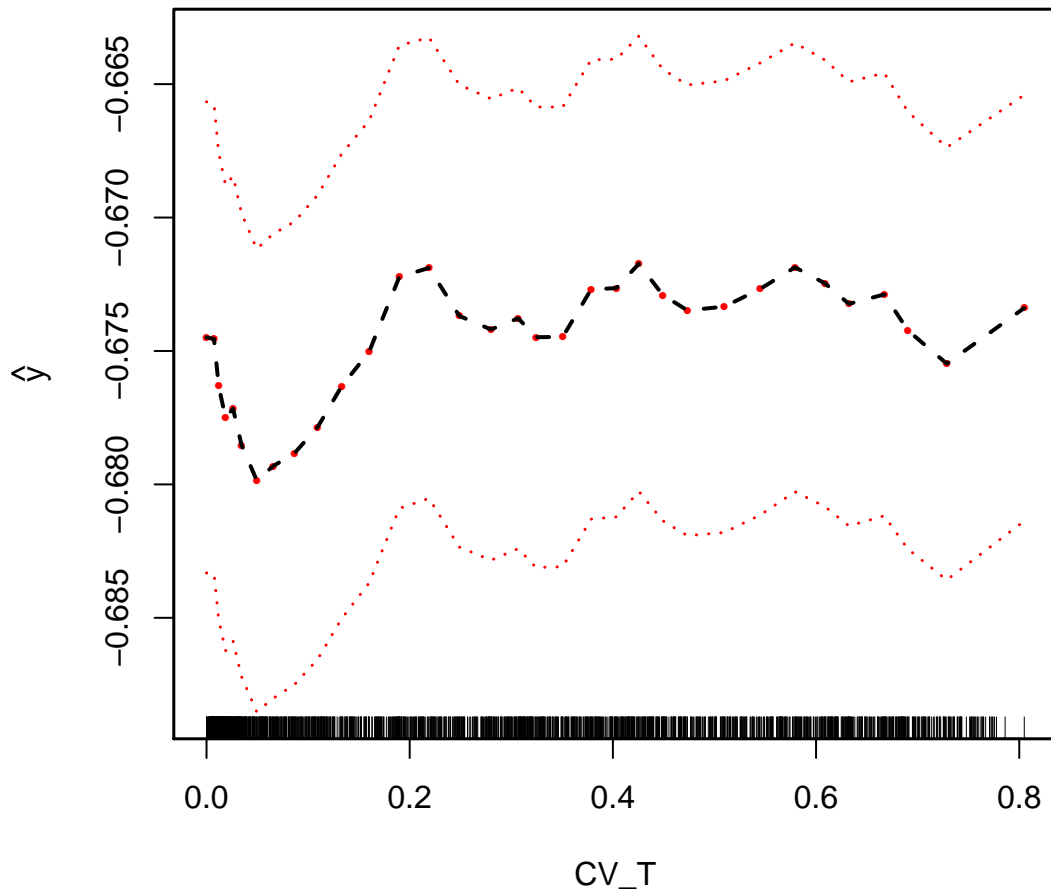

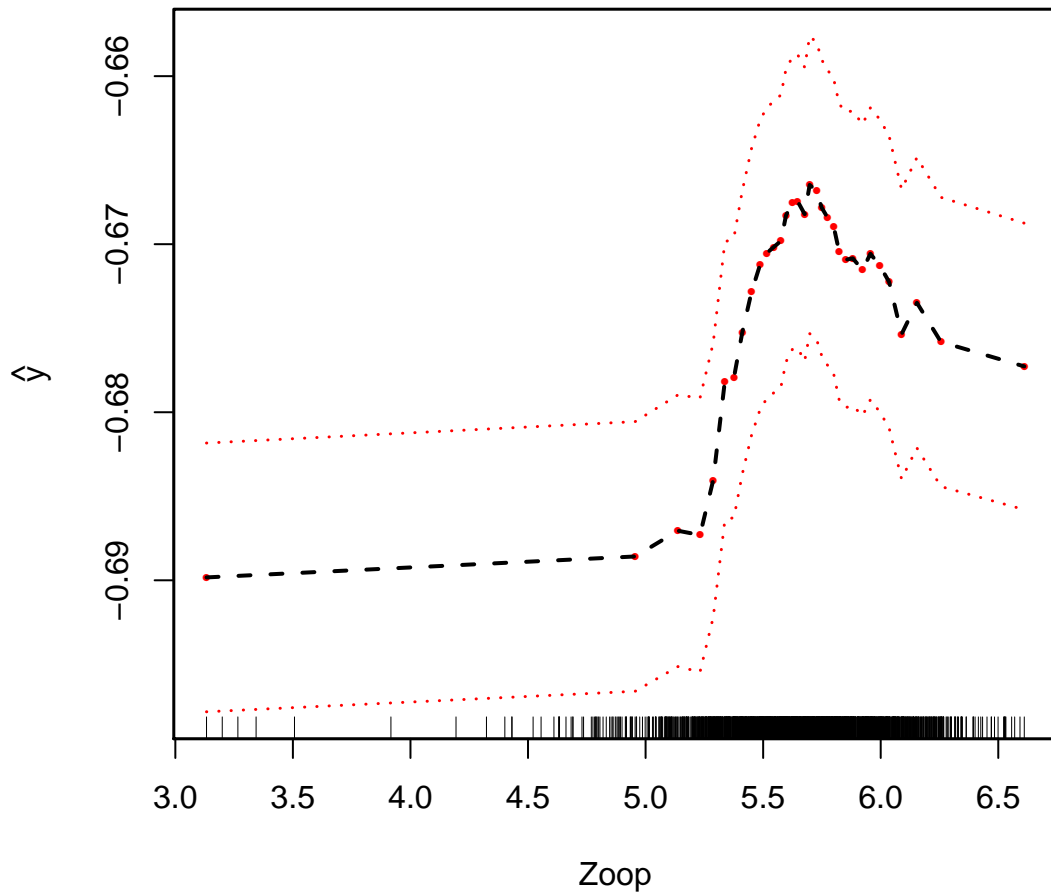

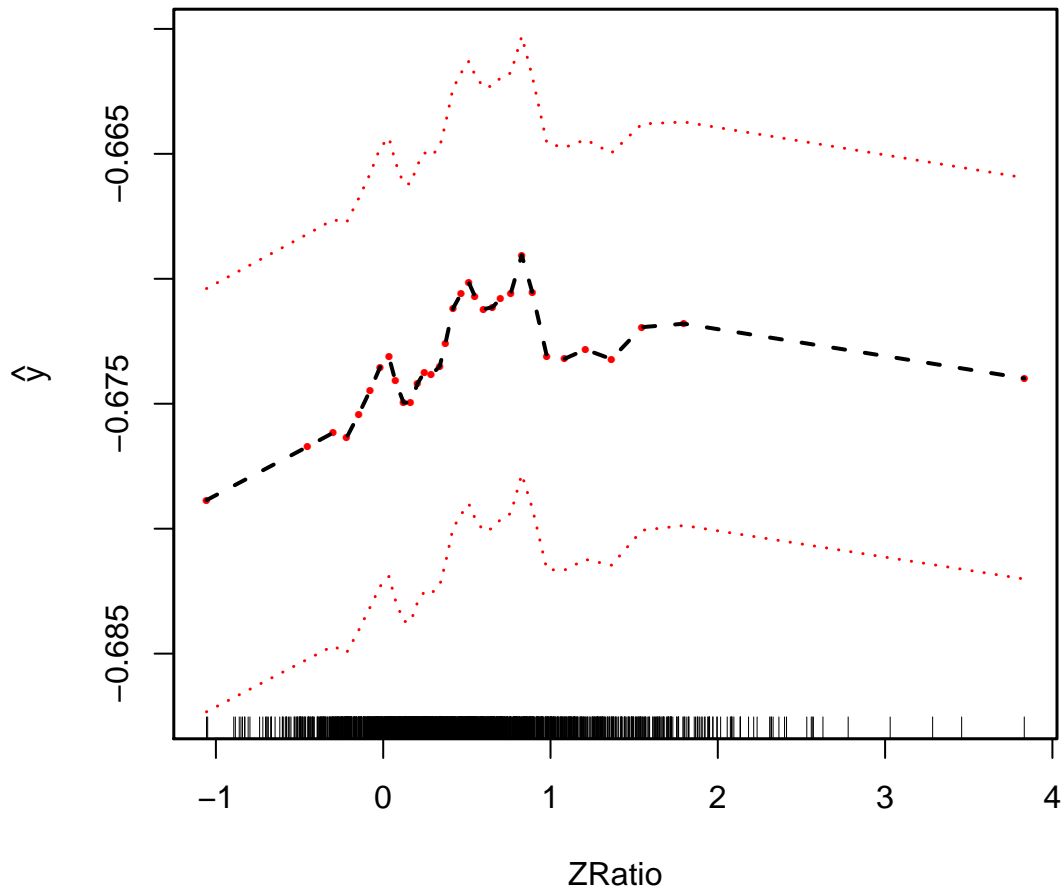

Supplement: FIGURE S1 — Scaling of phytoplankton abundances (Log10 cells L−1) with size (Log10 taxa biovolumes) in each lake dataset. [file Presentation_1.ZIP › FigS12.rf_noCyanos.pdf]

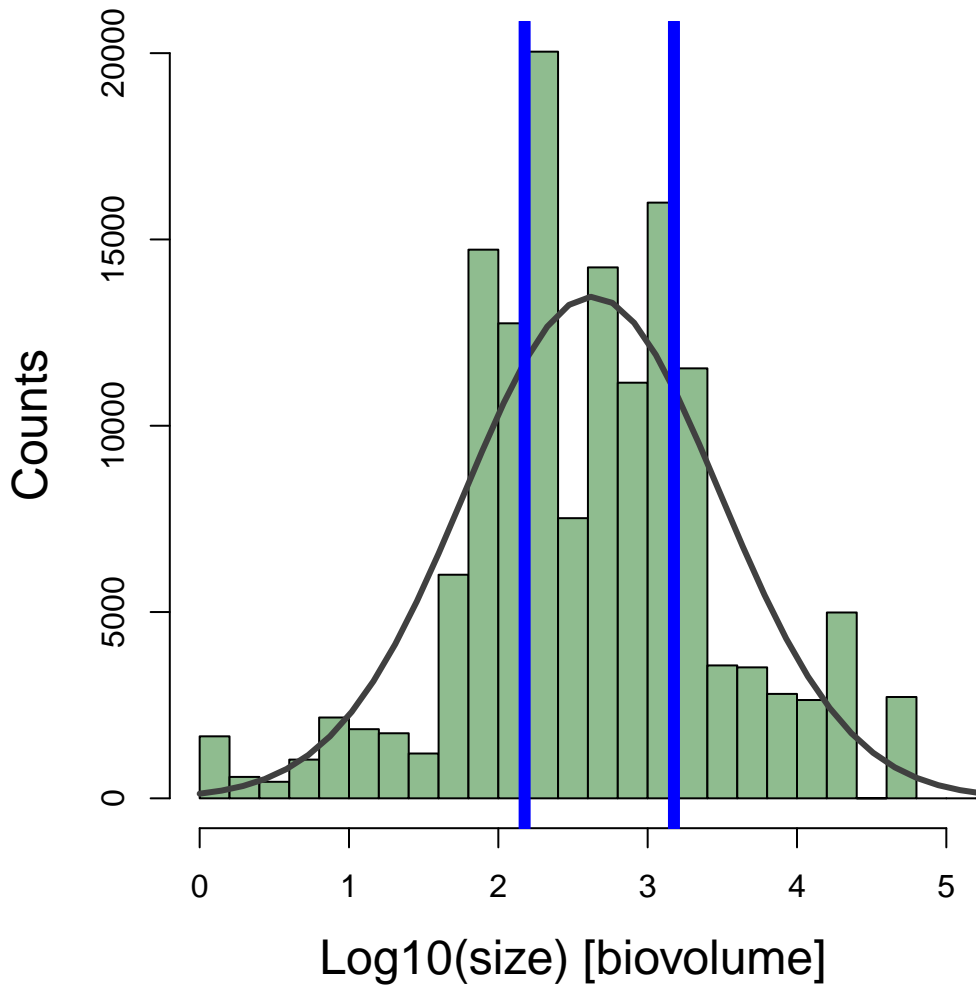

Supplement: FIGURE S1 — Scaling of phytoplankton abundances (Log10 cells L−1) with size (Log10 taxa biovolumes) in each lake dataset. [file Presentation_1.ZIP › FigS2.size_distribution_alldata.pdf]

**% variance explained: 52.14**

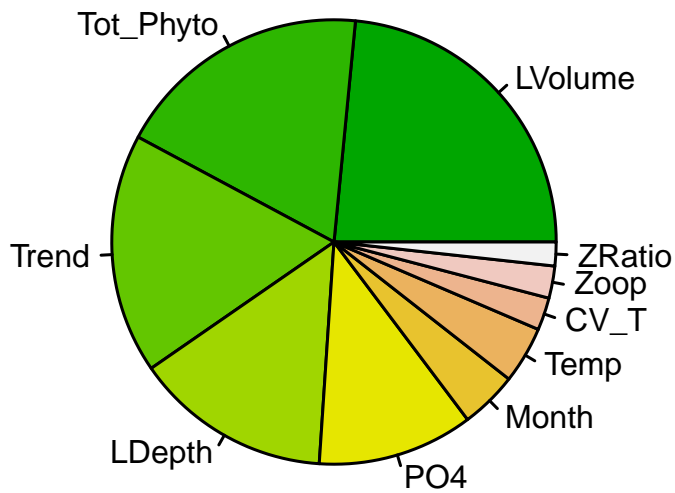

Supplement: FIGURE S1 — Scaling of phytoplankton abundances (Log10 cells L−1) with size (Log10 taxa biovolumes) in each lake dataset. [file Presentation_1.ZIP › FigS3.rf_observed_relimp.pdf]

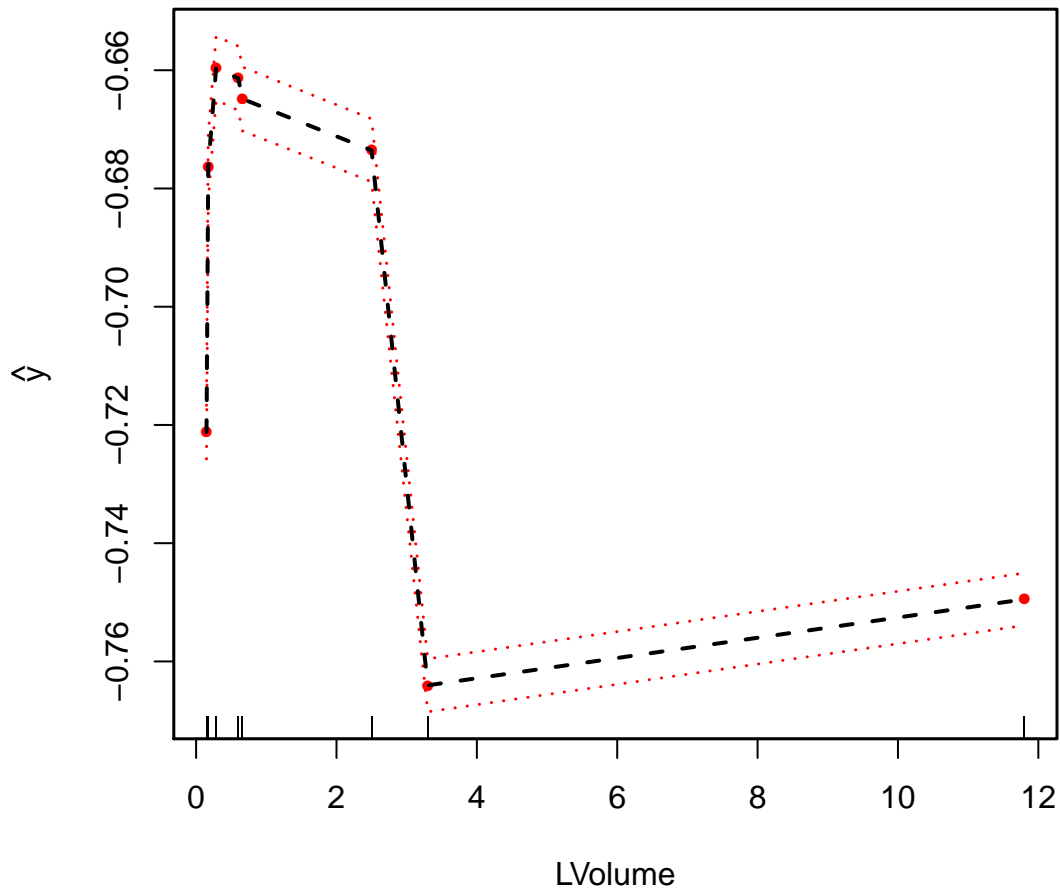

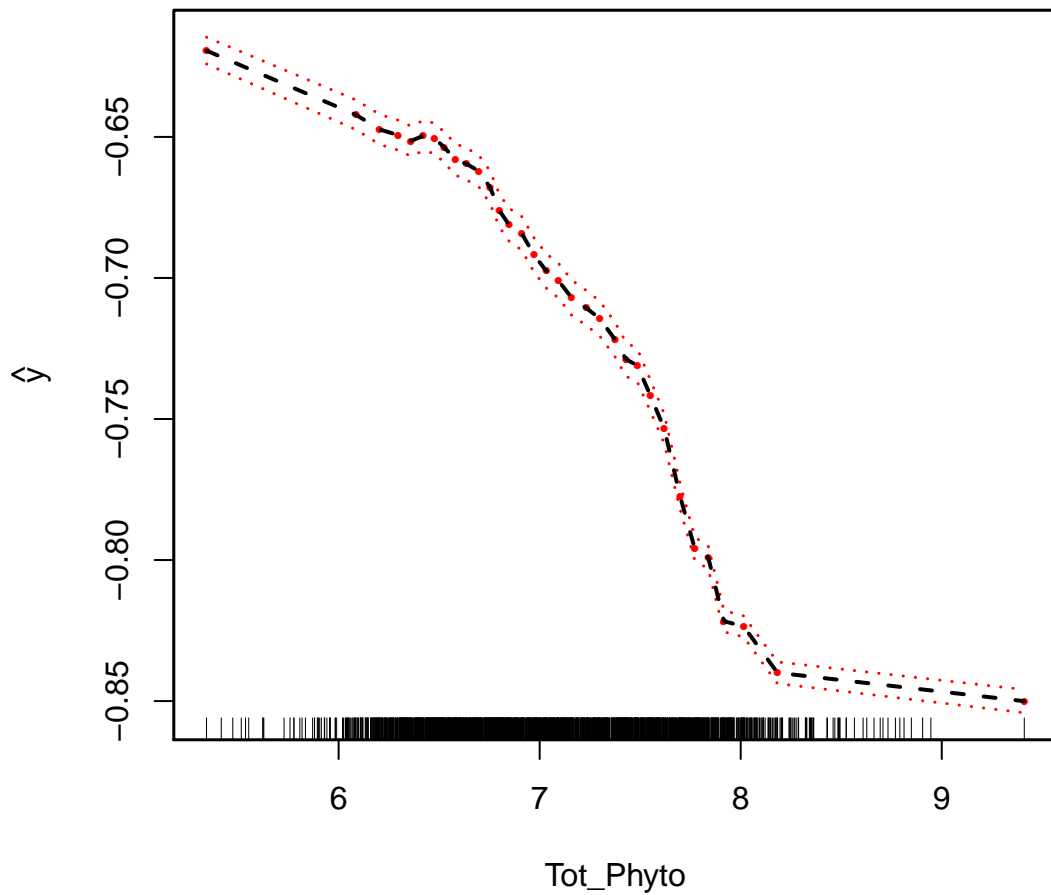

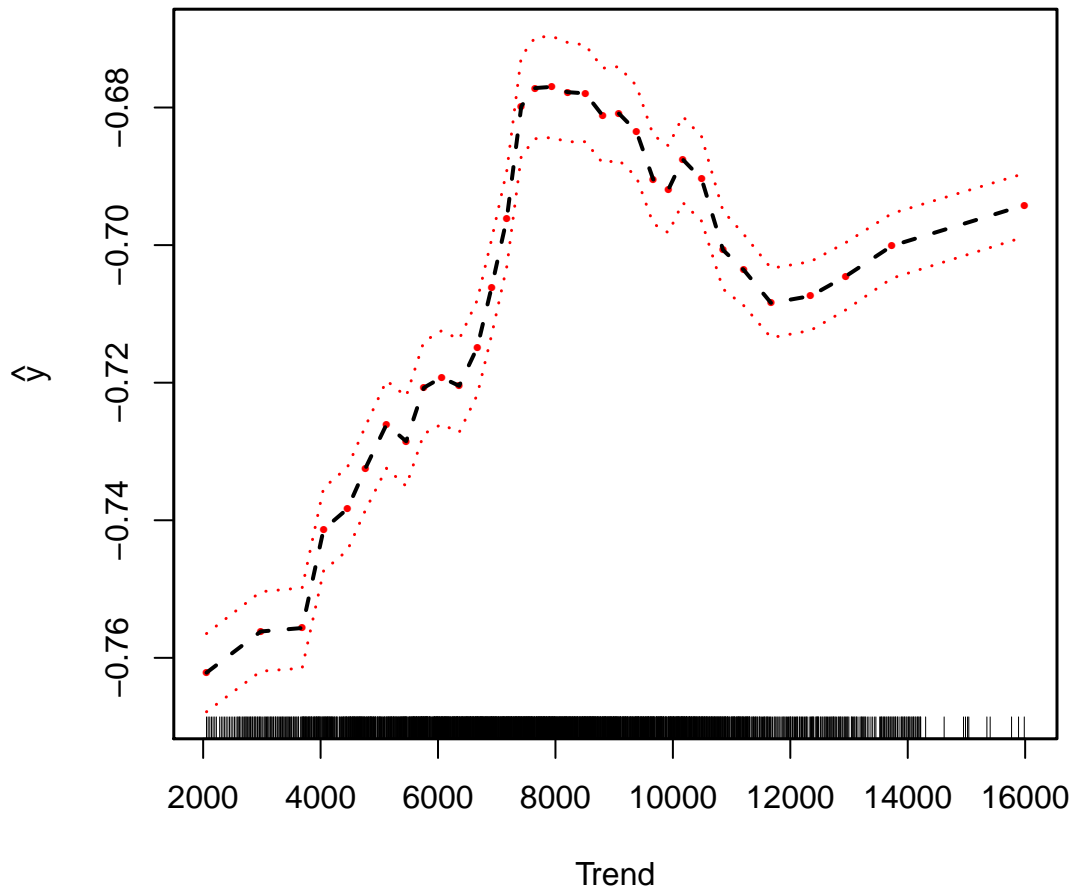

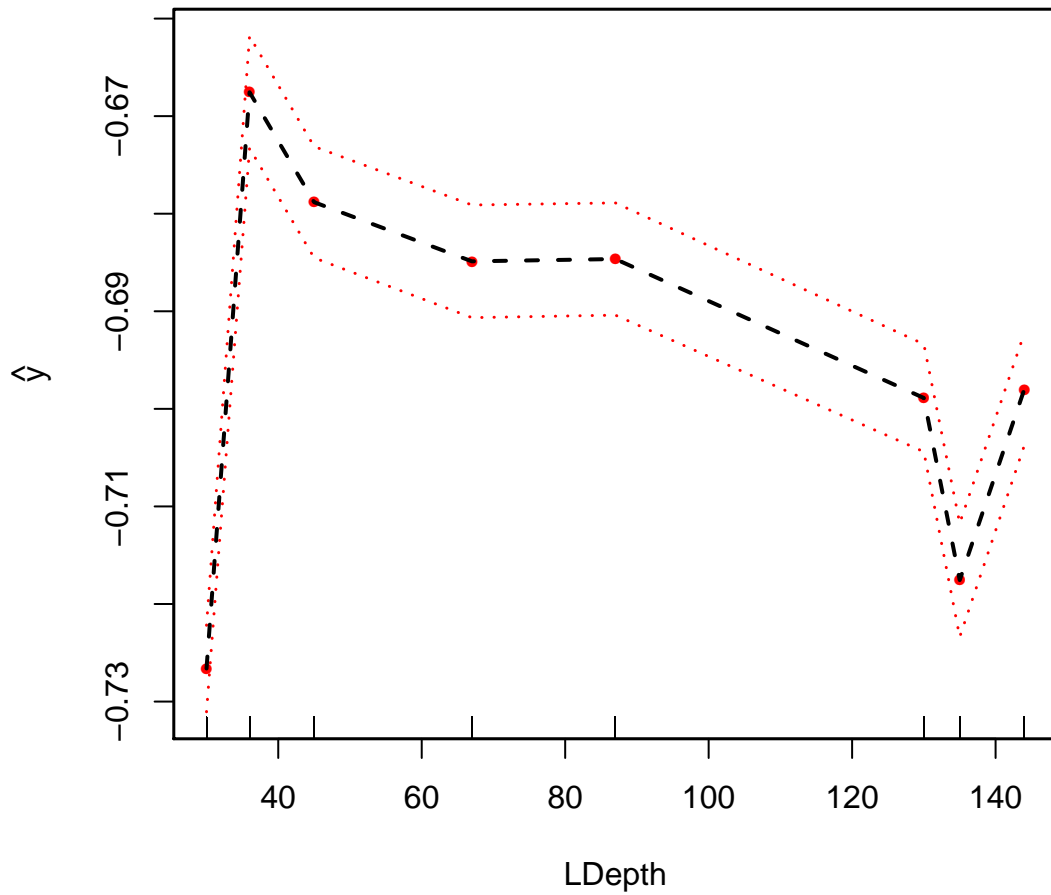

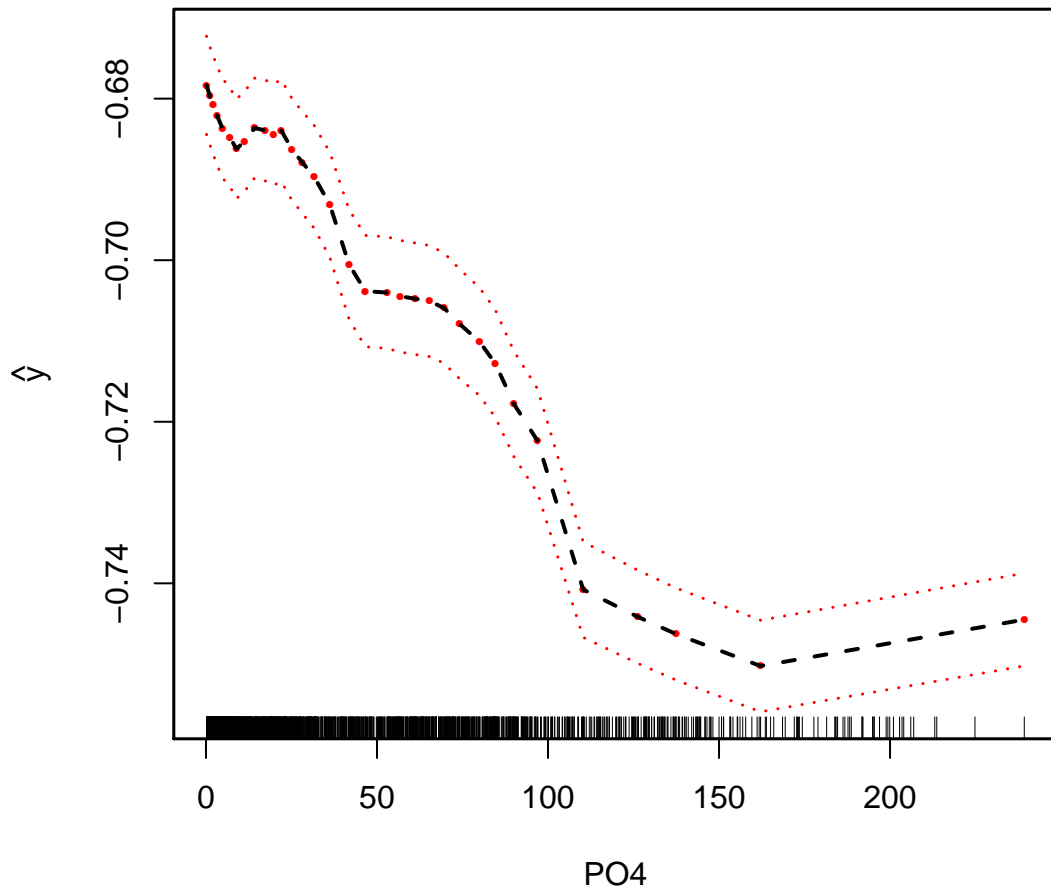

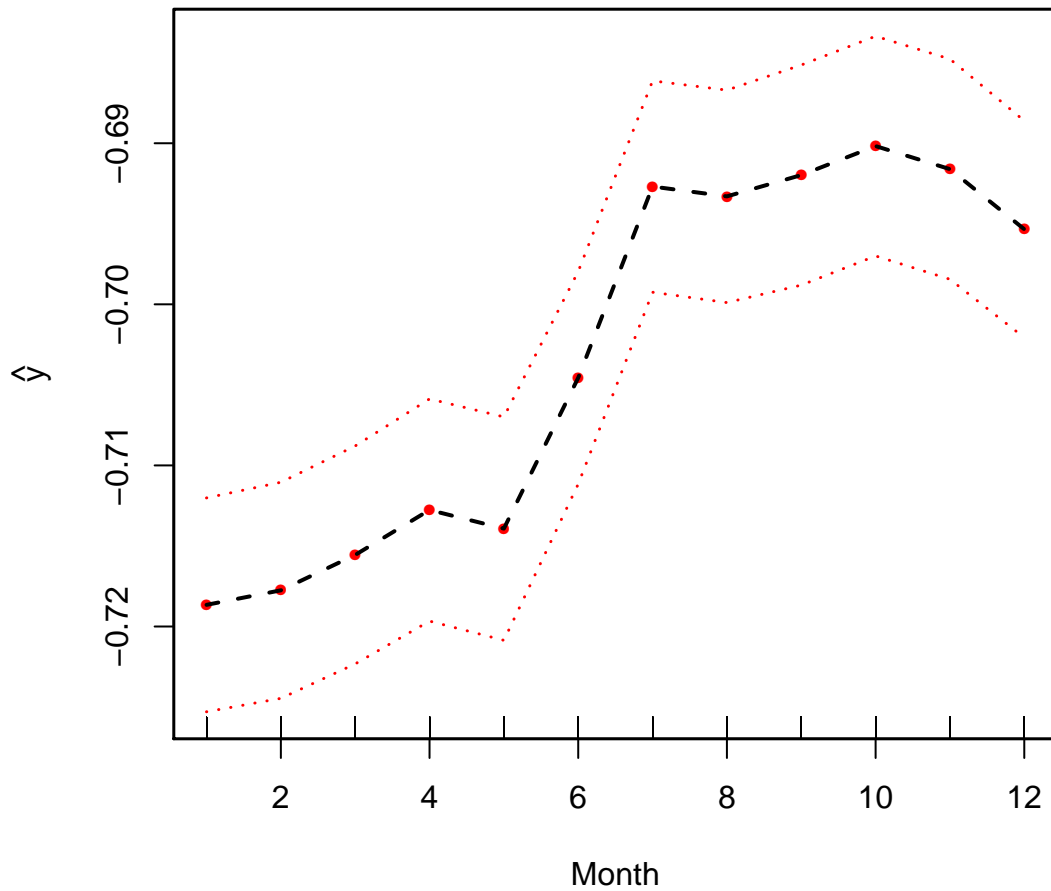

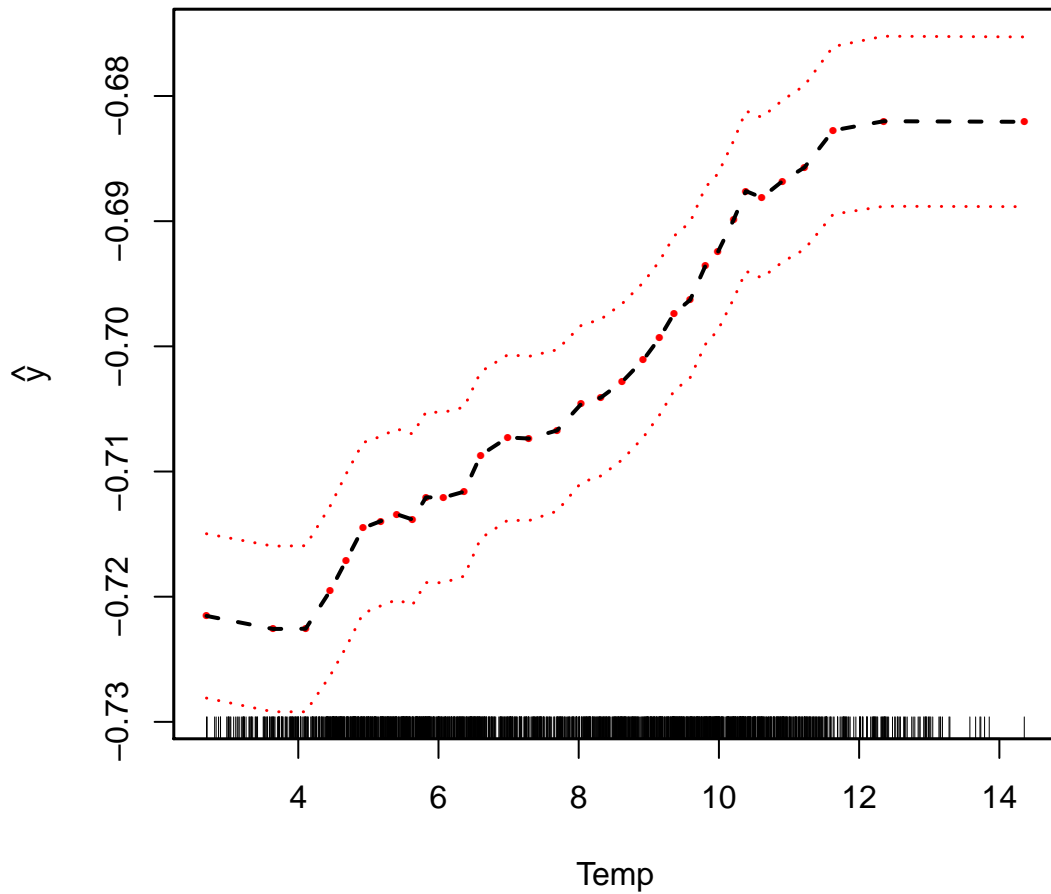

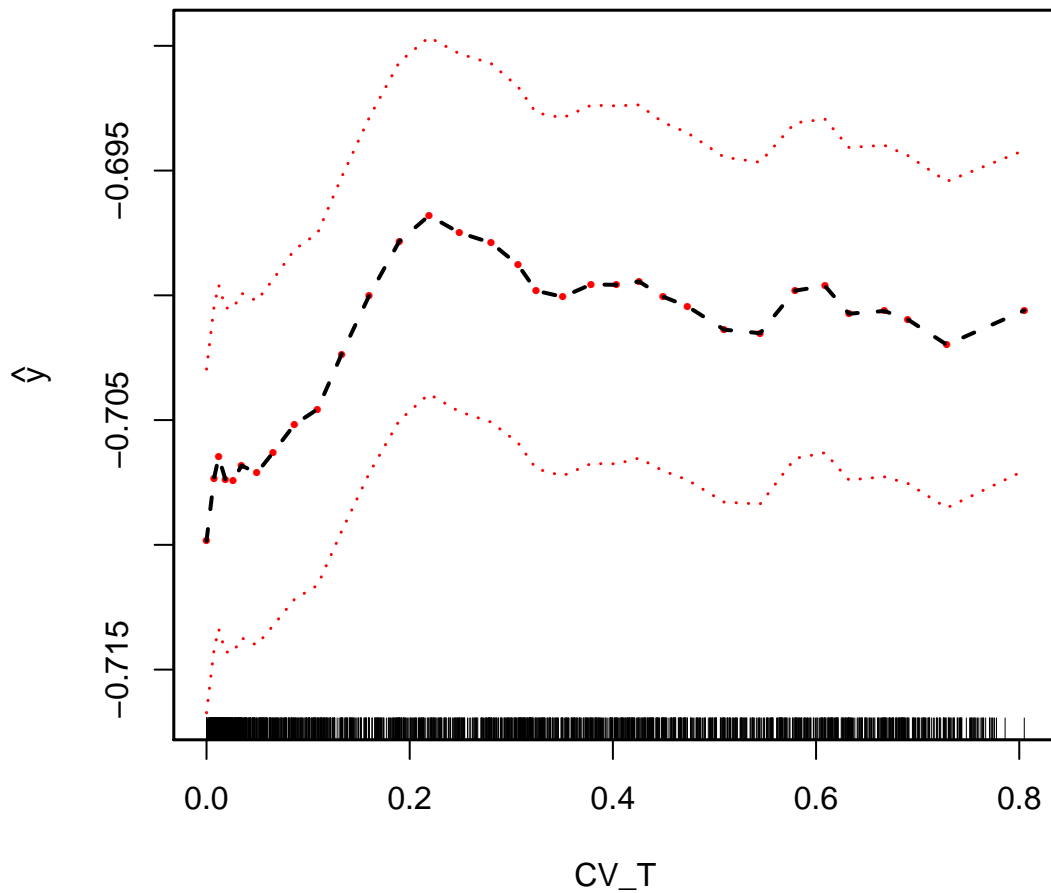

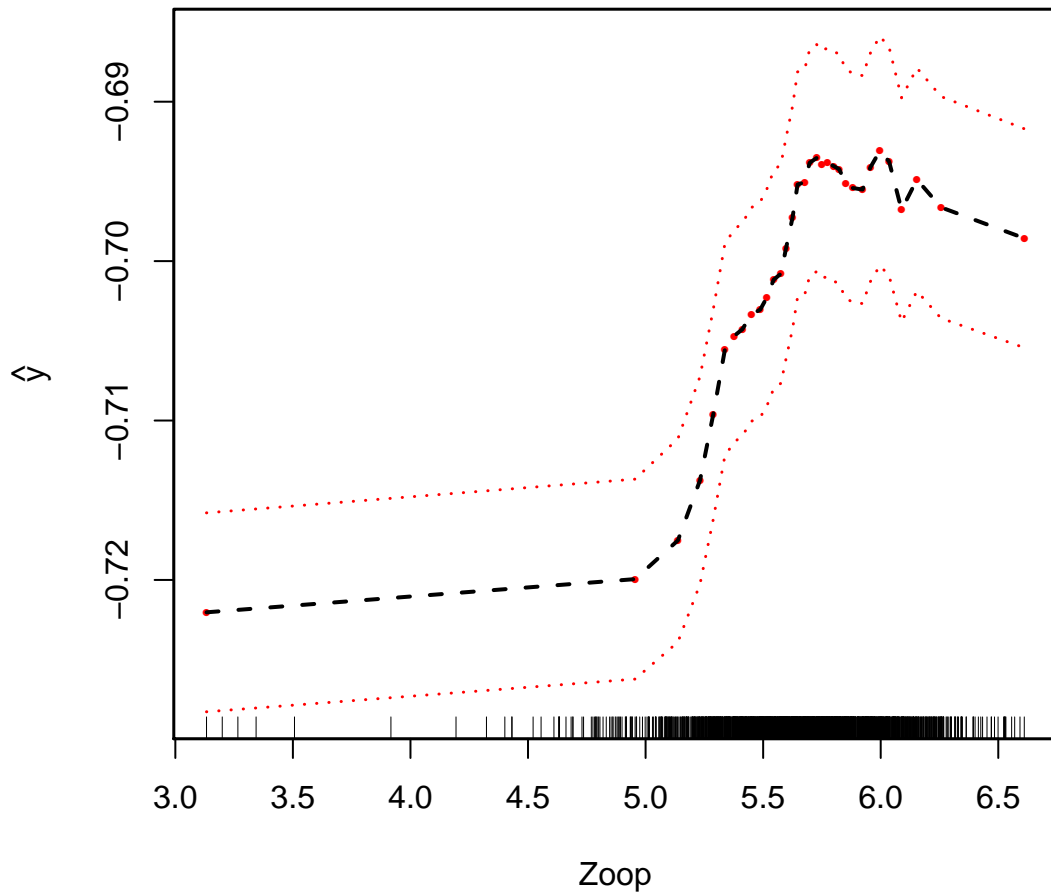

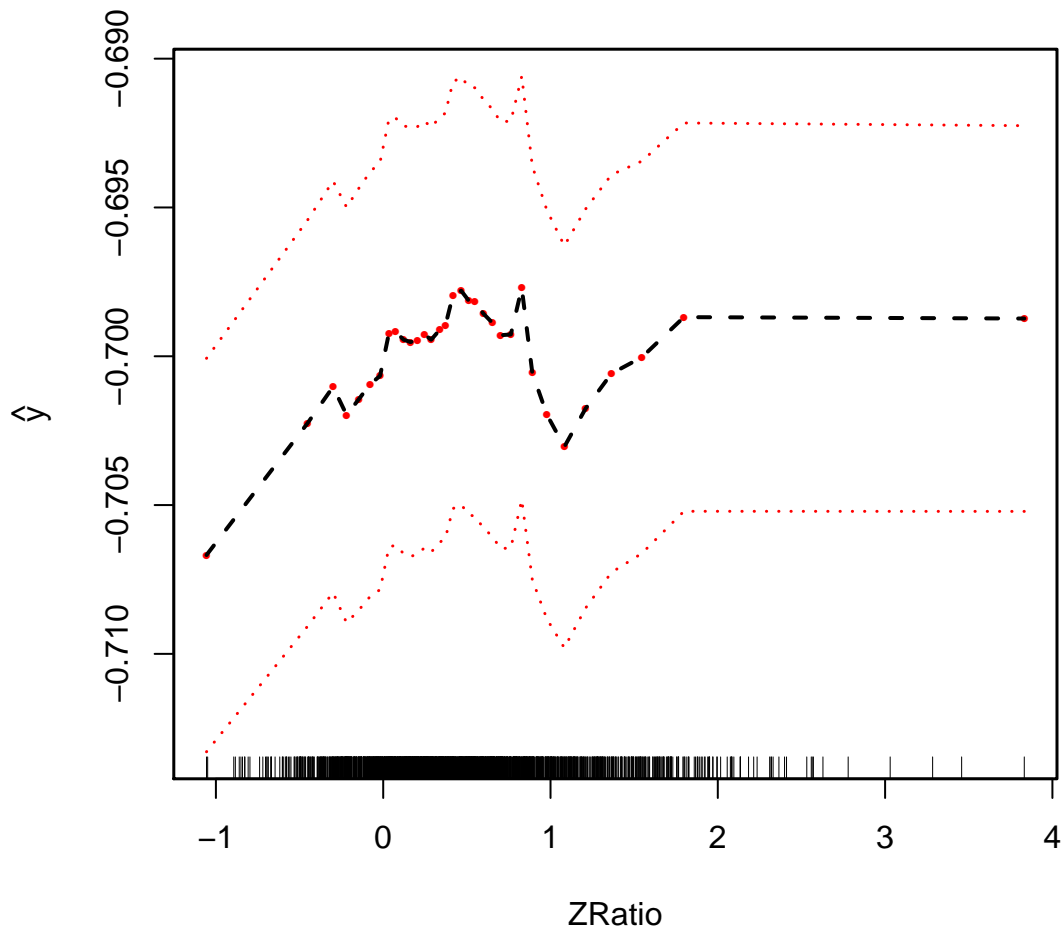

Supplement: FIGURE S1 — Scaling of phytoplankton abundances (Log10 cells L−1) with size (Log10 taxa biovolumes) in each lake dataset. [file Presentation_1.ZIP › FigS4.Effects_rf_observed.pdf]

**% variance explained: 52.4**

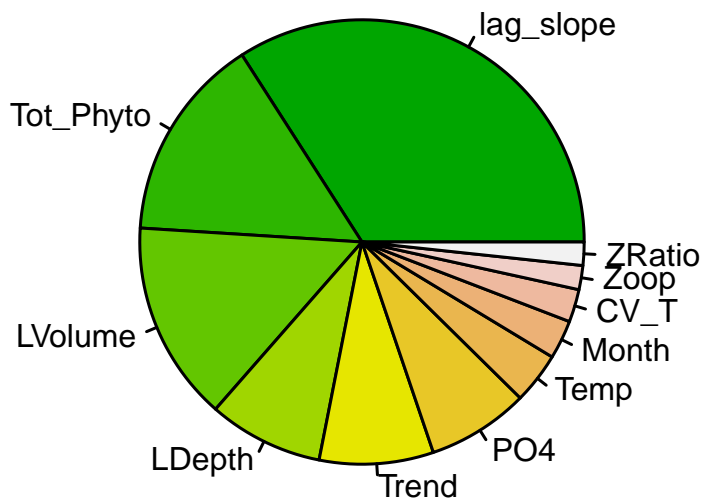

Supplement: FIGURE S1 — Scaling of phytoplankton abundances (Log10 cells L−1) with size (Log10 taxa biovolumes) in each lake dataset. [file Presentation_1.ZIP › FigS5.rf_withLag_relimp.pdf]

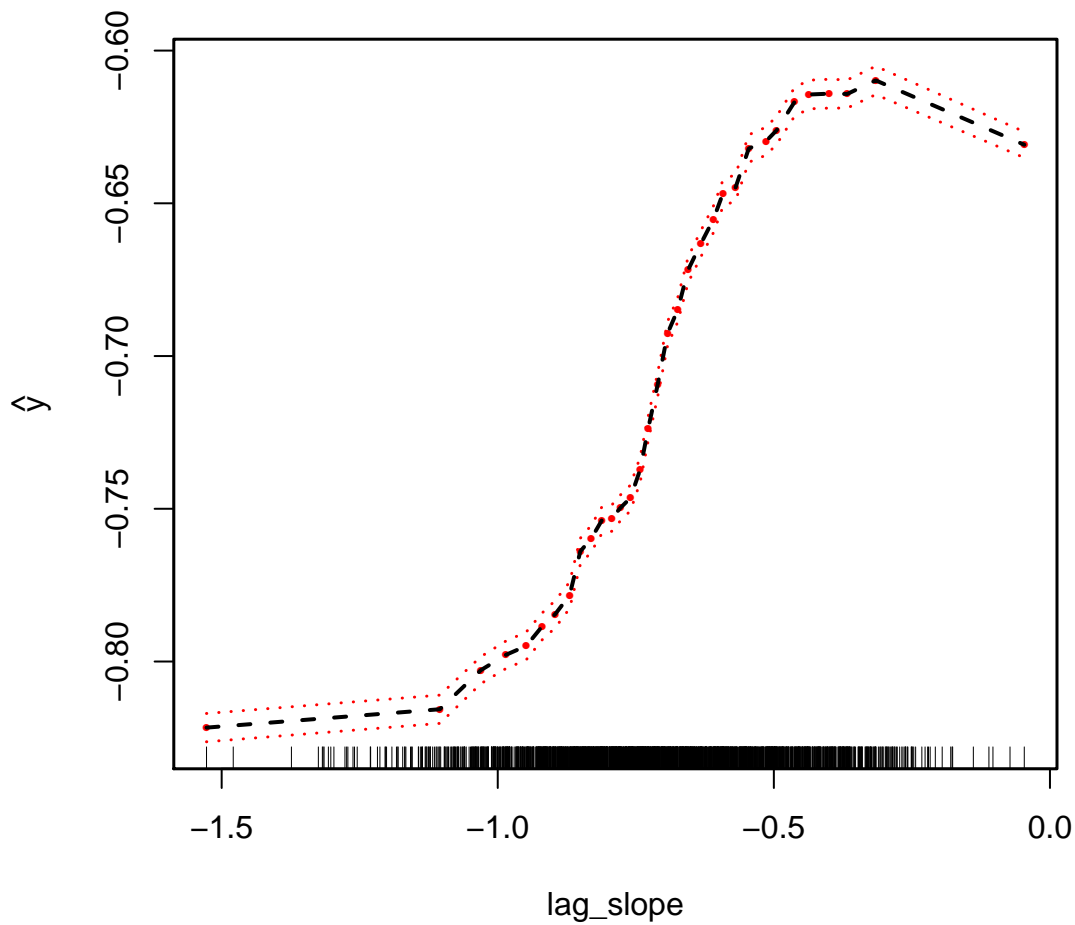

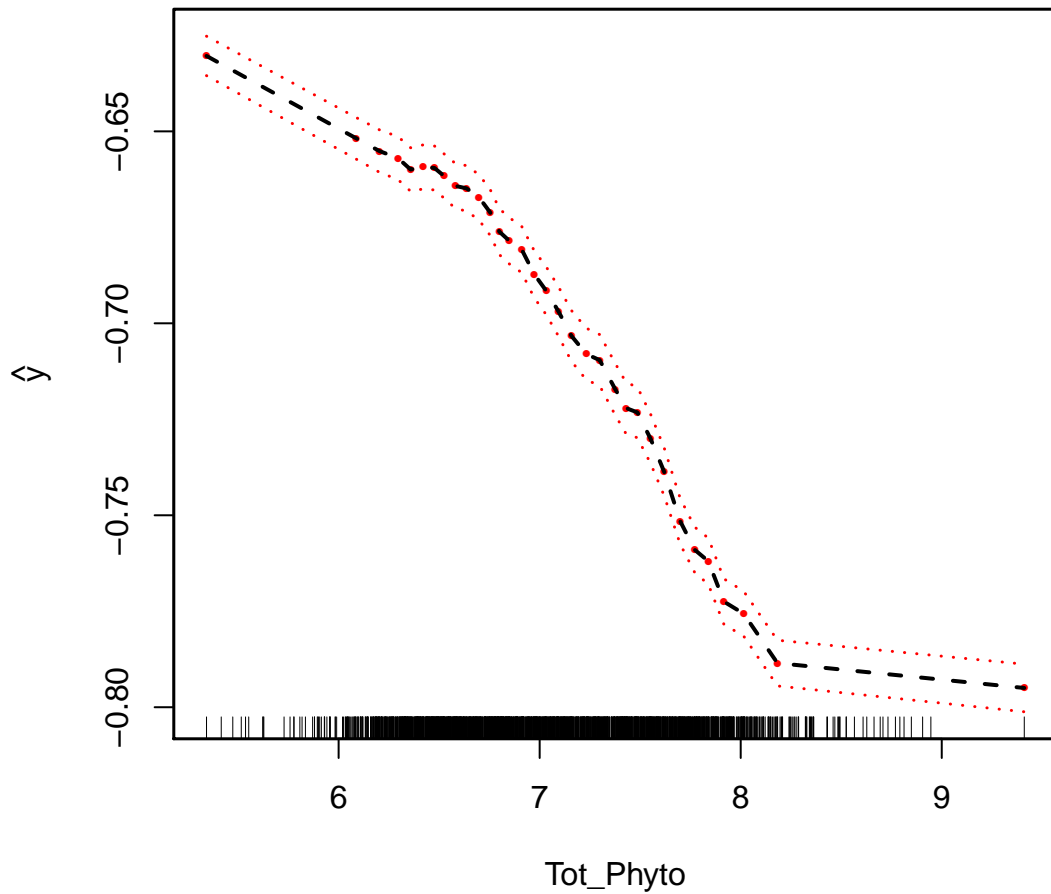

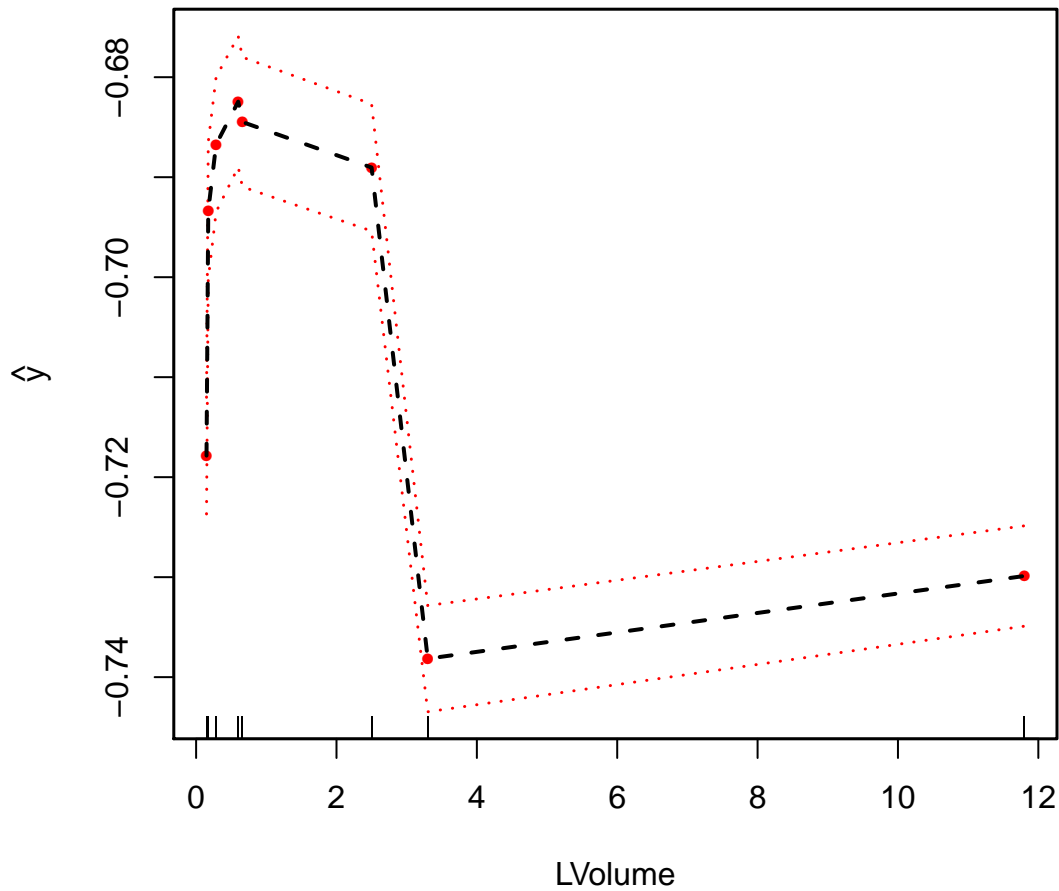

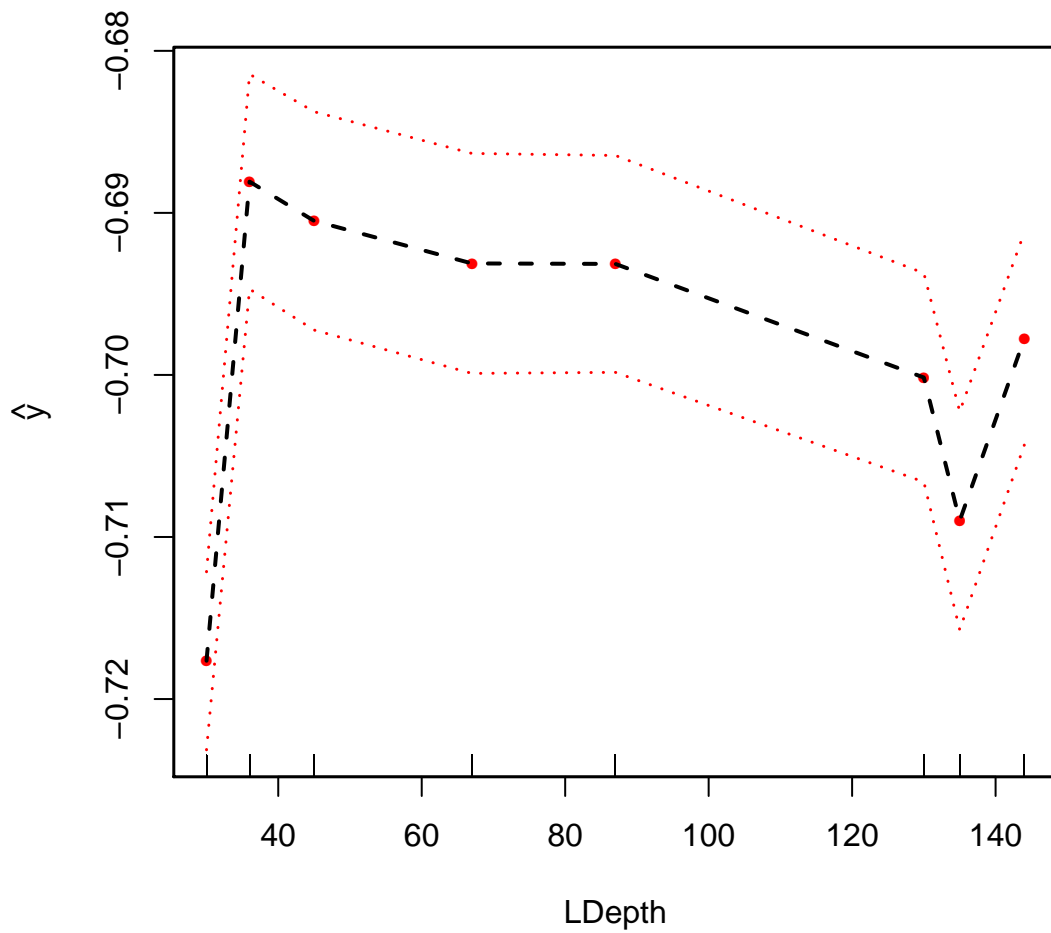

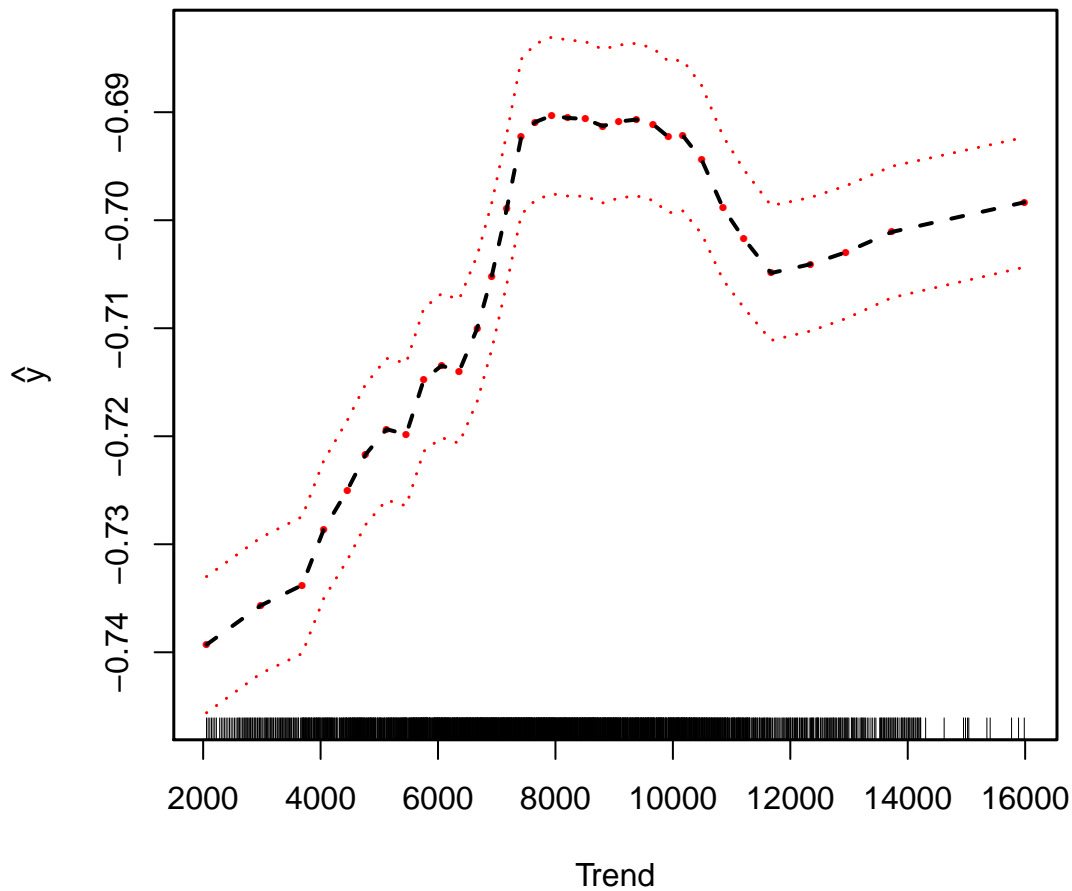

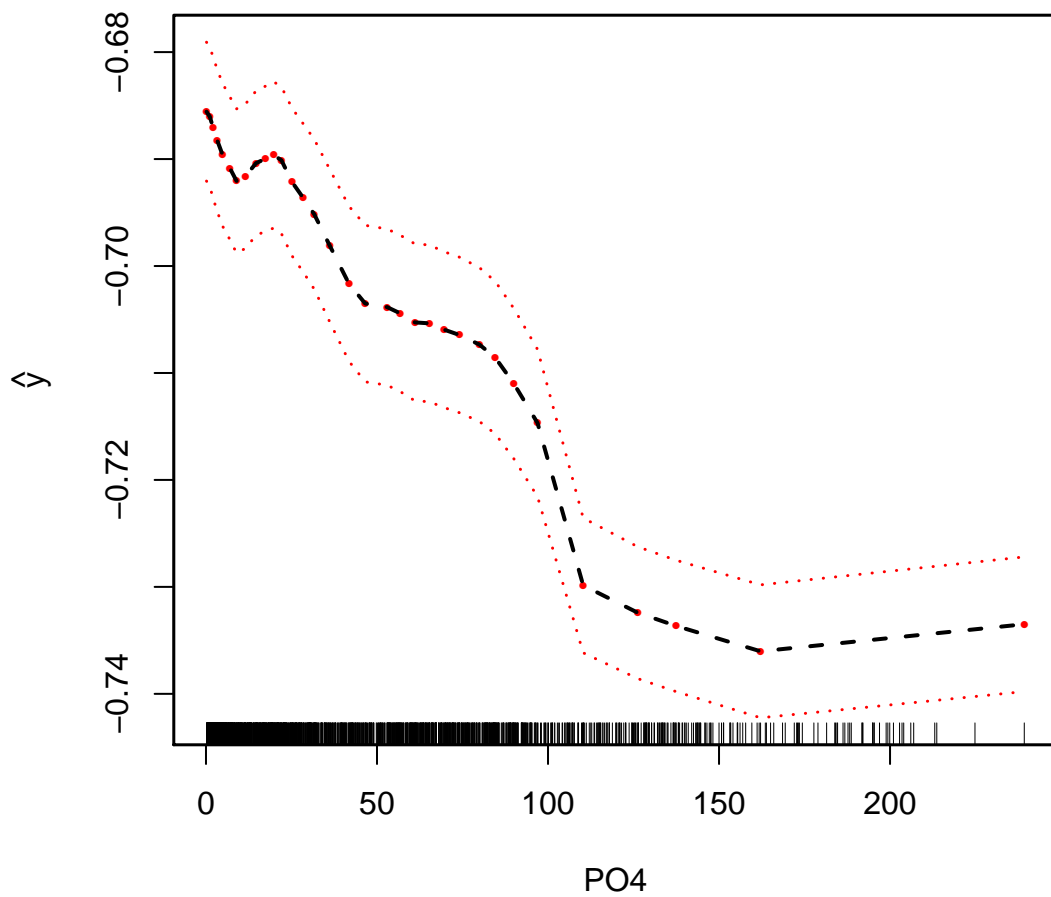

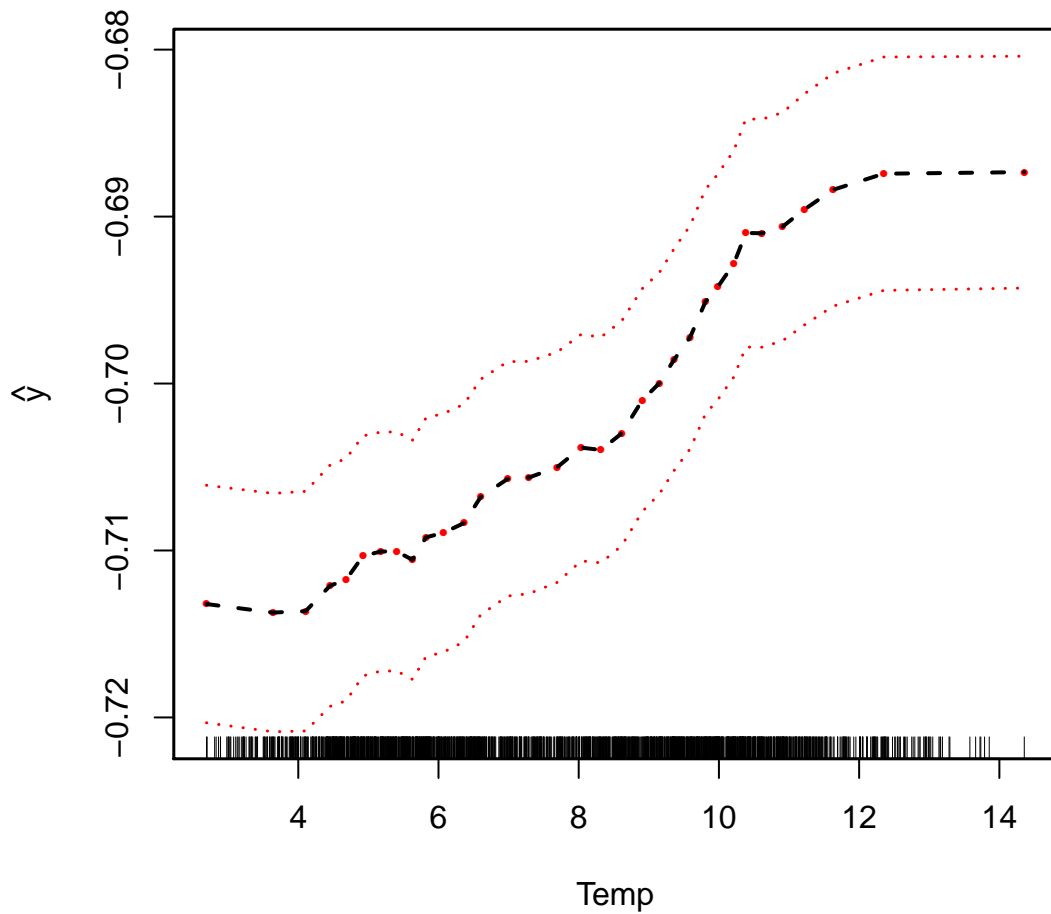

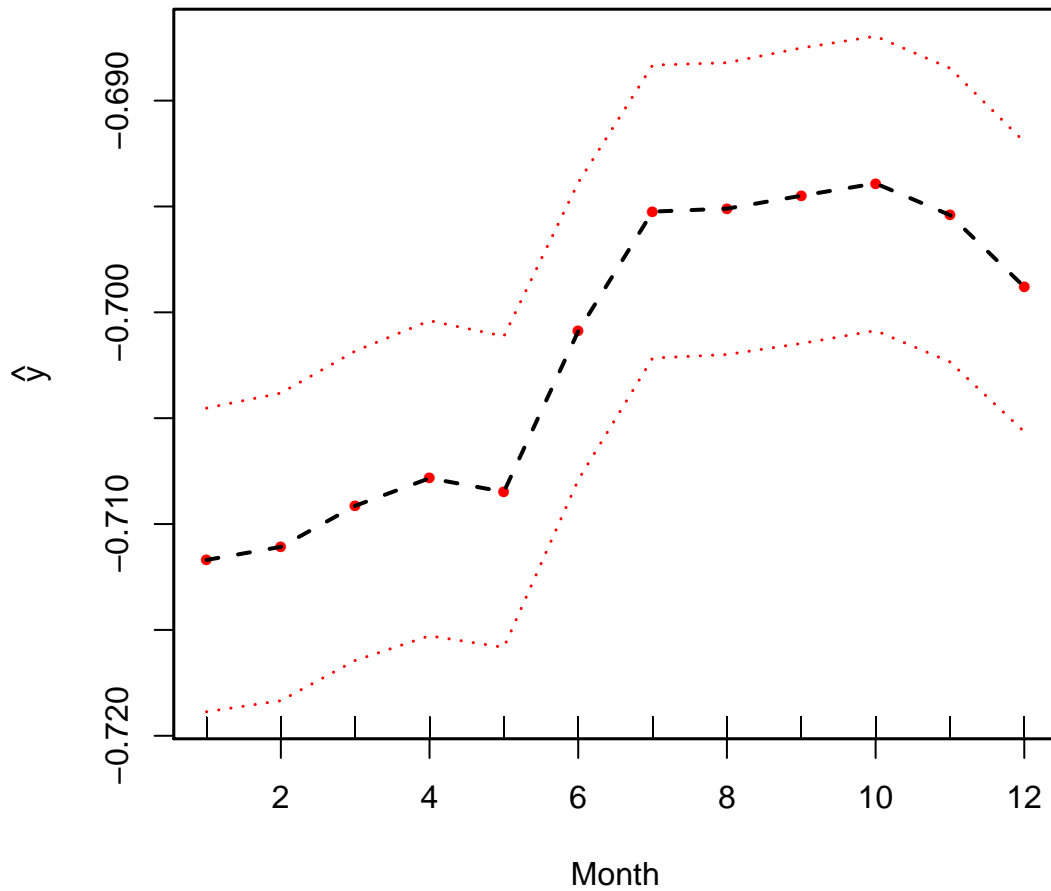

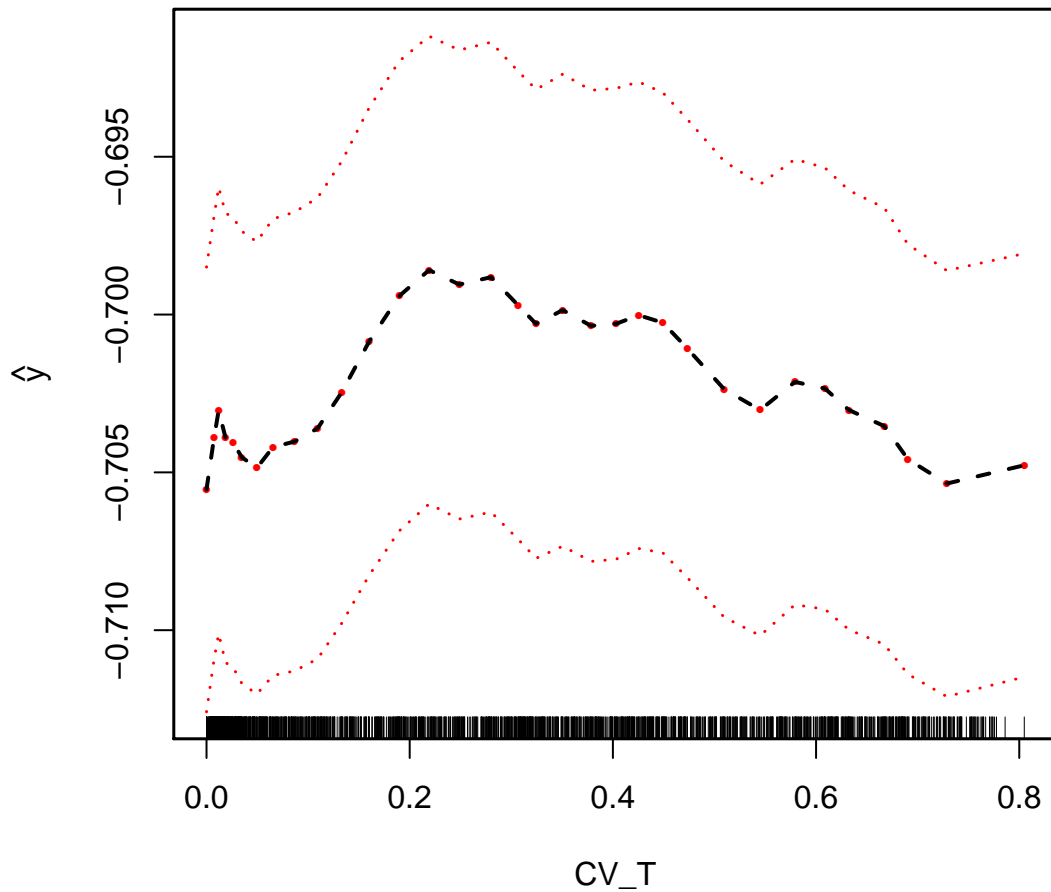

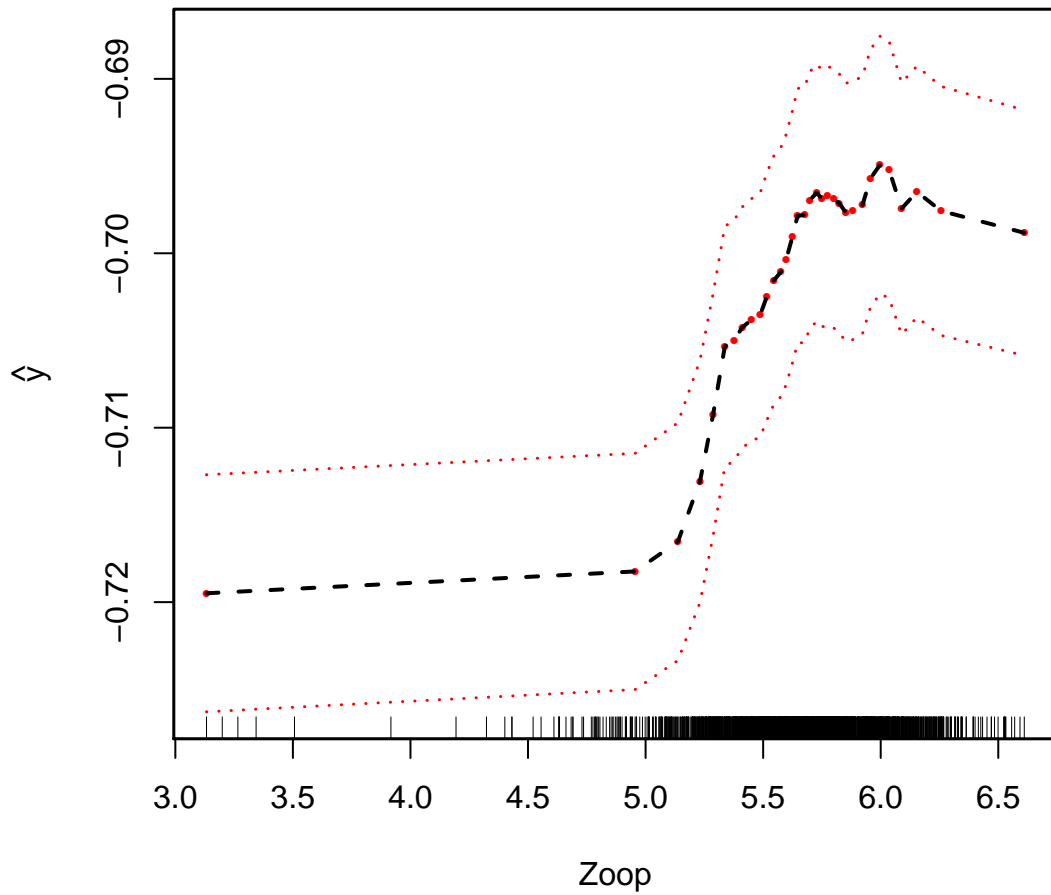

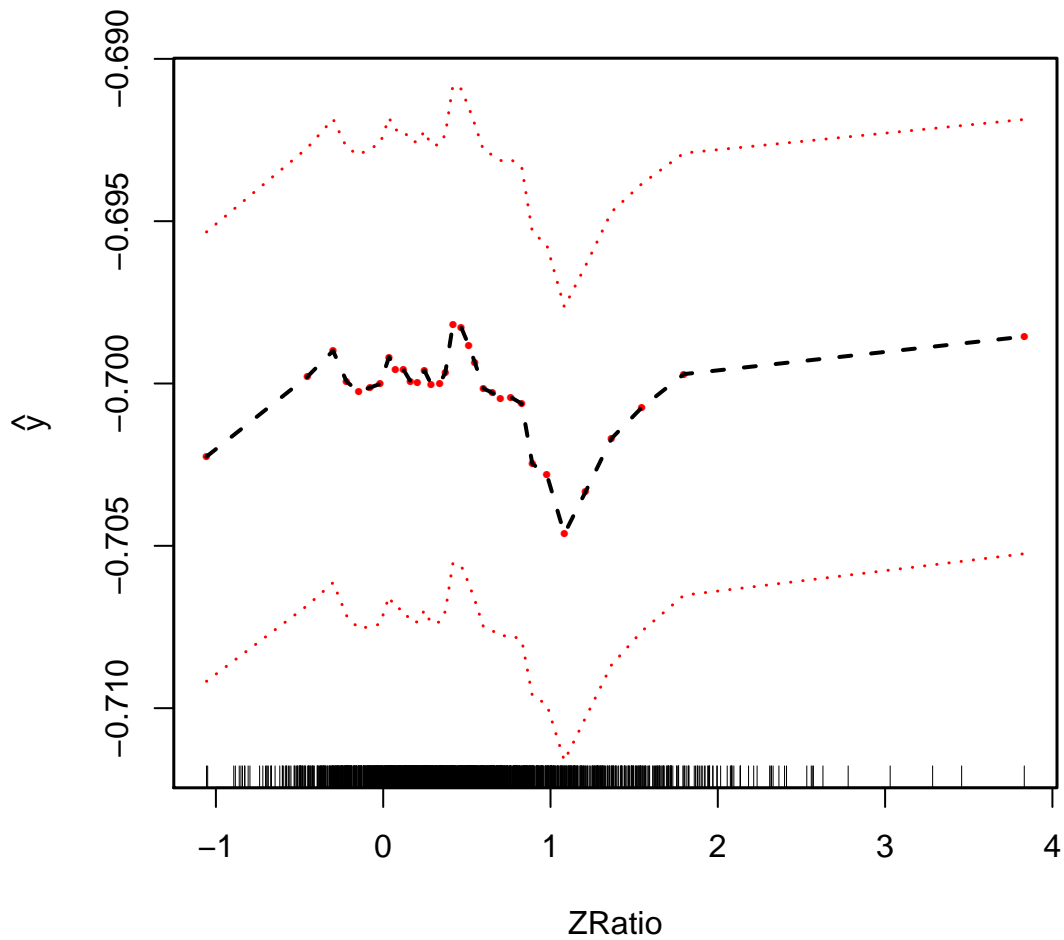

Supplement: FIGURE S1 — Scaling of phytoplankton abundances (Log10 cells L−1) with size (Log10 taxa biovolumes) in each lake dataset. [file Presentation_1.ZIP › FigS6.Effecs_rf_withLag.pdf]

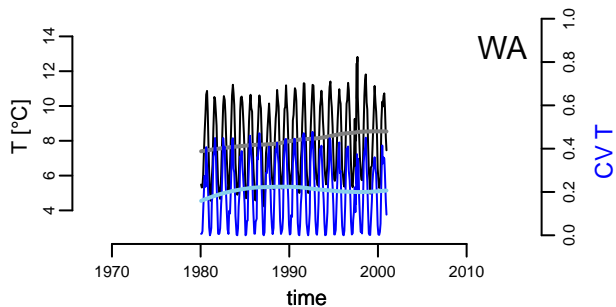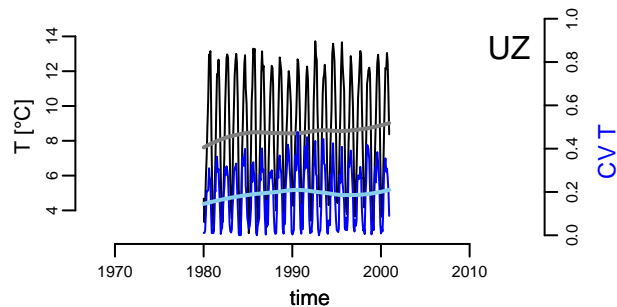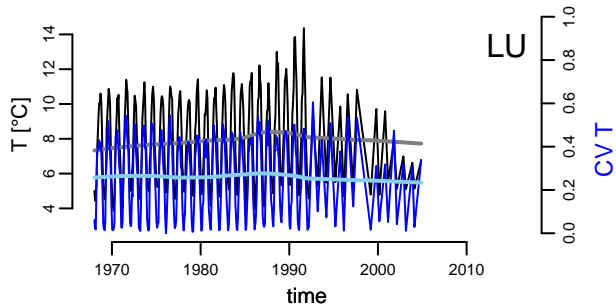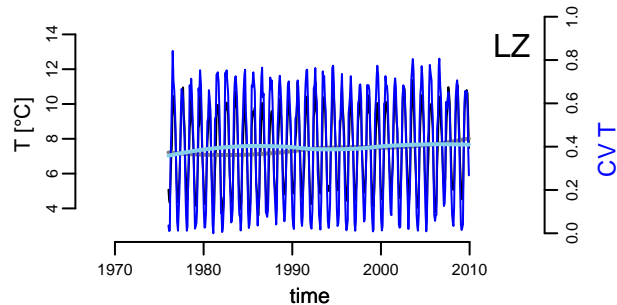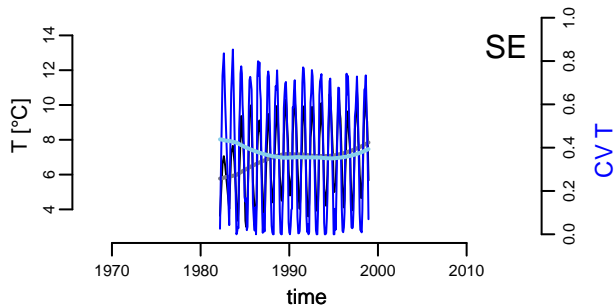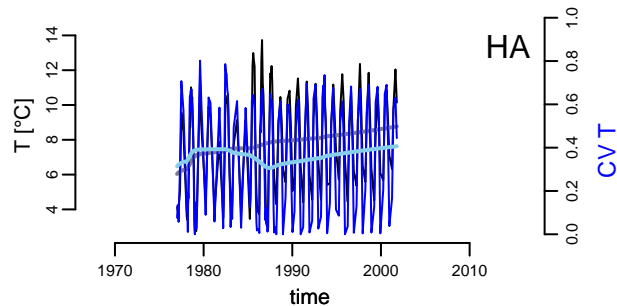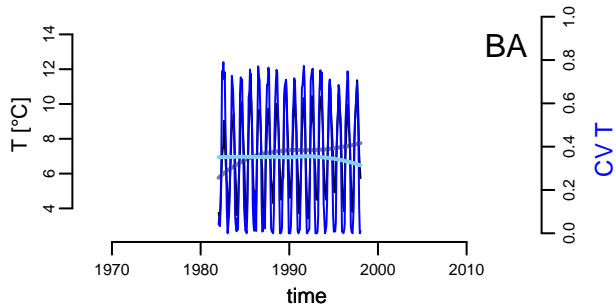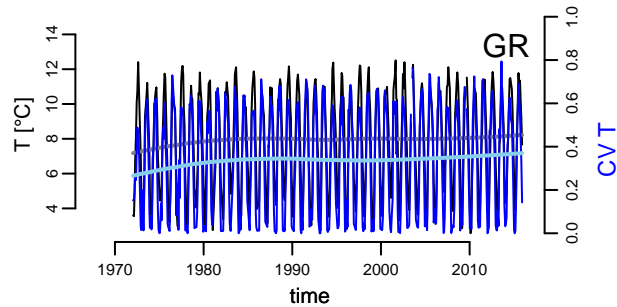

Supplement: FIGURE S1 — Scaling of phytoplankton abundances (Log10 cells L−1) with size (Log10 taxa biovolumes) in each lake dataset. [file Presentation_1.ZIP › FigS7.mean_t&cv.pdf]

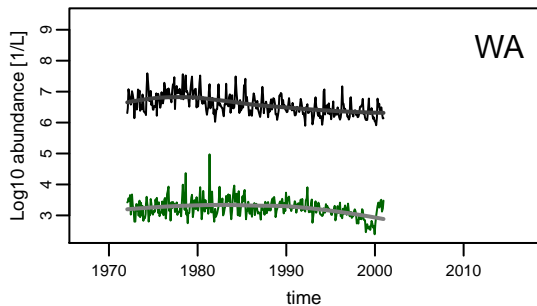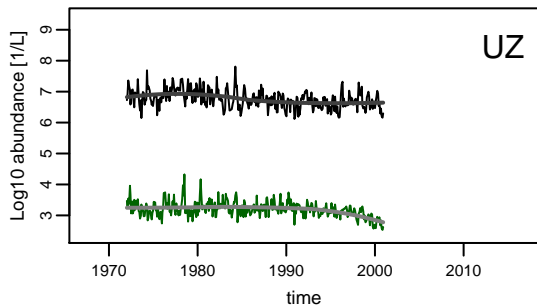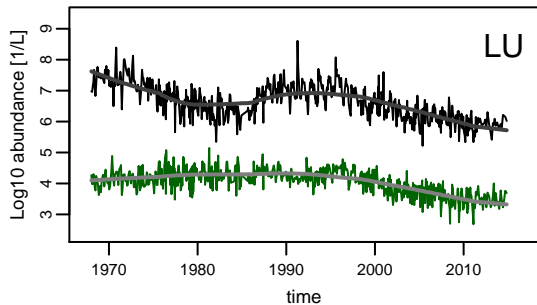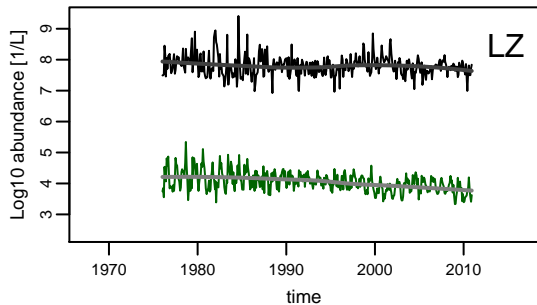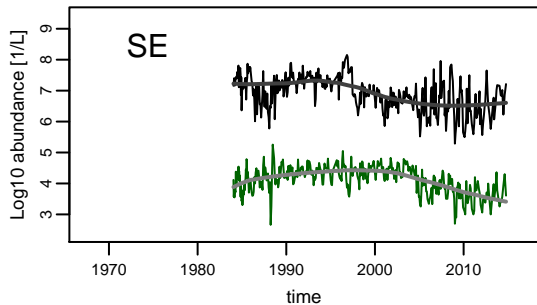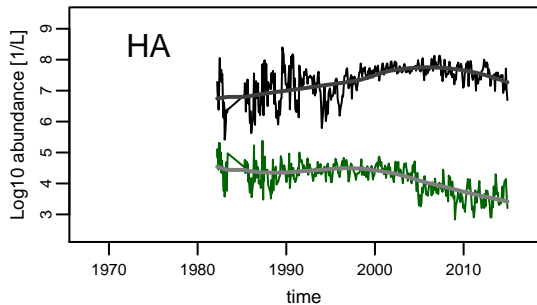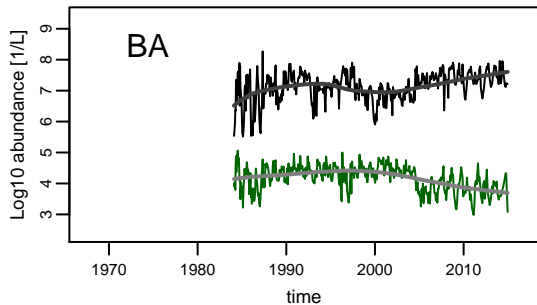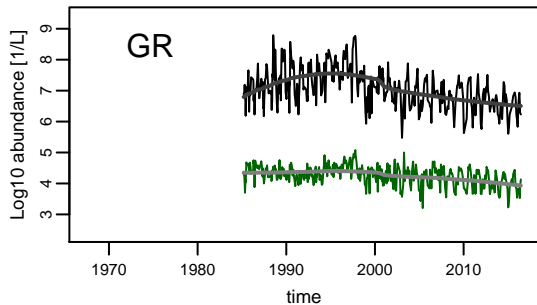

Supplement: FIGURE S1 — Scaling of phytoplankton abundances (Log10 cells L−1) with size (Log10 taxa biovolumes) in each lake dataset. [file Presentation_1.ZIP › FigS8.phyto_med&tot_abundances.pdf]

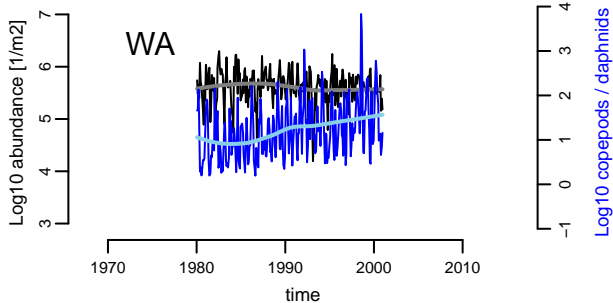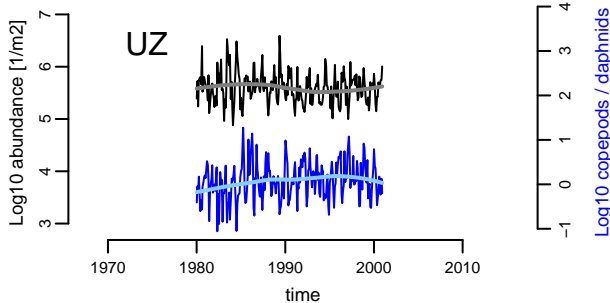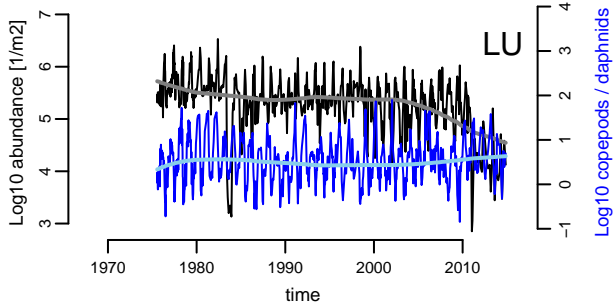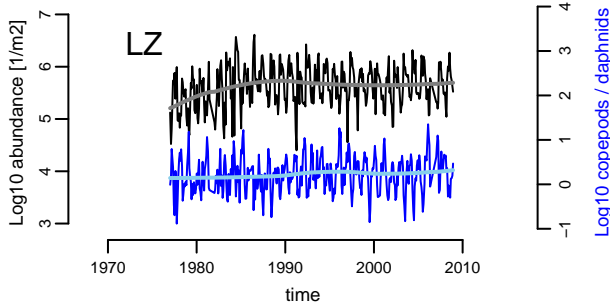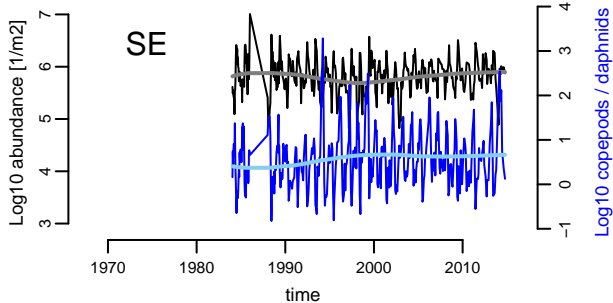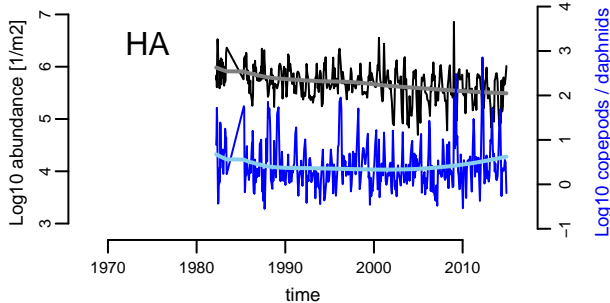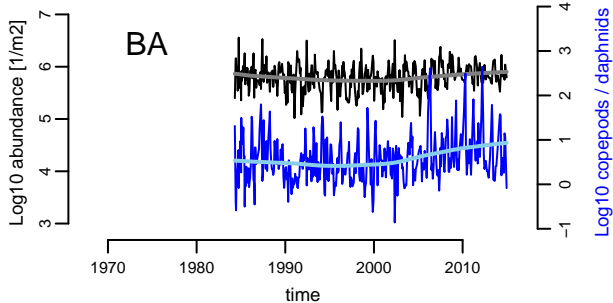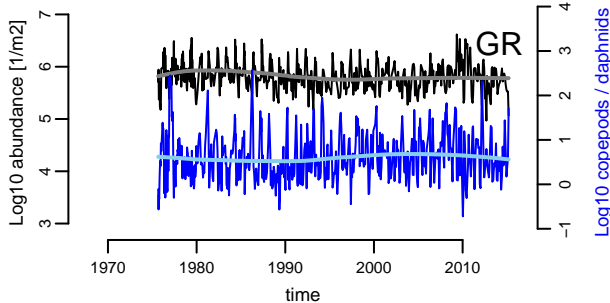

Supplement: FIGURE S1 — Scaling of phytoplankton abundances (Log10 cells L−1) with size (Log10 taxa biovolumes) in each lake dataset. [file Presentation_1.ZIP › FigS9.zooplankton.pdf]
